# From START to FINISH: The Influence of Osmotic Stress on the Cell Cycle

**DOI:** 10.1371/journal.pone.0068067

**Published:** 2013-07-10

**Authors:** Elahe Radmaneshfar, Despoina Kaloriti, Michael C. Gustin, Neil A. R. Gow, Alistair J. P. Brown, Celso Grebogi, M. Carmen Romano, Marco Thiel

**Affiliations:** 1 Institute for Complex Systems and Mathematical Biology, SUPA, University of Aberdeen, Aberdeen, United Kingdom; 2 Institute of Medical Sciences, Foresterhill, University of Aberdeen, Aberdeen, United Kingdom; 3 Department of Biochemistry and Cell Biology, Rice University, Houston, Texas, United States of America; Texas A&M University, United States of America

## Abstract

The cell cycle is a sequence of biochemical events that are controlled by complex but robust molecular machinery. This enables cells to achieve accurate self-reproduction under a broad range of different conditions. Environmental changes are transmitted by molecular signalling networks, which coordinate their action with the cell cycle. The cell cycle process and its responses to environmental stresses arise from intertwined nonlinear interactions among large numbers of simpler components. Yet, understanding of how these pieces fit together into a coherent whole requires a systems biology approach. Here, we present a novel mathematical model that describes the influence of osmotic stress on the entire cell cycle of *S. cerevisiae* for the first time. Our model incorporates all recently known and several proposed interactions between the osmotic stress response pathway and the cell cycle. This model unveils the mechanisms that emerge as a consequence of the interaction between the cell cycle and stress response networks. Furthermore, it characterises the role of individual components. Moreover, it predicts different phenotypical responses for cells depending on the phase of cells at the onset of the stress. The key predictions of the model are: (i) exposure of cells to osmotic stress during the late S and the early G2/M phase can induce DNA re-replication before cell division occurs, (ii) cells stressed at the late G2/M phase display accelerated exit from mitosis and arrest in the next cell cycle, (iii) osmotic stress delays the G1-to-S and G2-to-M transitions in a dose dependent manner, whereas it accelerates the M-to-G1 transition independently of the stress dose and (iv) the Hog MAPK network compensates the role of the MEN network during cell division of MEN mutant cells. These model predictions are supported by independent experiments in *S. cerevisiae* and, moreover, have recently been observed in other eukaryotes.

## Introduction

The cell cycle is the most fundamental biological clock underlying all forms of life. It enables faithful duplication of the entire set of genes before cell division, ensuring stable cell proliferation. The cell cycle can be considered as a sequence of biochemical events governed by a complex but robust molecular network. This network has evolved in a sophisticated way, allowing cells to achieve accurate self reproduction in various conditions. Environmental changes are transmitted by molecular signalling networks that allow cells to react accordingly. Signal transduction networks, however, do not work in isolation, but coordinate their action with the cell cycle machinery; allowing flexible timing of crucial cell cycle events, adapted to the type and level of stress. In the past, cell cycle and stress response networks have generally been studied in separation. It has recently become clear, however, that to understand cellular responses to stresses, cell cycle and signalling networks have to be considered simultaneously. Recent studies, particularly in the case of osmotic stress [1]–[5], have revealed some key links between stress response and cell cycle networks.

The molecular machinery, which regulates DNA replication and segregation, is highly conserved from unicellular eukaryotes to multicellular eukaryotes [6]. Therefore, simple eukaryotes, such as fission yeast and budding yeast, serve as convenient model organisms to understand the analogous cell cycle control mechanisms in metazoa including humans. To understand such a complex system we have developed a novel mathematical model which integrates the osmotic stress signalling pathway with the cell cycle control network of budding yeast, *S. cerevisiae*. First modelling approaches have addressed the interaction between the osmotic stress response and the G1 phase of the cell cycle of *S. cerevisiae* [5]. Yet, because, the cell cycle phases are linked by global control mechanisms, the effect of osmotic stress on the cell cycle cannot be predicted from the consideration of one single phase alone. In this paper we introduce a mathematical model that, for the first time, describes the effect of osmotic stress in all stages of cell cycle progression. Our mathematical model elucidates how this elaborate system might work in the presence of osmotic stress in *S. cerevisiae*. The model unveils that the influence of the osmotic stress on different stages of the cell cycle attributes to interaction among many components of the entire cell cycle network rather than a single element. It also provides a tool for further investigation of the molecular processes and cell behaviour of the budding yeast cells under various environmental conditions and experimental setups.

### Cell Cycle Regulation of *S. cerevisiae*

The cell cycle of eukaryotes consists of two main phases, the S (DNA Synthesis) phase and the M (Mitosis) phase, which are separated by the G1 (Gap1) and G2 (Gap2) phases. In the G1 phase, the cell substantially increases in size and prepares for the S phase, during which DNA replication occurs. The G2 phase provides the cell with additional time to grow and to activate regulatory mechanisms in preparation for cell division. During the M phase chromosome segregation and nuclear division take place, and the cell divides into two isogenic cells. The cell cycle has three main transitions (see Figure S1 in Supporting Information S1): the G1-to-S transition (START), the G2-to-M transition and the M-to-G1 transition (FINISH). Activity of Cyclin Dependent Kinases (CDKs) causes transitions between phases [7]. CDK activity (Cdc28 in *S. cerevisiae*) is regulated by the availability of its cyclin partners, inhibitory tyrosine phosphorylation (like phosphorylation of Cdc28 by Swe1) and binding to stoichiometric CDK inhibitors (like Sic1) [8]. Cdc28 has two types of associated cyclins: (i) the three G1 cyclins, (Cln1, Cln2, Cln3) and (ii) the six B-type cyclins (Clb1 to Clb6). G1 cyclins regulate events in the gap between mitosis and DNA replication, whereas B-type cyclins are expressed successively from START to FINISH [9]–[12]. Among the G1 cyclins, Cln3 links growth to the expression of Cln1 and Cln2 mediated by the transcription factor SBF (see reactions sr1 and sr2 in Figure S1 in Supporting Information S1). Cln1 and Cln2 are responsible for appearance of the bud [11]. As Cln1 and Cln2 act similarly [11] they are represented by Cln2 in our model. The six B-type cyclins are divided into three distinct pairs of similar functions. The cyclins Clb5 and Clb6 initiate the DNA synthesis [12]. These cyclins, also known as S phase cyclins, are both represented by Clb5 from now on. The mitotic cyclins Clb1 and Clb2 [10] are crucial for successful mitosis [9]. This pair is represented by Clb2 in our model. The remaining B-cyclins, Clb3 and Clb4, play a redundant role in initiating the S phase and also in mitotic spindle formation [8]. Therefore, we do not distinguish them in our model. Figure S1 in Supporting Information S1 shows the main molecular interactions that control the timing of the cell cycle. For further details of these molecular mechanisms, see Section 1.1 of Supporting Information S1.

### The Influence of Osmotic Stress on Cell Cycle Progression

**Figure 1:**
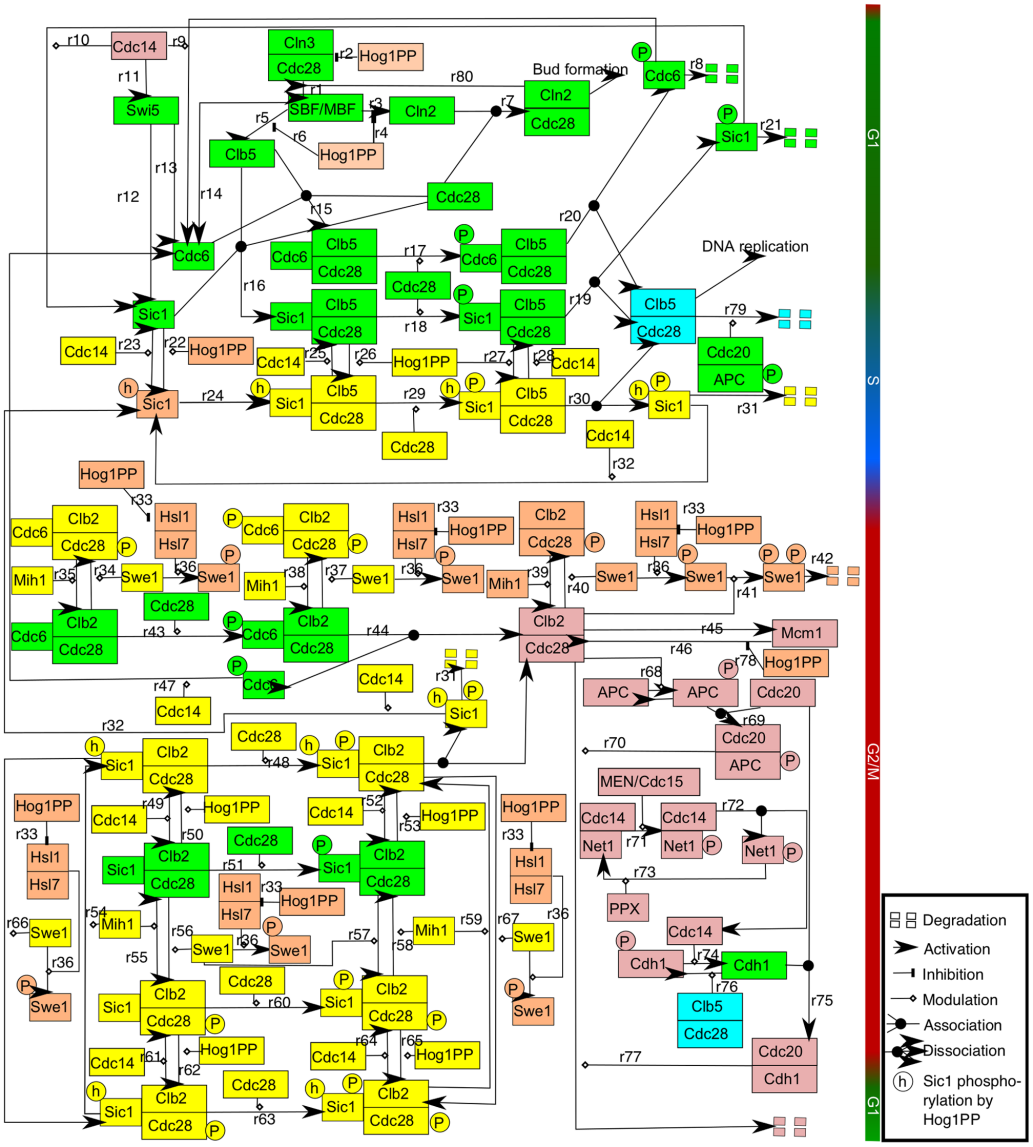
Wiring diagram of the interactions of Hog1PP with the cell cycle components. This network summarises the biological interactions (see text for details of these interactions) and modelling assumptions. Active Hog1PP halts cell cycle progression depending on the phase of the cell cycle at the onset of osmotic stress. The components marked in yellow and orange in this figure represent elements that couple the cell cycle with the osmotic stress response network. Components involved in known biological interactions are marked in orange and the ones involved in assumed interactions based on known experimental evidence are marked in yellow (see Introduction section for details of modelling assumptions). Cell cycle control components are coloured based on the phase of activity, namely, green for G1 phase, blue for S phase and pink for G2/M phase (see Section 1 of Supporting Information S1 for details of cell cycle control interactions, osmotic stress response and modelling assumptions). Note that some components, such as Hog1PP and Swe1, appear several times in the diagram. This has been done for clarity, because they are involved in multiple reactions.

The cycle of biochemical events is compromised in the presence of stress. Various receptors sense osmotic stress and activate different signalling pathways, among which the High-Osmolarity Glycerol (HOG) MAPK signalling plays a key role [13], [14]. The MAPK signalling module is highly conserved among eukaryotes [14]. Activation of the HOG MAPK signalling network by an increase in the osmolarity of the cell environment results in the activation of Hog1 via phosphorylation (see Figure S2 in Supporting Information S1) [13]. Dually phosphorylated Hog1 (Hog1PP) then accumulates in the nucleus and activates gene expression of proteins involved in the recovery of the cell from osmotic stress. In the presence of osmotic stress the cell cycle progression is delayed. In the last decade, various interactions between Hog1PP and different cell cycle regulated proteins have been experimentally identified which can account for the observed delay in cell cycle progression [1]–[4], [15]. Importantly, the mechanisms of interaction between the osmotic stress response and the cell cycle machinery depend on the phase of the cell cycle during which the osmotic stress is applied. Figure 1 shows a schematic diagram summarising the main interactions between Hog1PP and cell cycle regulators (note that some components, such as Hog1PP and Swe1, appear several times in the diagram; this has been done for clarity, because they are involved in multiple interactions).

In untreated cells the G1-to-S transition is triggered by the activation of the Cdc28-Cln2 complex which enables Cdc28 to target Sic1 (CKI) for degradation (see reactions r17 and r18 in Figure 1), thereby freeing the S phase cyclin to initiate DNA replication (see reactions r19 and r20 in Figure 1) [16], [17]. If osmotic stress is applied during the G1 phase, the activation of Hog1 results in a G1 arrest by a dual mechanism: (i) Hog1PP downregulates the transcription of the G1 cyclins [1], [2] (see reactions r2 and r4 in Figure 1), and (ii) Hog1PP phosphorylates Sic1 directly on a specific site (see reaction r22 in Figure 1) [2]. This alters the efficient degradation of Sic1, thereby stabilising this protein [2]. Hence, the G1-to-S transition is transiently blocked in response to osmotic stress [2].

The next cell cycle transition, G2-to-M, is mainly governed by the activity of Cdc28-Clb2 [9], which is regulated by several mechanisms. The protein kinase Swe1 inhibits Cdc28-Clb2 activity by tyrosine phosphorylation of Cdc28 during the G1 and S phase (see reaction r40 in Figure 1), but it disappears during the G2-to-M transition (see reactions r36, r41 and r42 in Figure 1) [18]. Then, the freed Cdc28-Clb2 activates Mcm1 [19], which is the transcription factor of *CLB2*, thereby establishing a positive feedback loop (see reactions r45 and r46 in Figure 1). Active Cdc28-Clb2, therefore, transfers the cell to the M phase, during which the replicated chromosomes are segregated. The activity of Hog1PP also restricts the G2-to-M transition due to two main mechanisms [3], [15]: (i) accumulation of Swe1, and (ii) downregulation of *CLB2* expression (see reaction r78 in Figure 1). Rapid degradation of Swe1 is regulated by the activity of the Hsl1-Hsl7 complex as well as by the activity of Cdc28-Clb2 in an untreated cell (see reactions r36, r41 and r42 in Figure 1) [20]. However, in the presence of osmotic stress, Hog1PP targets Hsl1 for phosphorylation, hindering the Hsl1-Hsl7 complex formation (follow all reactions named r33 in Figure 1). As a consequence, Swe1 is not degraded [3] and Cdc28-Clb2 activity is inhibited. This together with the direct downregulation of *CLB2* transcription, leads to a G2 arrest. Moreover, the presence of osmotic stress delays S phase progression by direct downregulation of *CLB5* transcription by Hog1PP (see reaction r6 in Figure 1) [4]. Based on all these interactions we have built the wiring diagram depicted in Figure 1. Cell cycle control components are coloured based on the phase in which they are active, namely, green for G1, blue for S and pink for G2/M. Furthermore, the cell cycle regulated components involved in interactions with Hog1PP – which are well stablished in literature [1]–[4], [15] – are indicated in orange, whereas the components involved in interactions with Hog1PP which are hypothesised by us – based on reported experimental data [21]–[26] – are indicated in yellow in Figure 1. Next, we explain the hypothesised interactions in the model.

### Hypothesised Interactions in the Model

First, we assume that Hog1PP can phosphorylate Sic1 when the latter is in any of its forms, that means, both when Sic1 is in a complex or when it is unbound (see reactions r26, r50 and r62 in Figure 1). We also assume that Hog1 can phosphorylate Sic1 when it is already phosphorylated by Cdc28 (see reactions r27, r53 and r65 in Figure 1). Since Sic1 has 9 phosphorylation sites [21] and the site at which Hog1PP phosphorylates Sic1 is different from the site at which Cdc28 phosphorylates Sic1 [2], this assumption is very plausible. Hence, it is also reasonable to assume that Sic1 can be first phosphorylated by Hog1PP and then by Cdc28, both when Sic1 is free and when it is bound in a complex (see reactions r29, r48 and r63 in Figure 1). Cdc28-phosphorylated Sic1 is denoted by Sic1P and Hog1PP-phosphorylated Sic1 is denoted by Sic1h in the equations of the model and in Figure 1. Moreover, given the experimental evidence that Sic1h has a reduced binding affinity to the Cdc4 complex [2], Sic1h is assumed to be more stable than Sic1P in our model, both in free and complex form.

Second, we assume that if Sic1 is first phosphorylated by Hog1PP, then it can also bind to the Cdc28-B-type cyclins complexes (note that although these associations are considered in the mathematical model, they have not been depicted in Figure 1 for clarity). Furthermore, we assume that Sic1h can be dephosphorylated by the phosphatase Cdc14 (see reactions r23, r25, r28, r32, r49, r52, r61 and r64 in Figure 1), since Cdc14 also dephosphorylates Sic1P and it is known to dephosphorylate almost all substrates involved in the G1-to-S transition [22].

Third, it is known that Swe1 phosphorylates and inhibits Cdc28-Clb2 [27]. Likewise, we assume that Swe1 can phosphorylate any complex containing Cdc28-Clb2 (see reactions r34, r37, r56, r57, r66 and r67) [23]. Note that Swe1, after it has been phosphorylated by Hsl1-Hsl7, it is hyperphosphorylated by Cdc28-Clb2 and consequently degraded. This has been considered in the mathematical model but it is not shown in Figure 1 for clarity, i.e. reaction r36 is always followed by reactions r41 and r42 in the mathematical model.

Our mathematical model incorporates all these interactions through a set of 54 ordinary differential equations and 35 algebraic equations, and it yields novel predictions and provides the mechanisms underlying unexplained experimental results previously reported in the literature. One of the main predictions of the model is a second incidence of DNA replication before mitosis, when osmotic stress is applied during late S or early G2/M phase. This is a novel result that has not been reported yet. It is, however, strongly supported by the dual role of the S phase cyclin on DNA replication [28], [29]. Dahmann *et al.* and Nguyen *et al.* observed that downregulation of S phase cyclin before upregulation of M phase cyclin causes DNA re-replication [28], [29]. According to our model Hog1PP activity also downregulates S phase cyclin and results in DNA re-replication before cell division. Our integrative model also provides a mechanistic explanation for experimental results reported by Reiser *et al.* regarding the exit from mitosis of certain mutated cells in the presence of osmotic stress [30]. Mutation of Mitotic Exit Network (MEN) components arrests the cells in the G2/M phase. However, these cells are able to exit mitosis if an osmotic stress is applied [30]. Our model does not only reproduce this experimental result but it also provides a mechanism responsible for this effect. In fact, the MAP kinase Hog1, which is activated by osmotic stress, stabilises the CDK inhibitor Sic1 and thereby the transition to the new G1 phase is facilitated. Furthermore, our model predicts that osmotic stress delays the G1-to-S and G2-to-M transitions in a dose dependent manner, whereas it accelerates the M-to-G1 transition independently of the stress dose. We therefore present a series of novel predictions of what constitute the most complete model for the reaction of budding yeast cell cycle to osmotic stress.

## Results

### Osmotic Stress Delays the G1-to-S and G2-to-M Transitions

The osmostress-activated MAP kinase Hog1 modulates the activity of several components of the cell cycle network to prevent cell cycle progression before proper adaptation to the osmotic stress. According to our model and in accordance with experimental observations the duration of osmostress-induced cell cycle arrest depends on the position of the cell in its cycle at the onset of the osmotic stress. To demonstrate this, we apply osmotic stress at different points of our simulated cell cycle and calculate the arrest duration by:
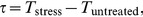
(1)where 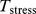 denotes the cell cycle duration under stress and 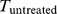 is the cell cycle duration of untreated cells.

**Figure 2:**
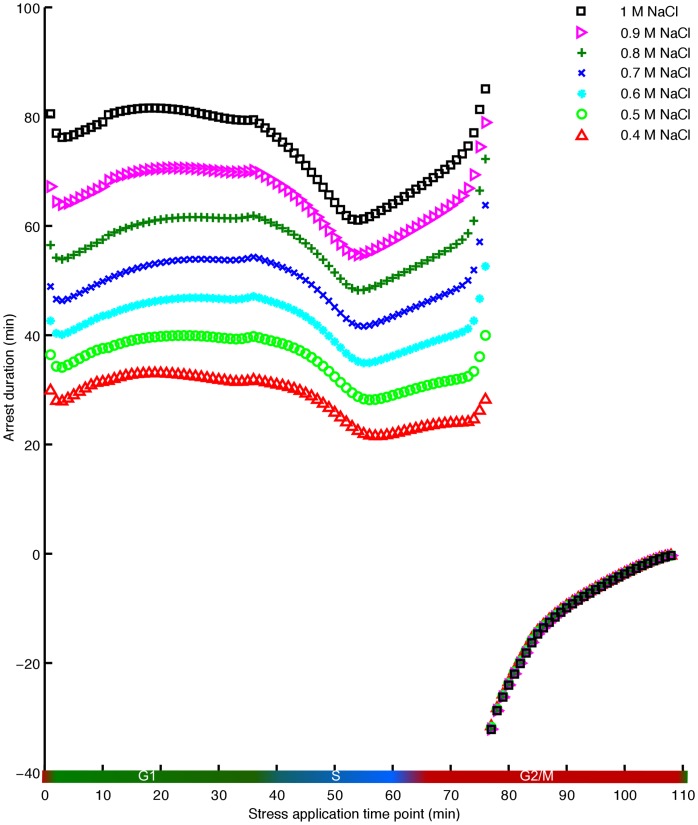
Dose-dependent arrest duration following the imposition of osmotic stress at different stages of the cell cycle. The x-axis represents the time point of application of stress, whereas the y-axis illustrates the corresponding arrest duration. Different colours demonstrate various doses of the stress, ranging from 0.4 M NaCl to 1 M NaCl. During the G1 phase and the S phase, higher doses of stress cause longer cell cycle arrests, while the acceleration of the exit from mitosis is dose independent.

Figure 2 shows simulation results of the arrest duration 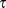 throughout the cell cycle for different doses of NaCl. Strikingly, according to our model there are two distinct types of cell cycle responses to NaCl depending on the timing of stress:

(i) Before the transition to the M phase, 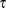 is positive, i.e. that the cell cycle progression becomes slower (see Figure 2). This result has recently been experimentally validated for 0.4 M NaCl at few time points before the transition to the M phase [1]–[4], [15]. Moreover, the value of 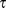 depends on the time point at which the stress is applied. For example, the cell cycle arrest reaches a minimum duration within the S phase and increases again at the beginning of the G2 phase.

(ii) Immediately after the beginning of the FINISH process, instead of having a delay, the progression of the cell cycle is accelerated (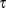 becomes negative). Note that the beginning of the FINISH process is defined as the time point at which Mcm1 (M phase cyclin transcription factor) reaches its maximal value [31]. Cells exposed to osmotic stress at the beginning of the FINISH or later have an accelerated M-to-G1 transition and get arrested in the G1 phase of the next cell cycle. Remarkably, the transition in 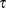 is very sharp. The stress induced arrest duration changes suddenly from being close to 40 min to approximately −35 minutes for 0.5 M NaCl (green circle in Figure 2). The later the stress is applied after FINISH, the smaller becomes 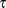 in magnitude and the cell cycle duration becomes closer to the cell cycle duration of the untreated cell.

**Figure 3:**
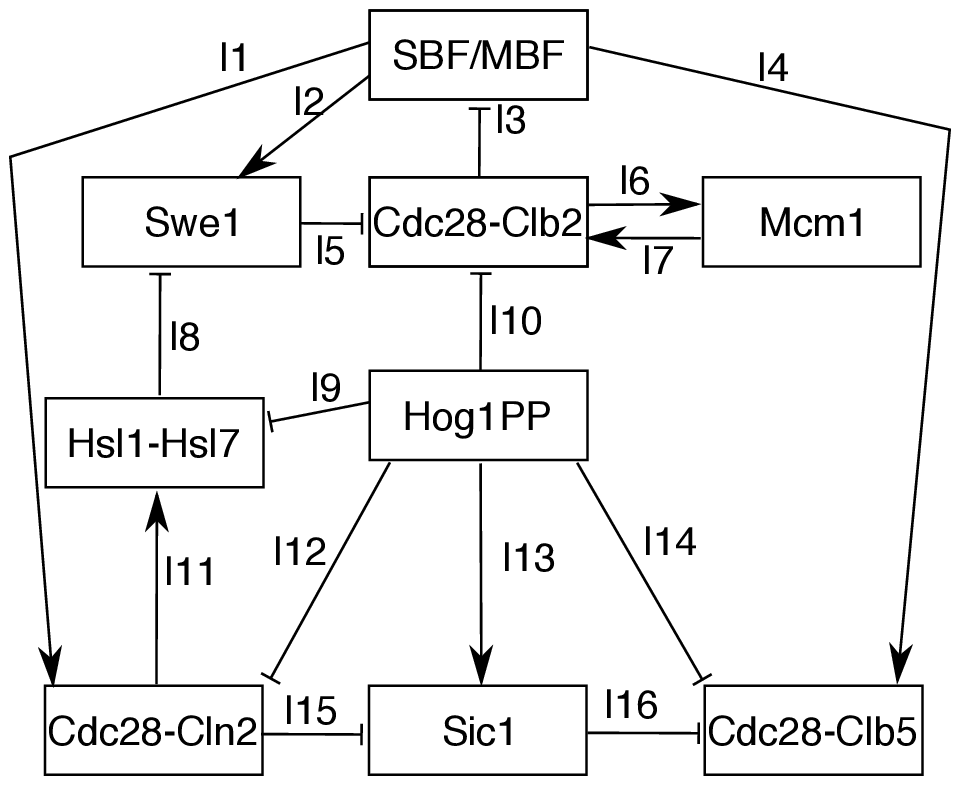
Summary of interactions of Hog1PP with cell cycle components. Hog1PP phosphorylates Sic1, which results in accumulation of Sic1 and an arrest in G1 phase. Also, Hog1PP prevents the formation of the Hsl1-Hsl7 complex by phosphorylating Hsl1. This leads to the accumulation of Swe1, due to the double negative link between Hog1PP and Swe1. As a consequence, the cell is arrested before the G2/M transition. Transcriptional downregulation of *CLN2* by Hog1PP influences the cell cycle progression in two different ways: first, it causes Sic1 to be less phosphorylated and more active. This inhibits the cell cycle transition to the S phase. Second, it influences indirectly the timing of the formation of the Hsl1-Hsl7 complex, which is important for the transition to the G2/M phase. Moreover, the downregulation of Cdc28-Clb2 by Hog1PP can cause SBF/MBF to be active for an extended duration, and therefore, cell cycle arrest before the G2/M transition.

Our model elucidates the molecular mechanisms responsible for the osmotic stress induced delay across the different cell cycle phases. Figure 3 summarises the key interactions between Hog1PP and the cell cycle components shown in Figure 1, emphasising the key reactions involved in cell cycle adaptation to osmotic stress, as inferred by our model.

**Figure 4:**
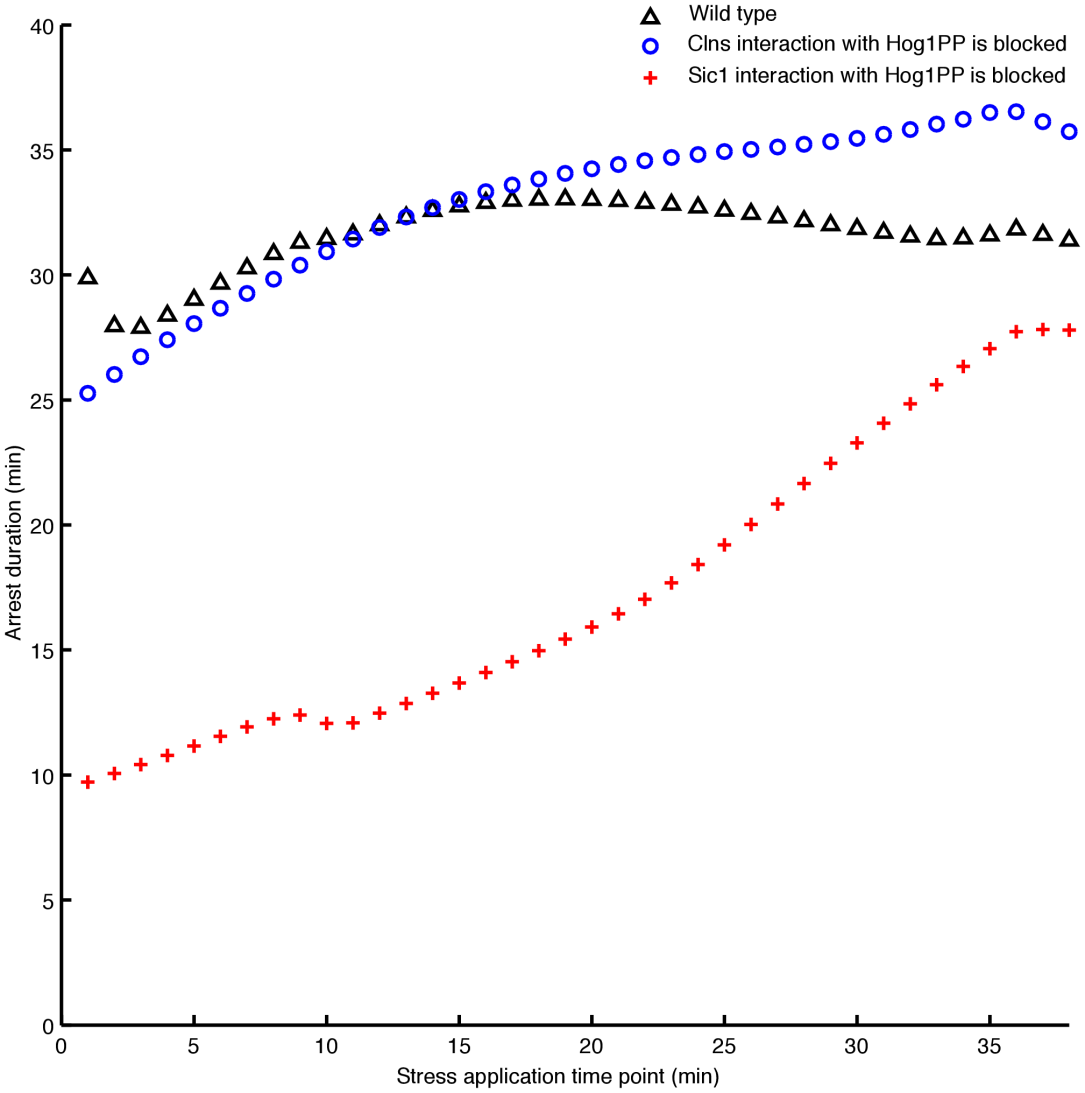
Assessing the role of two key mechanisms responsible for the cell adaptation to osmotic stress during the G1 phase. The x-axis represents the time point of application of stress, whereas the y-axis illustrates the corresponding arrest duration. Blocking the interaction of Sic1 with Hog1PP, reduces the arrest duration significantly along the G1 phase (red crosses).

During the G1 phase, the experimentally reported mechanisms responsible for the delay are (i) the Sic1 stabilisation mediated by Hog1PP (see link l13 in Figure 3) and (ii) the transcription downregulation of G1 cyclins due to Hog1PP (see link l12 in Figure 3) [1], [2]. To assess the importance of the first mechanism along the entire G1 phase, we block the interaction of Sic1 with Hog1PP in our model (see Figure 4). Note that to implement this experimentally, the specific phosphorylation site of Sic1 needs to be blocked [2]. In this case 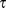 strongly decreases, especially at the beginning of the G1 phase, compared with the respective arrest duration in the wild type (red crosses in Figure 4). At the end of G1 phase, the role of Sic1 becomes less dominant, as the level of Sic1 decreases. In contrast, if we remove the influence of Hog1PP on the transcription of G1 cyclins from the model, we observe a small difference in the delay 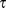 compared with the wild type cell (blue circles in Figure 4). Hence, according to our analysis Sic1 stabilisation by Hog1PP plays an essential role in the G1 phase arrest. The regulation of Cdc28-Cln2 and Sic1 upon activation of Hog1PP during G1 are shown in Figures S5B and S5C in Supporting Information S1.

**Figure 5:**
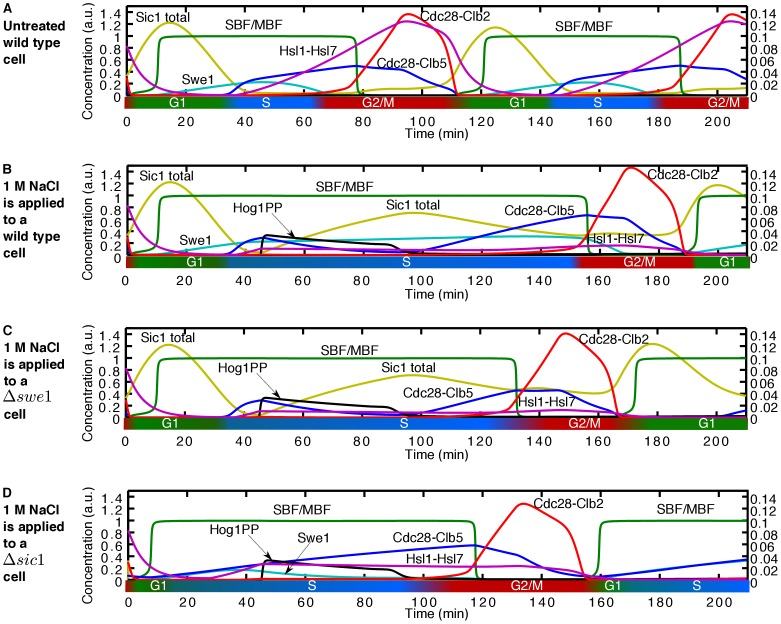
Time course activity of cell cycle components upon application of 1 M NaCl at early S phase. The left vertical axis refers to the concentrations of total Sic1, SBF/MBF, Swe1, Cdc28-Clb2, Cdc28-Clb5, and Hog1PP and the right vertical axis refers to the concentration of the Hsl1-Hsl7 complex. (A) A wild type untreated cell, (B) 1 M NaCl applied during early S phase (at t = 45 min) to a wild type cell causes the cell cycle to last about 76 minutes longer compared to the wild type untreated cell. (C) 1 M NaCl applied to a Δ*swe1* cell; in this case the cell cycle duration is 62 minutes longer than in an untreated Δ*swe1* cell. (D) The deletion of Sic1 does not cancel the delay caused by Hog1PP activity. 1 M NaCl applied to a Δ*sic1* cell prolongs the cell cycle around 52 minutes compared to a Δ*sic1* untreated cell.

During the early S phase, our model predicts that the slow progression of the cell cycle is mainly due to the delayed accumulation of Cdc28-Clb5. In Figure 5 we show the time course activity of cell cycle components playing a key role during early S phase upon activation of 1 M NaCl. First, the downregulation of *CLB5* transcription by Hog1PP causes the Cdc28-Clb5 level to decrease initially (see blue line in Figure 5B). However, after Hog1PP returns to its basal level, our model predicts a further increase of Cdc28-Clb5 by the active SBF/MBF transcription factors (compare the activity of Cdc28-Clb5 – blue line – in Figure 5B with Figure 5A, and see link l4 in Figure 3). The SBF/MBF transcription factors, in turn, remain high for an extended time interval due to Hog1PP mediated stabilisation of Swe1. The accumulation of Swe1 prevents the increase of Clb2 activity, which is the main inhibitor of SBF/MBF. Moreover, SBF/MBF activates the transcription of Swe1, establishing a positive feedback mechanism (links l2, l5, and l3 in Figure 3), causing the maximum level of Swe1 to be higher than in the unstressed cell (compare the black line – untreated cell – in Figure S5F in Supporting Information S1 with other colour lines). Only when the level of the Hsl1-Hsl7 complex increases, the feedback mechanism (links l2, l5, and l3 in Figure 3) becomes less efficient, enabling the transition to the late S phase. Therefore, the positive feedback between the SBF/MBF and Swe1 via Cdc28-Clb2 causes the delayed accumulation of Cdc28-Clb5 and as a result a longer S phase. The stress-induced delay during early S phase shows a steady decrease (see Figure 2). This is due to the higher level of the Hsl1-Hsl7 complex as we move towards the late S phase, which makes the positive feedback loop between SBF/MBF and Swe1 less effective. Consequently, SBF/MBF is active for a shorter period of time and, therefore, the delay decreases. Hence, the positive feedback between the SBF/MBF and Swe1 via Cdc28-Clb2 (links l2, l5, and l3 in Figure 3) not only causes a longer early S phase but also controls the length of this delay.

Yaakov *et al.* examined the role of Swe1 in the S phase delay caused by osmotic stress [4], and they found that a strain lacking Swe1 has almost the same delay during S phase as the wild type. To validate our model, we perform a simulation where we remove Swe1, and, in accordance with the experimental results, obtain a delay during the S phase that differs only by approximately 15 minutes compared with the wild type (compare Figure 5C with Figure 5B). Yaakov *et al.* also tested the role of Sic1 in the S phase delay [4]. They showed that Sic1 has almost no influence on the delay of the cell cycle progression during the S phase [4]. Our model also reproduces this result (compare Figure 5D with Figure 5B). Therefore, our model suggests that the S phase delay due to osmotic stress cannot be attributed to a single component but rather it emerges as the result of the interaction among the many components of the cell cycle network. We summarise the emergent molecular mechanisms as a consequence of the interaction between the cell cycle and stress response in Figure 3. Note that this concise network is derived based on a systematic study of the role of different components in reaction of the cell cycle to osmotic stress.

If the stress is applied during late S phase or at the beginning of G2/M phase, a striking phenomenon occurs leading to a second incidence of DNA replication before cell division. We discuss this in a dedicated section below.

### Osmotic Stress Causes Accelerated Exit from Mitosis

**Figure 6:**
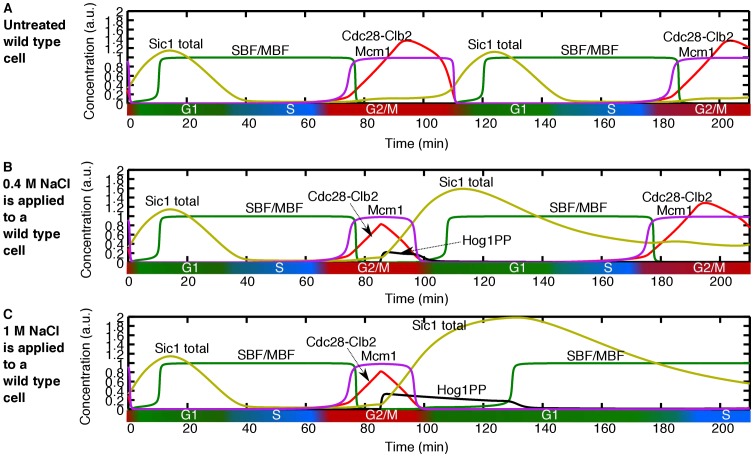
Time course activity of cell cycle components upon application of 0.4 M and 1 M NaCl at M phase. (A) Wild type untreated cell. (B) 0.4 M NaCl applied during G2/M phase to a wild type cell. Osmotic stress causes the cell to finish the current cell cycle very rapidly compared to untreated cell and gets arrested in the G1 phase of the next cell cycle. (C) 1 M NaCl applied during the G2/M phase. The time profile activity of the G2/M phase is the same for 0.4 M NaCl. This cell experiences the same accelerated exit from mitosis and gets arrested in the G1 phase of the new cell cycle. In this case the G1 phase is longer compare to the cell treated with 0.4 M NaCl.

In contrast to the cases discussed above, if osmotic stress is applied after a very precise time point in the G2/M phase, the cell experiences an accelerated exit from mitosis (discontinuity in arrest duration in Figure 2). The time point at which this dramatic change occurs is determined by the point at which the level of Mcm1, the transcription factor of *CLB2*, reaches its maximum. This time point coincides with the initiation of the FINISH process [31]. Hence, if the osmotic stress is applied at that time point or later, our model predicts that the level of Cdc28-Clb2 starts to decrease immediately mainly due to two mechanisms: (i) inhibition of Cdc28-Clb2 activity by Sic1, the latter being stabilised by Hog1PP (see Figure 3) and, (ii) direct transcriptional inhibition of *CLB2* by Hog1PP. Hence, the point at which Cdc28-Clb2 starts decreasing occurs earlier than in the absence of stress (compare Figure 6B with Figure 6A). Moreover, the level of Sic1 starts increasing rapidly due to the presence of Hog1PP, and as a consequence, the exit from mitosis is significantly accelerated. Also, the later the stress is applied during the M phase, the slower the acceleration becomes, since the exit from mitosis is further advanced (see Figure 2).

Importantly, after the accelerated exit from mitosis, the cells get arrested in the next G1 phase. Depending on the dose of the stress, the delay can be carried over to the subsequent cell cycles (compare Figure 6C – 1 M NaCl – with Figure 6B – 0.4 M NaCl). The effect of the stress dose is discussed in the next section.

### Delays in the G1-to-S and G2-to-M Transitions are dose Dependent, Whereas Acceleration of the M-to-G1 Transition is dose Independent

Next we apply different stress doses, ranging from 0.4 M NaCl to 1 M NaCl, at different times along the entire cell cycle, following the approach described in the last section. Upon the onset of the stress, Hog1PP rises almost immediately, stays active for a time interval proportional to the stress dose, and then returns rapidly to its basal level (Figures S6A in Supporting Information S1). Notably, if the stress is applied before FINISH, the delay obtained increases approximately linearly with the stress dose, whereas if the stress is applied after that point, the acceleration does not depend on the stress dose (see Figures 2 and S6A–I in Supporting Information S1).

According to our model, one of the key mechanisms responsible for this very different behaviour, is the regulation of the main transcription factors of the G1/S and G2/M phases, namely, SBF/MBF and Mcm1, respectively. The transcription complexes SBF/MBF are downregulated by Cdc28-Clb2, the latter being downregulated by Hog1PP (see Figure 3). Hence, upon stress, SBF/MBF are activated for an extended period of time and the main G1 and S phase cyclins are stabilised until Hog1PP returns to its basal level. Therefore, the delay in the cell cycle progression increases with the stress dose.

In contrast, Mcm1 is upregulated by Cdc28-Clb2, and upon stress, Cdc28-Clb2 is downregulated; as a consequence, the time interval during which Mcm1 is active, is reduced. Therefore, there is no stabilising influence on Cdc28-Clb2 upon stress, and its level decreases almost immediately after the onset of the stress (Figure S5H in Supporting Information S1), nearly independent of the stress dose (between 0.4 M and 1 M NaCl, see Figure S6H in Supporting Information S1). However, the next cell cycle will be delayed since Hog1PP is still active. The length of the delay in the next cell cycle depends on the stress dose (compare Figure 6B with 6C).

### Osmotic Stress at Late S and Early G2/M Phase Causes DNA Re-replication

**Figure 7:**
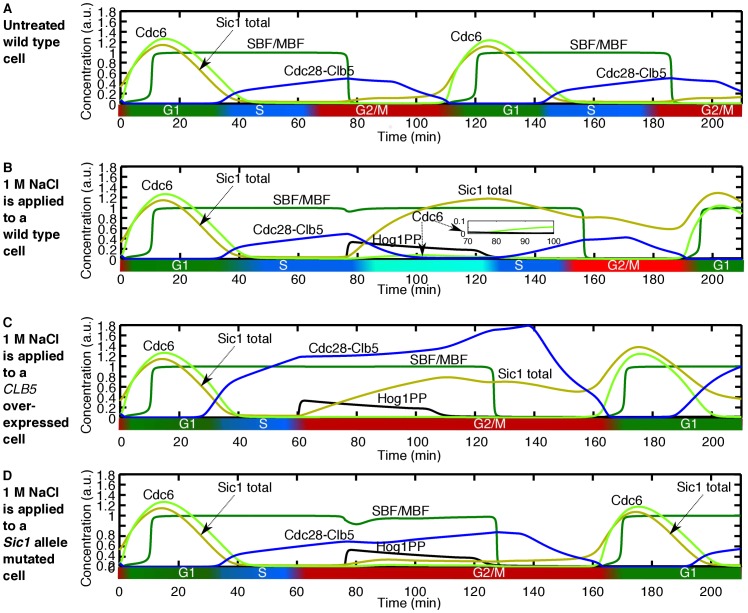
Application of osmotic stress during late S phase or early G2/M phase causes DNA re-replication. (A) Time course activity of the cell cycle components for the wild type untreated cell. (B) 1 M NaCl applied at minute 76. Activity of Hog1PP causes downregulation of Cdc28-Clb5. In addition, the level of Cdc6 slightly increases when Cdc28-Clb5 activity is reduced by Hog1PP (see inset). Then, after Hog1PP returns to its basal level, Clb5 starts increasing again. The downregulation, following by an upregulation of Cdc28-Clb5 can lead to DNA re-replication. (C) Overexpression of *CLB5*, by simulating induction of *CLB5* transcription from the GAL1 promoter, inhibits the DNA re-replication. (D) Blocking the interaction of Sic1 with Hog1PP also hinders the DNA re-replication in the presence of 1 M NaCl.

By simulating the application of osmotic stress in late S phase or early G2/M phase, our model predicts the initiation of a second incidence of DNA replication (Figure 7B). This effect is more pronounced for higher stress doses (Figure S7 in Supporting Information S1).

This prediction can be validated based on the experimental evidence reported on the dual role of Cdc28-Clb5 [28], [29]; this cyclin dependent kinase complex is responsible for both the initiation of DNA replication at the onset of the S phase, and the blocking of the assembly of the pre-replicative complex during the G2/M phase [28]. Blocking the assembly of the pre-replicative complex prevents DNA re-replication, thereby enabling stable propagation of genetic information [29], and it occurs via three overlapping mechanisms to prevent DNA re-replication: first, Cdc28-Clb5 reduces Cdc6 levels through phosphorylation; second, it promotes the nuclear export of MCM proteins, and third, it phosphorylates ORC proteins [29]. All three mechanisms render the replication origins in the post-replicative state, so that the high level of Cdc28-Clb5 prevents *de novo* assembly of the pre-replicative complex in the G2/M phase [29]. In order to show that the activity of Cdc28-Clb5 is crucial to prevent DNA re-replication, Dahmann *et al.* isolated mutations of the *SIM* genes that caused a second incidence of DNA replication without mitosis [28]. They demonstrated that mutated *SIM* genes lower the activity of Cdc28-Clb5, probably by a post-transcriptional mechanism. To validate the key role of Cdc28-Clb5 activity for the prevention of DNA re-replication, they overexpressed *CLB5* in the *SIM* mutant in G2-phase arrested cells and DNA re-replication was inhibited. Moreover, in another experiment they inhibited Cdc28-Clb5 activity by inducing Sic1 expression during the G2/M phase, which led to the assembly of the pre-replicative complex. Subsequent repression of Sic1 allowed recovery of Cdc28-Clb5 activity and, crucially, it triggered DNA re-replication. Therefore, by lowering the activity of Cdc28-Clb5, the three DNA re-replication blocking mechanisms are rendered ineffective.

Interestingly, our model predicts that, by applying osmotic stress during late S or early G2/M phase, the activity of Cdc28-Clb5 is decreased (Figure 7B). Just before applying the stress, at that stage of the cell cycle, Cdc28-Clb5 has reached a high level, and DNA replication is almost complete. If the osmotic stress is applied at that moment, initially Cdc28-Clb5 activity decreases, due to the downregulation of *CLB5* transcription by Hog1PP. Moreover, Hog1PP stabilises Sic1, which also inhibits Cdc28-Clb5 activity. Furthermore, Hog1PP also downregulates *CLB2* expression and, as a consequence, the transcription factors SBF/MBF remain active for longer. Accordingly, the levels of Cdc6 and Cdc14 increase, leading to the assembly of the pre-replicative complex (see Figures 7B and S7C in Supporting Information S1). Note that Cdc6 increases very slightly (see inset within Figure 7B and see Figure S7C in Supporting Information S1 for a higher resolution), but there is experimental evidence that low Cdc6 levels are sufficient to licence the origin of replication and transfer the cell to the S phase [32].

Then, after Hog1PP returns to its basal level, Cdc28-Clb5 starts increasing again, leading to a second peak in Cdc28-Clb5 activity (compare Figure 7B with 7A). This sequence of events therefore strongly suggests that a second incidence of DNA replication occurs before mitosis upon application of osmotic stress during late S or early G2/M phase. This novel prediction is in accordance with the experimental results mentioned above, which show that a decrease followed by an increase in Cdc28-Clb5 activity leads to DNA re-replication.

In order to further validate our model with the known measured data, we numerically overexpress *CLB5* by simulating induction of *CLB5* transcription from the GAL1 promoter [33]. In accordance with the experimental observation by Dahmann *et al.* [28], this overexpression inhibits DNA re-replication (Figure 7C). Moreover, to test the mechanism that we propose for DNA re-replication, we block the interaction of Sic1 with Hog1PP. This case leads to inhibition of DNA re-replication (Figure 7D). Hence, according to our model stabilisation of Sic1 by Hog1PP can lead to DNA re-replication for cells which are in the late S or early G2/M phases at the onset of application of osmotic stress.

### The HOG MAPK Network Can Supplant MEN Network’s Role in Cell Division

Our integrative cell cycle and osmotic stress model also provides a mechanistic explanation for the experimental results obtained by Reiser *et al.* [30] regarding the response of *cdc15
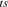
* cells to osmotic stress.

**Figure 8:**
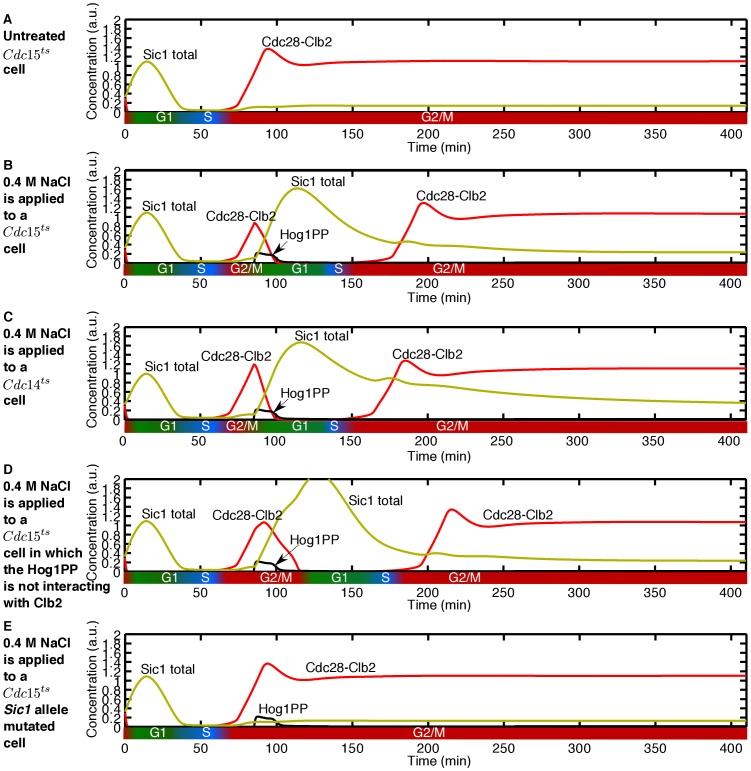
The HOG MAPK network rescues the mitotic exit defect of MEN mutants. (A) A *cdc15
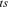
* cell is arrested in M phase and cannot divide. (B) Application of 0.4 M NaCl stimulates the *cdc15
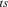
* cell to go through cell division. (C) The *cdc14
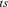
* cell can go through the cell division in the presence of 0.4 M NaCl. (D) Removing the interaction of Hog1PP with *CLB2* does not cancel the cell division of the *cdc15
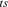
* cell in the presence of osmotic stress. Note that the *cdc15
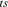
* cell upon osmotic stress is able to finish its current cell cycle but gets arrested in the next G2/M phase. (E) The *cdc15
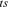
* cell, in which the interaction of Sic1 with Hog1PP is blocked, cannot finish its cell cycle and is arrested in M phase.

The protein kinase Cdc15 is one of the components of the Mitotic Exit Network (MEN) [34], responsible for the final M-to-G1 transition. For this transition to occur, Cdc28-Clb2 has to be inactivated, which is partly mediated by the protein phosphatase Cdc14 [35], [36]. In *S. cerevisiae*, Cdc14 is localised in the nucleolus during most of the cell cycle, but it is released during late M phase [37], [38], thereby reversing the activity of Cdc28-Clb2. Cdc14 delocalisation from the nucleolus is mainly controlled by MEN [39], [40]. Hence, *cdc15
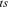
* cells, as well as other viable MEN temperature-sensitive mutant cells, keep Cdc14 trapped in the nucleolus, and consequently the levels of Cdc28-Clb2 activity remain high [35]. This leads to *cdc15
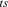
* cells being arrested in the M phase [41]. Figure 8A shows the simulation result for a *cdc15
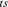
* cell. In accordance with the experimental results, the model predicts that *cdc15
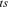
* cells do not divide [30].

A significant observation by Reiser *et al.* [30] was that the temperature-sensitive MEN mutant cells, at the non-permissive temperature, will complete mitosis and cell division following imposition of osmotic stress. It was suggested that the measured increase in Cdc14 activity, induced by the HOG MAPK network, was responsible for the M-to-G1 transition of the cell in the perturbed environment [30]. The molecular mechanism behind this experimental result, however, remained unclear.

By simulating the reaction of *cdc15
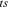
* cells to osmotic stress, we reproduce the known experimental results. Our simulations show that *cdc15
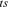
* cells, as well as further temperature-sensitive MEN mutants, end mitosis and enter a new cell cycle after exposure to various doses of osmotic stress, as measured in the experiments (see Figure 8B). In order to identify the mechanism responsible for this response, we first tested the role of Cdc14 in cell division in the osmotic stress condition. According to our model, *cdc14
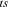
* cells can also go through cell division in the osmotically perturbed environment (see Figure 8C). The same result was experimentally obtained by Grandin *et al.* [41]. This suggests that Cdc14 upregulation by Hog1PP is not the key mechanism for cell division of MEN mutants in osmotic conditions.

We then tested the role of the downregulation of *CLB2* by Hog1PP in *cdc15
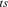
* cells by blocking this interaction in silico and applying the stress in the M phase. In this case the cell could complete mitosis and be transferred to the next cell cycle (see Figure 8D). Only by blocking the interaction between Hog1PP and Sic1, was possible to stop the cell from completing mitosis (see Figure 8E). Thus, stabilisation of Sic1 by Hog1PP is the key mechanism responsible for transferring MEN mutant cells under osmotic stress to the G1 phase of the next cell cycle.

### Sensitivity of the Predictions of the Model to Parameters

Our sensitivity analysis shows that the predictions of the model are robust against changes in the parameters (based on the analysis of the model predictions obtained with a large number of different sets of parameters changing over two orders of magnitude. For further details see Section 3 of Supporting Information S1). In particular, the predicted delays upon different stress doses did not show large changes upon variation of parameters.

Our analysis showed that the most sensitive parameter of the model was 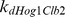, which quantifies the strength of inhibition of *CLB2* by Hog1PP. In fact, the predicted acceleration of cell division upon stress during the M-to-G1 transition depends strongly on this parameter. For example, when the value of 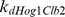 is reduced by approximately 60% from the estimated value, instead of predicting an acceleration in mitosis the cell is predicted to be arrested in the M phase (see Figure S8D and the corresponding caption in Supporting Information S1). It has been reported that overexpression of *CLB2* arrests the cell cycle in the M phase [42]. Note that a small value of 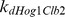 indicates weak inhibition of *CLB2* by Hog1PP, and therefore leads to a larger value of Clb2. Hence, the fact that the model prediction is sensitive to this parameter is supported by experimental data.

## Discussion

We have presented a novel model that describes how osmotic stress influences the cell cycle machinery throughout the entire cell cycle. Our model integrates recent experimental findings of the interaction of the osmotic stress response and cell cycle networks across different cell cycle phases. By considering the whole picture, rather than focusing on a special cell cycle phase, we are able to unveil mechanisms that emerge as a consequence of the multiple interactions between different parts of the cell cycle and osmotic stress response. Our model makes a series of novel predictions and provides mechanisms that explain further experimental findings which lacked explanation so far. The two main predictions of our model are: (i) upon osmotic stress in late S or early G2/M phase, cells undergo a second incidence of DNA replication before mitosis, (ii) cells stressed at late G2/M phase have an accelerated exit from mitosis and get arrested in the next cell cycle.

In non-stressed cells, DNA re-replication is prevented due to the inhibitory role of Cdc28-Clb5 in the assembly of the pre-replicative complex. When cells are osmotically stressed at the end of the S phase or early G2/M phase, however, the activity of Cdc28-Clb5 is downregulated by Hog1PP. This cancels the inhibition of Cdc28-Clb5 on the pre-replicative complex formation, and thereby causes increased activity of Cdc6 and Cdc14. Then, when Cdc28-Clb5 recovers, after Hog1PP returns to its basal level, a second incidence of DNA re-replication is initiated before mitosis. Note that the level of Cdc6 is lower than in the first incidence of DNA replication, but there is experimental evidence that low Cdc6 levels are sufficient to licence the origin of replication and transfer the cell to the S phase [32]. Importantly, our model identifies the mechanisms responsible for DNA re-replication; our results indicate that by blocking the Hog1PP interaction with Sic1, DNA re-replication should be inhibited.

The alternative prediction derived from our model is that Hog1PP may exert another, as yet undetected, level of control on licensing factors to prevent re-replication of DNA. Interestingly, in human cells the licensing protein Cdt1, which assists the assembly of the pre-replicative complex, is phosphorylated by the stress-activated mitogen-activated protein (MAP) kinases. Phosphorylated Cdt1 is then rapidly degraded, thereby inhibiting DNA re-replication upon osmotic stress [43]. In *S. cerevisiae* Cdt1 is also present, but to our knowledge, it has not been reported that it is phosphorylated by HogPP.

The second main prediction of the model, namely the accelerated exit from mitosis, has, to our knowledge, not yet been tested experimentally for *S. cerevisiae*. However, recent studies on the influence of osmotic stress on dividing leaf cells in the model plant *Arabidopsis thaliana* also shows early exit from mitosis, as our model predicts for budding yeast [44], [45]. If this prediction is not confirmed experimentally in *S. cerevisiae*, it would strongly indicate that a key component linking the M phase and osmotic stress response is missing; our model suggests that this component should hinder the Clb2 inactivation mechanisms to inhibit accelerated exit from mitosis.

Furthermore, we find that the cell cycle progression is delayed approximately linearly with stress dose if the cells are stressed at the G1, S or early G2/M phase. This linear increase of the delay with the stress dose has been experimentally observed by Adrover *et al.* for the cells which are at the G1 phase at the onset of stress [5]. In contrast, if cells are stressed at the late G2/M phase, they undergo an accelerated exit from mitosis in a stress dose-independent manner. This is due to the existence of a common mechanism which is responsible for the delay observed in cells stressed at the G1, S or early G2/M phase, even though the details differ from phase to phase. In all cases, interactions of Hog1PP with different cell cycle network components lead to an extended active interval of the transcription factor complexes SBF and MBF. In contrast, the activity of Hog1PP during the G2/M phase causes a reduced active time interval of the transcription factor Mcm1. In the case of cells stressed during the late G2/M phase, it is noteworthy that even though accelerated exit from mitosis is stress dose independent, they are arrested in the next cycle, and the duration of that arrest is indeed dose dependent.

Finally, our model provides a mechanism that explains why MEN (Mitotic Exit Network) temperature sensitive mutant cells undergo mitosis under osmotic stress. It has been suggested that *cdc15
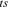
* cells can progress through mitosis due to increased Cdc14 activity mediated by Hog1PP [30]. But we find, in accordance with experiments by Grandin *et al.* [41], that Cdc14 is not the main responsible component for that division. Our model indicates that the stabilisation of Sic1 by Hog1PP is the key mechanism that transfers *cdc15
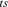
* cells under osmotic stress to the next cell cycle.

Therefore, stabilisation of Sic1 by Hog1PP across all cell cycle phases, seems to be the most important biochemical event in the interaction between osmotic stress and cell cycle progression.

### The Relevance of the Model Predictions for Other Eukaryotes

Our mathematical model is built based on the molecular mechanisms of the model organism *S. cerevisiae*. However, the molecular basis of control of two crucial events of the cell cycle, DNA replication and segregation, is highly conserved in higher eukaryotes, including humans, with CDK playing a universal role [6]. On the other hand, the entire osmotic response pathway is conserved for different fungi, and the Hog1 MAPK cascade is conserved even in higher eukaryotes, also humans [46]. As discussed, the activity of the Hog1 MAPK network affects the activity of CDK by (i) altering the transcription of the cyclin partners of CDK, (ii) prolonging the phosphorylation of CDK and (iii) accumulating the cyclin kinase inhibitor. Hence, the predictions of our model are expected to be relevant for higher eukaryotes. Indeed, one of our model’s predictions, namely accelerated exit from mitosis upon osmotic stress, has been recently validated in *Arabidopsis thaliana* [44].

Note that this model has been developed for doses of osmotic stress between 0.4 M and 1 M NaCl. For higher doses of stress, other links and components, not identified yet, may be involved in cellular response to osmotic stress. Moreover, the repair mechanisms for DNA replication errors are not included in the model. Therefore this model cannot predict the recovery process for the cells that have two sets of DNA because of osmotic stress. Also note that our model is built to unveil the structure of the regulatory mechanisms of the cell in response to osmotic stress rather than to make an exact quantitative prediction of the levels of the proteins involved. In order to achieve that, further experiments and data fitting are necessary.

In summary, our model provides a series of novel predictions for the interactions between the cell cycle and the osmotic stress response, which on one hand are validated by existing experimental data, and on the other hand, suggest new experiments.

## Materials and Methods

### Mathematical Modelling and Simulation

The construction of our model started from two basic modules: the cell cycle module and the osmotic stress response module [33], [47]. We then extracted the molecular mechanisms which affect the cell cycle in the presence of osmotic stress from the literature [1]–[4], [15]. Based on this information we constructed the wiring diagram depicted in Figure 1. New cell cycle elements were added to the cell cycle module model, namely Swe1, Hsl1, Hsl7, Mih1 and their complexes. These are the cell cycle regulated components that are key in the interplay between cell cycle progression and osmotic stress response. These new components were integrated such that the new model of the cell cycle reproduces the phenotypical behaviour of the untreated wild-type and mutated cell [18], [20], [24]–[27], [48]–[50]. Then, the influence of Hog1PP activity on the regulation of the targeted cell cycle components was modelled based on the experiments reported in [1]–[4], [15].

In general the time profile of the concentration 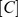 of each component depends on the sum of its production/activation rates 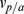, and the sum of its degradation/inhibition rates 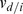:
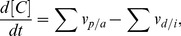
(2)where 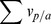 and 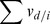 depend on the kinetics of the corresponding interactions.

We used mass-action kinetics, Michaelis-Menten kinetics, and Hill functions to describe the production/activation and degradation/inhibition interactions of each component 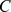. For components that are either active or inactive during the cell cycle, like the transcription factors SBF, MBF and Mcm1, we used the Goldbeter-Koshland switch-like function [51]. Note that there is no unique way to translate the wiring diagram into equations, and the final model depends on the level of required detail [33]. For further details and the complete list of equations, see Supporting Information S1. The set of equations was numerically solved by a 4
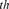
order Runge-Kutta algorithm in MATLAB (MathWorks).

### Parameter Estimation

The set of initial conditions and parameters used in our simulation are presented in Tables S2 and S3 in Supporting Information S1. Parameters were taken from the literature, when available [2], [23], [31], [33], [47], [52]–[57]. The remaining parameters were determined by comparing the simulated dynamical behaviour of the mathematical model with the behaviour of cells in different experimental conditions, as explained below.

The parameters used in the modelling of the morphogenesis checkpoint (see Figure S3 and sections 2.1, 2.2, 2.3 and 2.4 of Supporting Information S1 for details of this model) were adapted from Ciliberto *et al.* [23]. The parameters involved in the interaction of Hog1PP and the cell cycle components were chosen based on the known delay duration of wild-type cells and different mutants under different doses of osmotic stress: the simulation results for the 0.4 M NaCl dose reproduce the delay duration for wild-type and several mutated cells as reported by Escote *et al.*, Clotet *et al.* and Yaakov *et al.* [2]–[4]. Red triangles in Figure 2 shows the delay duration according to our model for wild-type cell. Moreover, the predicted delay for the *sic*1Δ cells is comparable with the known delay [2], [4] (see Figure S4 in Supporting Information S1).

The regulation of Cdc28-Cln2 and Sic1 upon activation of Hog1PP during G1 is shown in Figures S5B–C in Supporting Information S1. Our model also successfully predicts the dynamical behaviour of the G1 phase model published by Adrover *et al.* [5] (see Figures S5A–C, S6A–C in Supporting Information S1). But in contrast to the model of Adrover *et al.* [5], our model is able to predict the activity profile of the Hog1PP targets also when the different doses of stress are applied during the G2-to-M and also M-to-G1 transition (see Figures S5D–I, S6D–I in Supporting Information S1).

The resulting model encompasses a vast amount of known biological experimental knowledge; it adds a substantial amount of information by making biological implicit assumptions mathematically explicit.

### Mathematical Definition of the Cell Cycle Phases

The precise experimental determination of the limits between different cell cycle phases is not straightforward; often cell cycle phases are determined in single cells by monitoring bud formation via microscopy, or DNA content in fluorescence-activated cell sorting (FACS) experiments in the case of a synchronised cell population. The cell cycle, however, is a continuous rather than a discrete progression of biochemical events. When referring to the model predictions though, it is useful to have a precise definition of the borders between the phases. Therefore, here we introduce a mathematical definition of the limits between the cell cycle phases, which we use throughout the paper: the G1 phase starts right after cell division, and finishes when the level of Cdc28-Clb5 crosses the level of Sic1 [21], [58]–[61]. This indicates initiation of DNA replication and therefore the start of the S phase. The S phase finishes when the level of Cdc28-Clb2 becomes greater than the level of Swe1 [50], also defining the start of the G2/M phase (see Figure S9 in Supporting Information S1). The end of the G2/M phase is defined as the point at which Cdc28-Clb2 becomes less than Sic1, which indicates cell division [33]. A further key biochemical event is defined by Mcm1 reaching its maximum level, which marks the beginning of the FINISH process [31]. Note that these definitions of the limits between different cell cycle phases use the cell cycle of a non-stressed cell as a reference. Under osmotic stress the biochemical events dictating the transitions between the phases are distorted and therefore the chosen definitions serve just as reference points.

## Supporting Information

Matlab Programme S1 **The Matlab code is written in such a way that the dose of the stress and the time point of application of the stress can be adjusted by the user.**
(ZIP)

Supporting Information S1 (PDF)

## References

[1] Bellí G, Garí E, Aldea M, Herrero E (2001) Osmotic stress causes a G1 cell cycle delay and downregulation of Cln3/Cdc28 activity in Saccharomyces cerevisiae. Molecular Microbiology 39: 1022–35. 11251821 10.1046/j.1365-2958.2001.02297.x

[2] Escoté X, Zapater M, Clotet J, Posas F (2004) Hog1 mediates cell-cycle arrest in G1 phase by the dual targeting of Sic1. Nature Cell Biology 6: 997–1002. 15448699 10.1038/ncb1174

[3] Clotet J, Escoté X, Adrover M, Yaakov G, Garí E, et al (2006) Phosphorylation of Hsl1 by Hog1 leads to a G2 arrest essential for cell survival at high osmolarity. The EMBO Journal 25: 2338–46. 16688223 10.1038/sj.emboj.7601095 PMC1478172

[4] Yaakov G, Duch A, Garcí-Rubio M, Clotet J, Jimenez J, et al (2009) The stress-activated protein kinase Hog1 mediates S phase delay in response to osmostress. Molecular Biology of the Cell 20: 3572–3582. 19477922 10.1091/mbc.E09-02-0129 PMC2719575

[5] Adrover M, Zi Z, Duch A, Schaber J, Gonzalez-Novo A, et al (2011) Time-dependent quantitative multicomponent control of the G1-S network by the stress-activated protein kinase Hog1 upon osmostress. Science Signalling 4: ra63. 10.1126/scisignal.2002204 21954289

[6] Nurse P (1997) Regulation of the eukaryotic cell cycle. European Journal of Cancer Part A 33: 1002–1004. 9376179 10.1016/s0959-8049(97)00091-9

[7] Nigg E (1995) Cyclin-dependent protein kinases: Key regulators of the eukaryotic cell cycle. BioEssays 17: 471–480. 7575488 10.1002/bies.950170603

[8] Mendenhall M, Hodge A (1998) Regulation of Cdc28 cyclin-dependent protein kinase activity during the cell cycle of the yeast Saccharomyces cerevisiae. Microbiology and Molecular Biology Reviews 62: 1191–1243. 9841670 10.1128/mmbr.62.4.1191-1243.1998 PMC98944

[9] Surana U, Robitsch H, Price C, Schuster T, Fitch I, et al (1991) The role of CDC28 and cyclins during mitosis in the budding yeast S. cerevisiae. Cell 65: 145–61. 1849457 10.1016/0092-8674(91)90416-v

[10] Lew D, Reed S (1993) Morphogenesis in the yeast cell cycle: regulation by Cdc28 and cyclins. The Journal of Cell Biology 120: 1305–20. 8449978 10.1083/jcb.120.6.1305 PMC2119756

[11] Tyers M, Tokiwa G, Futcher B (1993) Comparison of the Saccharomyces cerevisiae G1 cyclins: Cln3 may be an upstream activator of Cln1, Cln2 and other cyclins. The EMBO Journal 12: 1955–68. 8387915 10.1002/j.1460-2075.1993.tb05845.x PMC413417

[12] Schwob E, Nasmyth K (1993) CLB5 and CLB6, a new pair of b cyclins involved in DNA replication in Saccharomyces cerevisiae. Genes and Development 7: 1160–1175. 8319908 10.1101/gad.7.7a.1160

[13] Brewster J, De Valoir T, Dwyer N, Winter E, Gustin M (1993) An osmosensing signal transduction pathway in yeast. Science 259: 1760–1763. 7681220 10.1126/science.7681220

[14] Gustin M, Albertyn J, Alexander M, Davenport K (1998) MAP kinase pathways in the yeast Saccharomyces cerevisiae. Microbiology and Molecular Biology Reviews 62: 1264–1300. 9841672 10.1128/mmbr.62.4.1264-1300.1998 PMC98946

[15] Alexander M, Tyers M, Perret M, Craig B, Fang K, et al (2001) Regulation of cell cycle progression by Swe1p and Hog1p following hypertonic stress. Molecular Biology of the Cell 12: 53–62. 11160822 10.1091/mbc.12.1.53 PMC30567

[16] Schwob E, Böhm T, Mendenhall M, Nasmyth K (1994) The B-type cyclin kinase inhibitor p40SIC1 controls the G1 to S transition in S. cerevisiae. Cell 79: 233–44. 7954792 10.1016/0092-8674(94)90193-7

[17] Verma R, Annan R, Huddleston M, Carr S, Reynard G, et al (1997) Phosphorylation of Sic1p by G1 Cdk required for its degradation and entry into S phase. Science 278: 455–460. 9334303 10.1126/science.278.5337.455

[18] Sia R, Herald H, Lew D (1996) Cdc28 tyrosine phosphorylation and the morphogenesis checkpoint in budding yeast. Molecular Biology of the Cell 7: 1657–66. 8930890 10.1091/mbc.7.11.1657 PMC276016

[19] Amon A, Tyers M, Futcher B, Nasmyth K (1993) Mechanisms that help the yeast cell cycle clock tick: G2 cyclins transcriptionally activate G2 cyclins and repress G1 cyclins. Cell 74: 993–1007. 8402888 10.1016/0092-8674(93)90722-3

[20] McMillan J, Longtine M, Sia R, Theesfeld C, Bardes E, et al (1999) The morphogenesis checkpoint in Saccharomyces cerevisiae: cell cycle control of Swe1p degradation by Hsl1p and Hsl7p. Molecular and Cellular Biology 19: 6929–39. 10490630 10.1128/mcb.19.10.6929 PMC84688

[21] Nash P, Tang X, Orlicky S, Chen Q, Gertler FB, et al (2001) Multisite phosphorylation of a CDK inhibitor sets a threshold for the onset of DNA replication. Nature 414: 514–521. 11734846 10.1038/35107009

[22] Stegmeier F, Amon A (2004) Closing mitosis: The functions of the Cdc14 phosphatase and its regulation. Annual Review of Genetics 38: 203–232. 10.1146/annurev.genet.38.072902.093051 15568976

[23] Ciliberto A, Novak B, Tyson J (2003) Mathematical model of the morphogenesis checkpoint in budding yeast. The Journal of Cell Biology 163: 1243–54. 14691135 10.1083/jcb.200306139 PMC2173725

[24] Theesfeld CL, Zyla TR, Bardes EGS, Lew DJ (2003) A monitor for bud emergence in the yeast morphogenesis checkpoint. Molecular Biology of the Cell 14: 3280–3291. 12925763 10.1091/mbc.E03-03-0154 PMC181567

[25] Burton JL, Solomon MJ (2000) Hsl1p, a Swe1p inhibitor, is degraded via the anaphase-promoting complex. Molecular and Cellular Biology 20: 4614–4625. 10848588 10.1128/mcb.20.13.4614-4625.2000 PMC85864

[26] Simpson-Lavy KJ, Sajman J, Zenvirth D, Brandeis M (2009) APC/C Cdh1 specific degradation of Hsl1 and Clb2 is required for proper stress responses of S. cerevisiae. Cell Cycle 8: 3006–3012. 19713762

[27] Booher RN, Deshaies RJ, Kirschner MW (1993) Properties of Saccharomyces cerevisiae wee1 and its differential regulation of p34CDC28 in response to G1 and G2 cyclins. The EMBO Journal 12: 3417–26. 8253069 10.1002/j.1460-2075.1993.tb06016.x PMC413617

[28] Dahmann C, Diéy J, Nasmyth K (1995) S-phase-promoting cyclin-dependent kinases prevent re-replication by inhibiting the transition of replication origins to a pre-replicative state. Current Biology 5: 1257–1269. 8574583 10.1016/s0960-9822(95)00252-1

[29] Nguyen V, Co C, Li J (2001) Cyclin-dependent kinases prevent DNA re-replication through multiple mechanisms. Nature 411: 1068–1073. 11429609 10.1038/35082600

[30] Reiser V, D’Aquino K, Ly-Sha E, Amon A (2006) The stress-activated mitogen-activated protein kinase signalling cascade promotes exit from mitosis. Molecular Biology of the Cell 17: 3136–3146. 16672381 10.1091/mbc.E05-12-1102 PMC1483046

[31] Chen K, Csikasz-Nagy A, Gyorffy B, Val J, Novak B, et al (2000) Kinetic analysis of a molecular model of the budding yeast cell cycle. Molecular Biology of the Cell 11: 369–391. 10637314 10.1091/mbc.11.1.369 PMC14780

[32] Piatti S, Bohm T, Cocker J, Diéy J, Nasmyth K (1996) Activation of S-phase-promoting CDKs in late G1 defines a ‘point of no return’ after which Cdc6 synthesis cannot promote DNA replication in yeast. Genes and Development 10: 1516–1531. 8666235 10.1101/gad.10.12.1516

[33] Chen K, Calzone L, Csikasz-nagy A, Cross F, Novak B, et al (2004) Integrative analysis of cell cycle control in budding yeast. Molecular Biology of the Cell 15: 3841–3862. 15169868 10.1091/mbc.E03-11-0794 PMC491841

[34] Bardin A, Boselli M, Amon A (2003) Mitotic exit regulation through distinct domains within the protein kinase Cdc15. Molecular and Cellular Biology 23: 5018–5030. 12832486 10.1128/MCB.23.14.5018-5030.2003 PMC162228

[35] Jaspersen S, Charles J, Tinker-Kulberg R, Morgan D (1998) A late mitotic regulatory network controlling cyclin destruction in Saccharomyces cerevisiae. Molecular Biology of the Cell 9: 2803–2817. 9763445 10.1091/mbc.9.10.2803 PMC25555

[36] Morgan D (1999) Regulation of the APC and the exit from mitosis. Nature Cell Biology 1: E47–53. 10559897 10.1038/10039

[37] Traverso E, Baskerville C, Liu Y, Shou W, James P, et al (2001) Characterisation of the Net1 cell cycle-dependent regulator of the Cdc14 phosphatase from budding yeast. Journal of Biological Chemistry 276: 21924–21931. 11274204 10.1074/jbc.M011689200

[38] Stegmeier F, Visintin R, Amon A (2002) Separase, polo kinase, the kinetochore protein Slk19, and Spo12 function in a network that controls Cdc14 localisation during early anaphase. Cell 108: 207–220. 11832211 10.1016/s0092-8674(02)00618-9

[39] McCollum D, Gould K (2001) Timing is everything: regulation of mitotic exit and cytokinesis by the MEN and SIN. Trends in Cell Biology 11: 89–95. 11166217 10.1016/s0962-8924(00)01901-2

[40] Jensen S, Geymonat M, Johnston L (2002) Mitotic exit: delaying the end without FEAR. Current Biology 12: R221–223. 11909552 10.1016/s0960-9822(02)00756-x

[41] Grandin N, De Almeida A, Charbonneau M (1998) The Cdc14 phosphatase is functionally associated with the Dbf2 protein kinase in Saccharomyces cerevisiae. Molecular and General Genetics 258: 104–116. 9613578 10.1007/s004380050712

[42] Cross F, Schroeder L, Kruse M, Chen K (2005) Quantitative characterization of a mitotic cyclin threshold regulating exit from mitosis. Molecular Biology of the Cell 16: 2129–2138. 15716353 10.1091/mbc.E04-10-0897 PMC1087223

[43] Chandrasekaran S, Tan T, Hall J, Cook J (2011) Stress-stimulated mitogen-activated protein kinases control the stability and activity of the Cdt1 DNA replication licensing factor. Molecular and Cellular Biology 31: 4405–4416. 21930785 10.1128/MCB.06163-11 PMC3209262

[44] Skirycz A, Claeys H, de Bodt S, Oikawa A, Shinoda S, et al (2011) Pause-and-stop: The effects of osmotic stress on cell proliferation during early leaf development in Arabidopsis and a role for ethylene signalling in cell cycle arrest. Plant Cell 23: 1876–1888. 21558544 10.1105/tpc.111.084160 PMC3123952

[45] Claeys H, Skirycz A, Maleux K, Inze D (2012) DELLA signalling mediates stress-induced cell differentiation in Arabidopsis leaves through modulation of APC/C activity. Plant Physiology 159: 739–747. 22535421 10.1104/pp.112.195032 PMC3375938

[46] Saito H, Posas F (2012) Response to hyperosmotic stress. Genetics 192: 289–318. 23028184 10.1534/genetics.112.140863 PMC3454867

[47] Zi Z, Liebermeister W, Klipp E (2010) A quantitative study of the Hog1 MAPK response to uctuating osmotic stress in Saccharomyces cerevisiae. PLoS ONE 5: e9522. 20209100 10.1371/journal.pone.0009522 PMC2831999

[48] Sia R, Bardes ES, Lew DJ (1998) Control of Swe1p degradation by the morphogenesis checkpoint. The EMBO Journal 17: 6678–88. 9822611 10.1093/emboj/17.22.6678 PMC1171013

[49] Lew DJ (2000) Cell-cycle checkpoints that ensure coordination between nuclear and cytoplasmic events in Saccharomyces cerevisiae. Current Opinion in Genetics & Development 10: 47–53. 10679396 10.1016/s0959-437x(99)00051-9

[50] Lew D (2003) The morphogenesis checkpoint: how yeast cells watch their figures. Current Opinion in Cell Biology 15: 648–653. 14644188 10.1016/j.ceb.2003.09.001

[51] Goldbeter A, Koshland D (1981) An amplified sensitivity arising from covalent modification in biological systems. Proceedings of the National Academy of Sciences 78: 6840–6844. 10.1073/pnas.78.11.6840 PMC349147 6947258

[52] Cross F, Archambault V, Miller M, Klovstad M (2002) Testing a mathematical model of the yeast cell cycle. Molecular Biology of the Cell 13: 52–70. 11809822 10.1091/mbc.01-05-0265 PMC65072

[53] Su T, Follette P, ÓFarrell P (1995) Qualifying for the license to replicate. Cell 81: 825–828. 7781058 10.1016/0092-8674(95)90000-4 PMC2755094

[54] Zachariae W, Nasmyth K (1999) Whose end is destruction: Cell division and the anaphasepromoting complex. Genes and Development 13: 2039–2058. 10465783 10.1101/gad.13.16.2039

[55] Dirick L, Böhm T, Nasmyth K (1995) Roles and regulation of Cln-Cdc28 kinases at the start of the cell cycle of Saccharomyces cerevisiae. The EMBO Journal 14: 4803–13. 7588610 10.1002/j.1460-2075.1995.tb00162.x PMC394578

[56] Epstein C, Cross F (1994) Genes that can bypass the CLN requirement for Saccharomyces cerevisiae cell cycle START. Molecular and Cellular Biology 14: 2041–2047. 8114735 10.1128/mcb.14.3.2041-2047.1994 PMC358564

[57] Ghaemmaghami S, Huh W, Bower K, Howson R, Belle A, et al (2003) Global analysis of protein expression in yeast. Nature 425: 737–741. 14562106 10.1038/nature02046

[58] Barberis M, Klipp E, Vanoni M, Alberghina L (2007) Cell size at S phase initiation: An emergent property of the G1/S network. PLoS Computational Biology 3: 649–666. 10.1371/journal.pcbi.0030064 PMC1851985 17432928

[59] Barberis M, Beck C, Amoussouvi A, Schreiber G, Diener C, et al (2011) A low number of SIC1 mrna molecules ensures a low noise level in cell cycle progression of budding yeast. Molecular BioSystems 7: 2804–2812. 21717009 10.1039/c1mb05073g

[60] Barberis M, Linke C, Adrover M, Gonzalez-Novo A, Lehrach H, et al (2012) Sic1 plays a role in timing and oscillatory behaviour of B-type cyclins. Biotechnology Advances 30: 108–130. 21963604 10.1016/j.biotechadv.2011.09.004

[61] Venta R, Valk E, Koivomagi M, Loog M (2012) Double-negative feedback between S-phase cyclin-CDK and CKI generates abruptness in the G1/S switch. Frontiers in Neurology 3: 459. 10.3389/fphys.2012.00459 PMC3515773 23230424

