# Supplementary material for: From START to FINISH: The Influence of Osmotic Stress on the Cell Cycle
Source: PLoS One. 2013 Jul 10;8(7):e68067. doi: 10.1371/journal.pone.0068067 (PMC3707922; doi:10.1371/journal.pone.0068067)
Supplement: Matlab Programme S1 — The Matlab code is written in such a way that the dose of the stress and the time point of application of the stress can be adjusted by the user. (ZIP) [file pone.0068067.s001.zip › Readme.rtf]

To run the programme, download all files in a folder.Change the Matlab current directory to your folder.In Matlab workspace type cellcycleduration=Main
