# Supplementary material for: From START to FINISH: The Influence of Osmotic Stress on the Cell Cycle
Source: PLoS One. 2013 Jul 10;8(7):e68067. doi: 10.1371/journal.pone.0068067 (PMC3707922; doi:10.1371/journal.pone.0068067)
Supplement: Supporting Information S1 — (PDF) [file pone.0068067.s002.pdf]

Supporting Information for

From START to FINISH: the influence of osmotic stress on the cell cycle

Elahe Radmaneshfar[1,*], Despoina Kaloriti[2], Michael C. Gustin[3], Neil A. R. Gow[2], Alistair J. P. Brown[2], Celso Grebogi[1], M. Carmen Romano[1,2], Marco Thiel[1].

1 Institute for Complex Systems and Mathematical Biology, SUPA, University of Aberdeen, Aberdeen, UK.

2 Institute of Medical Sciences, Foresterhill, University of Aberdeen, Aberdeen, UK.

3 Department of Biochemistry and Cell Biology, Rice University, Houston, TX, USA.

∗ **E-mail: radmaneshfar@gmail.com**

Contents

1 Deriving the wiring diagram **2**
1.1 The cell cycle network . . . 2
1.2 Osmotic stress response . . . 3
1.3 Interaction between cell cycle and osmotic stress networks . . . 3

2 Model description **4**
2.1 Modelling the regulation of Swe1 . . . 5
2.2 Modelling the regulation of Hsl1-Hsl7 complex under osmotic stress . . . 6
2.3 Modelling the regulation of Mih1 . . . 7
2.4 Modelling the regulation of Clb2 . . . 7
2.5 Modelling cell growth . . . 9
2.6 Modelling the influence of Hog1PP on the cyclin transcription . . . 10
2.7 Modelling the regulation of Sic1 under osmotic stress . . . 10

3 Sensitivity analysis of the estimated parameters **12**

4 Equations **14**

5 Initial conditions and parameters **21**

6 Figures **23**

Here we provide the details of our approach to build the mathematical model, which describes the influence of osmotic stress on the cell cycle.

1 Deriving the wiring diagram

Based on the molecular mechanisms which control the cell cycle [1–17], and those which affect the cell cycle in the presence of osmotic stress [18–24], we construct the wiring diagram depicted in Figure 1 of the main text. The resulting network is composed of three major modules: the cell cycle control network, the osmotic stress signalling and the interaction between both. Note that to avoid unnecessary complexity, we just display the downstream component of the osmotic stress signalling, Hog1PP in Figure 1. However, the regulation of Hog1PP has been modelled considering the activity of MAPK signalling pathway. Note that some components, such as Hog1PP and Swe1, appear several times in the diagram. This has been done for clarity purposes, because they are involved in multiple reactions. Next, we explain briefly the interactions included in each module.

1.1 The cell cycle network

The cell cycle is controlled by a complex molecular network, responsible for the self-sustained oscillatory expression of a large subset of genes. The cell cycle of eukaryotes consists of four different phases, denoted by G1 (Gap 1), S (DNA Synthesis), G2 (Gap 2) and M (Mitosis). The cell transition between phases are controlled by the successive activation and inactivation of Cyclin-Dependent Kinases (CDKs) [8]. CDK activity (Cdc28 in *S. cerevisiae*) is regulated by the availability of its cyclin partners, inhibitory tyrosine phosphorylation and binding to stoichiometric CDK inhibitors [8]. Cdc28 has two types of associated cyclins: (i) the three G1 cyclins, (Cln1, Cln2, Cln3) and (ii) the six B-type cyclins (Clb1 to Clb6) [8].

The transition from the G1-to-S phase is called START. START refers to the transcriptional cascade triggering three main events: budding, synthesising and duplicating the spindle pole bodies [4]. START is primarily induced by the Cdc28-Cln3 complex, an upstream activator of the G1 cyclins [2]. Cdc28-Cln3 activates the transcription factor complexes, SBF and MBF (link sr1 in Figure S1). These in turn trigger the transcription of a set of approximately 200 genes [25], most importantly *CLN2* and *CLB5*. Transcription of *CLN2* and *CLB5* makes the proteins available to bind to Cdc28, thereby increasing the levels of Cdc28-Cln2 and Cdc28-Clb5 (links sr2 and sr3 in Figure S1). The positive feedback loop (link sr5 in Figure S1) makes this transition switch-like [4, 26].

Sic1, the Cyclin Kinase Inhibitor (CKI) in the G1 phase, keeps Cdc28-Clb5 inactive during most of START [9]. To enable the G1-to-S transition, Sic1 has to be inactivated. Cdc28 phosphorylates Sic1 (link sr6 in Figure S1) [9] and targets it for degradation by the SCF complex (link sr16 in Figure S1) [9, 10]. The absence of the inhibitor Sic1 triggers the activity of Cdc28-Clb5, which initiates the DNA replication and transfers the cell to the S phase.

DNA replication is a multi-step process which begins at the origins of replication. The multiprotein complex ORC (Origin Recognition Complex) remains bound to the origins of replication throughout the cell cycle. The first step of DNA replication takes place during the late M phase and early G1 phase. In this step, the pre-replicative complex, required for the initiation of DNA replication [27], binds to the ORC. The pre-replicative complex consists of the proteins Cdc6, Cdt1 and six related proteins called Mcm. The second step of DNA replication occurs at the onset of the S phase triggered by the activity of Cdc28-Clb5. During this step the pre-replicative complex is disassembled from the origins of replication, mediated by the activity of Cdc28-Clb5. Therefore, Cdc28-Clb5 has a dual role: on the one hand it enables initiation of DNA replication, and on the other hand, it warrants that DNA replication happens only once before mitosis by disassembling the pre-replicative complex from the origin of replication [28] (the pre-replicative complex is represented by Cdc6 in our model).

The next cell cycle transition, G2-to-M, is mainly governed by the activity of Cdc28-Clb2 [1], which in turn is regulated by several mechanisms. On the one hand, the protein kinase Swe1 inhibits Cdc28-Clb2 activity by tyrosine phosphorylation of Cdc28 (link sr10 in Figure S1) [5]. Swe1 is part of the morphogenesis check-point, and it prevents the cell entering into the G2 phase when aspects of bud formation are defective [5, 7]. Swe1 is quickly degraded, mediated by the Hsl1-Hsl7 complex, as well as Cdc28-Clb2 (links sr11, sr12 and sr22 in Figure S1) . It is worth mentioning that the phosphatase Mih1 reverses the tyrosine phosphorylation of Cdc28 (link sr9 in Figure S1) [5]. On the other hand, the availability of Clb2 is transcriptionally controlled; Cdc28-Clb2 activates Mcm1 (link sr20 in Figure S1) [3], which is a transcription factor of *CLB2*, thereby establishing a positive feedback loop (links sr20 and sr21 in Figure S1). Active Cdc28-Clb2, therefore, transfers the cell to the M phase, during which the replicated chromosomes are segregated and nuclear division takes place.

Exit from mitosis is achieved mainly by the inactivation of Cdc28-Clb2 [24]. This is mediated by the degradation of Clb2, performed by two major APC (Anaphase Promoting Complex) components, Cdc20 and Cdh1 (links sr13 and sr14 in Figure S1) [16, 17]. First, Cdh1 is absent and Clb2 is primarily degraded by a Cdc20 dependent mechanism. Cdc20 also triggers a pathway that results in the activation of Cdh1 and Cdc14 [16, 17, 24]. The remaining fraction of the Clb2 is degraded by Cdh1 in the exit from mitosis [17]. The return to the G1 phase prompted by the phosphatase Cdc14 occurs in multiple steps: (i) Cdc14 dephosphorylates and activates Cdh1 (link sr24 in Figure S1); (ii) Cdc14 activates Swi5 (link sr25 in Figure S1), which is a transcription factor of Sic1 and Cdc6 (Sic1 and Cdc6 are shown by CKIs in Figure S1) [6], and (iii) Cdc14 also dephosphorylates and activates Sic1 and Cdc6 (link sr26 in Figure S1). Therefore, the activity of CKIs (Sic1 and Cdc6) and Cdh1 ensures Cdc28-Clb2 inactivation (see Figure S1).

1.2 Osmotic stress response

Osmoregulation is a homeostatic process, highly conserved across species, which regulates internal turgor pressure, as well as the water content and the volume of the cell. Osmotic stress is sensed by various receptors and causes the activation of different signalling pathways [29]. The High-Osmolarity Glycerol (HOG) MAPK signalling plays a key role in osmoregulation [30,31]. The HOG MAP kinase pathway, like any other MAP kinase pathway, has three categories of protein kinases: a MAP kinase, a MAP kinase kinase (MAPKK), and a MAP kinase kinase kinase (MAPKKK) [29,32]. For *S. cerevisiae*, Sln1, Msb2, Hkr1 and Sho1 [33–36] have been reported as osmosensors of three upstream branches which monitor changes in the turgor pressure and independently regulate three MAPKKKs (Ste11, Ssk2 and Ssk22) [29, 37]. Consequently, MAPKKKs phosphorylate and activate the MAPKK (Pbs2) [29]. Activation of Pbs2 results in the activation of Hog1 via phosphorylation [29]. Dually phosphorylated Hog1 (Hog1PP) then accumulates in the nucleus and activates gene expression of proteins involved in glycerol production (see Figure S2). Thereby, glycerol production increases to compensate the turgor pressure loss [32].

As we will see in the next section, phosphorylated Hog1, a result of osmotic stress, is the main player in the interactions between the osmotic stress response and cell cycle networks.

1.3 Interaction between cell cycle and osmotic stress networks

The kinase Hog1PP interacts with different cell cycle regulated proteins [18–22]. The phase of the cell cycle during which the osmotic stress is applied dictates the mechanisms of interaction of Hog1PP with the cell cycle machinery.

The G1-to-S transition is halted upon activation of Hog1PP. Activity of Hog1PP blocks this transition by a dual mechanism [18, 20]: (i) Hog1PP downregulates the transcription of the G1 cyclins [18, 20] and (ii) Hog1PP phosphorylates Sic1 directly on a specific site [20]. This reduces the binding of Sic1 to the Cdc4 complex [20]. This complex formation is required to initiate the ubiquitin-mediated proteolysis pathway responsible for Sic1 degradation [38]. Therefore, the presence of Hog1PP stabilised the level of

Sic1 [20]. Hence, G1 arrest as response to osmotic stress is the consequence of Sic1 accumulation, which is caused by direct phosphorylation of Sic1 and downregulation of the G1 cyclins by Hog1PP [20].

Application of osmotic stress after the transition to the S phase delays the cell cycle due to three main reported mechanisms: (i) direct downregulation of *CLB5* transcription by Hog1PP [22], (ii) accumulation of the protein kinase Swe1, and (iii) downregulation of the M phase cyclin *CLB2*. The protein kinase Swe1 is cell cycle regulated [7,11]. Swe1 synthesis is mediated by the transcription factor SBF during late G1 phase and early S phase [5]. Rapid degradation of Swe1 is regulated by sequential phosphorylations, which are caused by the activity of the Hsl1-Hsl7 complex, as well as by the activity of Cdc28-Clb2 [5,7]. However, in the presence of osmotic stress, Hog1PP phosphorylates Hsl1. Phosphorylated Hsl1 then cannot bind to Hsl7 and hence the Hsl1-Hsl7 complex is not formed. As a consequence, Swe1 is not degraded [21]. Hence, osmotic stress applied at the onset of the G2 phase inhibits Cdc28-Clb2 activity due to two mechanisms: (i) stabilisation of Swe1 [21], and (ii) direct downregulation of *CLB2* [19,21]. Inactivation of Cdc28-Clb2 blocks the G2-to-M transition.

These are the reported interactions between the osmotic stress response and the cell cycle networks. Despite the existence of all these experimental data, some questions still remain open. For example, there is experimental evidence that Hog1PP phosphorylates Sic1 [20], but it is unknown whether Hog1PP phosphorylates Sic1 when the latter is in a complex with Cdc28-Clb5 or with Cdc28-Clb2. Hence, we made a series of assumptions, which are stated in the main text section and summarised in Figure 1 of the main text (indicated by orange).

2 Model description

The dynamics of the concentration $[C]$ of each component is described by an ordinary differential equation in which the rate of change of $[C]$ depends on the sum of its production/activation rates $v_{p/a}$, and the sum of its degradation/inhibition rates $v_{d/i}$:

$$\frac{d[C]}{dt} = \sum v_{p/a} - \sum v_{d/i}, \tag{1}$$

where $\sum v_{p/a}$ and $\sum v_{d/i}$ depend on the kinetics of the corresponding interactions.

The molecular interactions are modelled by mass-action kinetics, Michaelis-Menten kinetics, and Hill functions. For components which are either active or inactive during the cell cycle, such as transcription factors SBF, MBF and Mcm1, the Goldbeter-Koshland switch-like function is used [39].

As mentioned in Materials and Methods, our model starts from two basic modules: the cell cycle module and the osmotic stress response module [40,41]. First, in order to model the molecular interactions between the osmotic stress response and the cell cycle network components, we have to introduce the dynamics of a substantial number of new cell cycle components and their complexes not considered before [40]. Then the dynamics of these new components has to be incorporated into the cell cycle network model such that the new model of the cell cycle reproduces the phenotypical behaviour of the wild-type and mutated cell [5,7,11–15,23,42]. Finally, we take into account the interactions of Hog1PP with the cell cycle regulated components such that the model mimics the experimental observations of the reaction of wild-type and mutated cell to osmotic stress [18–22].

In the following subsections we present in detail the substantial extensions of the cell cycle module and the modelling of the key interactions between the cell cycle and the osmotic stress pathway which make possible the coupling between both modules. We start with the modelling of the morphogenesis checkpoint.

Modelling the morphogenesis checkpoint

The morphogenesis checkpoint refers to the control mechanisms that hinder the progression through the G2 phase in unfavourable conditions. These mechanisms are achieved through the control of Cdc28-Clb2 activity [5, 7]. This checkpoint is inactive under optimal growth conditions, but it becomes active under a malformation of bud or osmotic stress, halting cell cycle progression.

Swe1 inhibits the activity of Cdc28-Clb2 during the late G1 and S phase. In turn, the activity of Swe1 is inhibited via the Hsl1-Hsl7 complex and Cdc28-Clb2 [5, 7] (for details of Cdc28-Clb2 regulation and Swe1 regulation see Sections 1.1 and 2.1 of Supplementary Information).

We therefore developed a model for the morphogenesis checkpoint which includes the dynamics of Hsl1-Hsl7 regulation explicitly, based on the recently unveiled molecular mechanisms of the morphogenesis checkpoint [12–15]. This is in contrast to the model of Ciliberto *et al.*, where the dynamics of Hsl1-Hsl7 was not considered, and instead, reduced to a fixed parameter [43]. We first assumed that Swe1 can exist in four different forms, namely, unphosphorylated (denoted by Swe1), phosphorylated via Hsl1-Hsl7 (denoted by Swe1M), phosphorylated via Cdc28-Clb2 (denoted by Swe1P), and doubly phosphorylated (denoted by Swe1MP), in accordance with biological studies [5, 7, 11–13, 23, 42] (see Figure S3). Note that the last form is expected to be highly unstable [44]. We furthermore included the interactions of all forms of Swe1 with Mih1, Hsl1, Hsl7, Cdc28-Clb2 and, additionally, we incorporated the interactions that link the morphogenesis checkpoint to the osmotic stress response module. Note that the mechanisms responsible for the morphogenesis checkpoint alone are highly complex and, in our model, we do not include components such as Gin4, Elm1, Cla4, which have been implicated in the morphogenesis checkpoint, but do not interact with Hog1PP. As such, we keep the model as simple as possible, while preserving the main biological mechanisms. We then integrated this model into the cell cycle module by considering the interaction of the morphogenesis checkpoint elements with the rest of the cell cycle components. We validated this new cell cycle model by reproducing the phenotypical observation of the cell cycle of the wild-type and various mutated cells [5, 7, 11–13, 23, 42].

2.1 Modelling the regulation of Swe1

Swe1 is not a crucial component for cells growing in optimal conditions, since *swe1*Δ cells exhibit a normal cell cycle in controlled environments [23]. However, Swe1 becomes pivotal in cells exposed to osmotic stress. We assumed that Swe1 can be present in four different forms during the cell cycle, in accordance with biological studies [5, 7, 11–13, 23, 42]. Activity of Swe1, is regulated by several mechanisms:

(i) Synthesis of Swe1 takes place via transcription factor SBF [5]. Hence, in wild type cells, accumulation of Swe1 begins in late G1 and peaks in S phase or early G2 phase. The production of Swe1 is described by:

$$v_{pSwe1} = k_{sswe}[SBF] + k_{ssweC}, \tag{2}$$

where k_{sswe} represents the synthesis rate of Swe1 by SBF and k_{ssweC} denotes the basal production rate of Swe1.

(ii) Swe1 is tagged for rapid degradation during G2 and M phase via a pathway which contains the Hsl1-Hsl7 complex and Cdc28-Clb2 [7,11,12,44]. When the Hsl1-Hsl7 complex is present, the kinase Swe1, which has been accumulated in the nucleus until then, is moved to the bud neck [12, 13, 44]. We denote the product of post-translational modification of Swe1 by Hsl1-Hsl7 by Swe1M, Eq.(6). We modelled the inhibition of Swe1 by the Hsl1-Hsl7 complex with a kinetic law similar to a Hill function:

$$v_{pSwe1M} = \frac{k_{hsl1}[Hsl1Hsl7][Swe1]}{J_{iwee} + [Swe1]}, \tag{3}$$

where k_{hsl1} represents the rate of formation of Swe1M and J_{iwee} is the inverse of the inflection point. The formation of the Hsl1-Hsl7 complex is explained in Section 2.2.

(iii) Also, Cdc28-Clb2 phosphorylates Swe1 [44]. We denote this intermediate product by Swe1P, Eq.(7), which is targeted for degradation [7]. We modelled the phosphorylation of Swe1 by Cdc28-Clb2 also by a Hill function since they are the antagonistic:

$$v_{pSwe1P} = \frac{V_{iwee}[Clb2][Swe1]}{J_{iwee} + [Swe1]}. \tag{4}$$

Therefore, the dynamics of Swe1 was modelled by the following equation:

$$\begin{aligned} \frac{d[Swe1]}{dt} &= v_{pSwe1} - v_{pSwe1M} + v_{rSwe1M} - v_{pSwe1P} + v_{rSwe1P} - v_{dSwe1} \\ &= k_{sswe}[SBF] + k_{ssweC} - \frac{k_{hsl1}[Hsl1Hsl7][Swe1]}{J_{iwee} + [Swe1]} + k_{hsl1r}[Swe1M] \\ &\quad - \frac{V_{iwee}[Clb2][Swe1]}{J_{iwee} + [Swe1]} + \frac{V_{awee}[Swe1P]}{J_{awee} + [Swe1P]} - kk_{dswe}[Swe1], \end{aligned} \tag{5}$$

where v_{rSwe1M} describes the backward reaction from Swe1M to Swe1, v_{rSwe1P} explains the reverse reaction from Swe1P to Swe1 and v_{dSwe1} represents the natural degradation of kinase Swe1.

Using similar arguments, we derived the following mathematical equations describing the dynamics of Swe1M, Swe1P and Swe1MP (see Figure S3):

$$\begin{aligned} \frac{d[Swe1M]}{dt} &= \frac{k_{hsl1}[Hsl1Hsl7][Swe1]}{J_{iwee} + [Swe1]} - k_{hsl1r}[Swe1M] - \frac{V_{iwee}[Clb2][Swe1M]}{J_{iwee} + [Swe1M]} \\ &\quad + \frac{V_{awee}[Swe1MP]}{J_{awee} + [Swe1MP]} - kk_{dswe}[Swe1M]. \end{aligned} \tag{6}$$

$$\begin{aligned} \frac{d[Swe1P]}{dt} &= -\frac{k_{hsl1}[Hsl1Hsl7][Swe1P]}{J_{iwee} + [Swe1P]} + k_{hsl1r}[Swe1MP] - \frac{V_{awee}[Swe1P]}{J_{awee} + [Swe1P]} \\ &\quad - kk_{dswe}[Swe1P] + \frac{V_{iwee}[Clb2][Swe1]}{J_{iwee} + [Swe1]}. \end{aligned} \tag{7}$$

$$\begin{aligned} \frac{d[Swe1MP]}{dt} &= \frac{k_{hsl1}[Hsl1Hsl7][Swe1P]}{J_{iwee} + [Swe1P]} - k_{hsl1r}[Swe1MP] \\ &\quad - \frac{V_{awee}[Swe1MP]}{J_{awee} + [Swe1MP]} + \frac{V_{iwee}[Clb2][Swe1M]}{J_{iwee} + [Swe1M]} - kk_{dswe}[Swe1MP]. \end{aligned} \tag{8}$$

The phosphorylation of Swe1 by Cdc28-Clb2 decreases the activity of the kinase Swe1. Both Swe1M and Swe1P are stable, but the product Swe1MP is highly unstable [43]. Swe1MP is a post-translational modification of Swe1 for which Hsl1-Hsl7 and Cdc28-Clb2 are needed. Hence, in accordance with the reported experiments we assumed that for degradation of Swe1, both Hsl1-Hsl7 and Cdc28-Clb2 are required [7, 11, 44, 45].

2.2 Modelling the regulation of Hsl1-Hsl7 complex under osmotic stress

Formation of the Hsl1-Hsl7 complex is necessary for Swe1 degradation [45]. Therefore we included it in our model. This complex is located on the bud neck [45], but spatial modelling of Hsl1 and Hsl7 localisation on the bud neck is beyond the scope of this paper. Instead, we built a temporal model for the regulation of this complex. Since Hsl1 is the dominant component in the formation of the Hsl1-Hsl7 complex [13], we can abstract the dynamics of the formation of the Hsl1-Hsl7 complex by Hsl1. Hsl1 activity correlates with bud emergence and remains stable up to nuclear division [13]. The localisation of

Hsl1 and Hsl7 to the septin cortex takes place exactly after bud formation [13]. This supports our choice of Hsl1 as a representative for the Hsl1-Hsl7 complex.

In the presence of osmotic stress, Hog1PP interacts with Hsl1 and hinders the formation of the Hsl1-Hsl7 complex. As a consequence, Swe1 is not degraded and Hsl7 is delocalised from the bud neck [21]. Therefore, the formation of the Hsl1-Hsl7 complex was modelled by:

$$v_{pHsl1Hsl7} = \frac{kk_{Hsl1Hsl7}[BUD]}{1 + \left(\frac{[Hog1PP]}{J_{Hsl1}}\right)^{n_{Hsl1d}}}, \tag{9}$$

where BUD denotes a mathematical function which describes the observation of bud formation and depends on Cln2, Cln3 and Clb5 (Eq.(70) of Section 4) [40].

Moreover, Hsl1 is stable until nuclear division. As well as Clb2, Hsl1 is a substrate of APC [14, 15]. Therefore, following this experimental evidence [14, 15] we assumed that its degradation follows a similar APC dependent mechanism to the one of Clb2. Moreover, we assumed that it also has a natural self-degradation. Hence, the degradation terms of Hsl1-Hsl7 are given by:

$$v_{dHsl1Hsl7} = kkk_{Hsl1Hsl7}V_{db2}[Hsl1Hsl7] + kkd_{Hsl1Hsl7}[Hsl1Hsl7], \tag{10}$$

where $V_{db2} = kk_{db2} + kkk_{db2}[Cdh1] + k_{db2p}[Cdc20_A]$ represents the APC dependent degradation of the Hsl1-Hsl7 complex and the parameter $kkd_{Hsl1Hsl7}$ captures the rate of self-degradation.

As a result, considering Eqs.(9) and (10), the regulation of the Hsl1-Hsl7 complex is described by:

$$\begin{aligned} \frac{d[Hsl1Hsl7]}{dt} &= v_{pHsl1Hsl7} - v_{dHsl1Hsl7} \qquad (11) \\ &= \frac{kk_{Hsl1Hsl7}[BUD]}{1 + \left(\frac{[Hog1PP]}{J_{Hsl1}}\right)^{n_{Hsl1d}}} - kkk_{Hsl1Hsl7}V_{db2}[Hsl1Hsl7] - kkd_{Hsl1Hsl7}[Hsl1Hsl7]. \end{aligned}$$

2.3 Modelling the regulation of Mih1

The protein phosphatase Mih1 reverses the phosphorylation of Cdc28-Clb2 which is caused by activity of Swe1 (see link sr9 in Figure S1) [5]. We assumed that there is a positive feedback between Cdc28-Clb2 and Mih1 (Cdc28-Clb2 activates Mih1) [43]. Since details of Mih1 regulations are still unknown, we used the following Hill functions to describe its activation by Clb2 and its self-inhibition [43]

$$\frac{d[Mih1]}{dt} = \frac{Va_{mih}[Clb2]\left([Mih1_T] - [Mih1]\right)}{Ja_{mih} + [Mih1_T] - [Mih1]} - \frac{Vi_{mih}[Mih1]}{Ji_{mih} + [Mih1]}, \tag{12}$$

where $[Mih1_T]$ is the total concentration of Mih1 in the cell, which is assumed to be constant. Note that $[Mih1_T]$ is defined as $[Mih1_T] = [Mih1_i] + [Mih1]$, therefore instead of $[Mih1_i]$ (inactive form of Mih1) we use $[Mih1_T]$ in the model.

2.4 Modelling the regulation of Clb2

The pair of mitotic cyclins Clb1 and Clb2 are represented by Clb2 in our model. Clb2 is crucial for successful mitosis and its mutation causes G2 arrest [1]. The activity of Cdc28-Clb2 is regulated by several mechanisms. The activity of Hog1PP blocks the G2-to-M transition by influencing the activity of Cdc28-Clb2. The protein kinase Swe1, accumulated upon activation of Hog1PP, inhibits Cdc28-Clb2 by tyrosine phosphorylation of Cdc28 [21]. Also, Hog1PP downregulates the transcriptional activity of *CLB2* [19, 21]. Hence, we need to take the interaction of Clb2 with Swe1 and also Hog1PP into consideration:

(i) The availability of Clb2 is transcriptionally controlled. Cdc28-Clb2 activates Mcm1, which is the transcription factor of *CLB2*, thereby establishing a positive feedback loop [3]. Also the presence of an osmotic stress influences the transcription of the M phase cyclin [19, 21]. Hence, the transcriptional production of Clb2 is described by:

$$v_{pClb2} = \frac{(kk_{sb2} + kkk_{sb2}[Mcm1])\,[mass]}{1 + (k_{dHog1Clb2}[Hog1PP])^{n_{Hog1Clb2}}}, \tag{13}$$

where the parameter kk_{sb2} captures the basal transcription of *CLB2*, and kkk_{sb2} describes the induced expression of *CLB2* by Mcm1. Activity of Hog1PP also changes the transcription of *CLB2* (see Section 2.6). Note that we use Goldbeter-Koshland function to model the Mcm1 activity, Eq.(114) of Section 4.

(ii) The stoichiometric inhibitors Sic1, Sic1h (Sic1 which is phosphorylated by Hog1PP) and Cdc6, when active, bind to Clb2, thereby inhibiting Clb2 activity. We described these tethering by the following equations:

$$\begin{aligned} v_{pC2} &= k_{asb2}[Sic1][Clb2], \\ v_{pF2} &= k_{asf2}[Cdc6][Clb2], \\ v_{pC2h} &= k_{hasb2}[Sic1h][Clb2]. \end{aligned} \tag{14}$$

Additionally, when Sic1, Sic1h or Cdc6 is bound to Clb2, the former can degraded, thereby releasing Clb2. Note that the degradation rate of Sic1h is reduced compared with Sic1 [20]. We described these releasing by the following equations:

$$\begin{aligned} v_{dC2} &= k_{dib2}[C2], \\ v_{dC2P} &= k_{d3c1}[C2P], \\ v_{dF2} &= k_{dif2}[F2], \\ v_{dF2P} &= k_{d3f6}[F2P], \\ v_{dC2h} &= k_{hdib2}[C2h], \\ v_{dC2hP} &= k_{hd3c1}[C2hP], \end{aligned} \tag{15}$$

where v_{dC2} and v_{dC2P} represent the release of Cdc28-Clb2 from the Cdc28-Clb2-Sic1 complex (denoted by C2) and the Cdc28-Clb2-Sic1P complex (C2P), respectively. Likewise, v_{dF2} and v_{dF2P} describe the unbinding of Cdc28-Clb2 from the Cdc28-Clb2-Cdc6 complex (F2), and from the Cdc28-Clb2-Cdc6P complex (F2P), respectively. Finally, v_{dC2h} and v_{dC2hP} illustrate the dissociation of Cdc28-Clb2 from the Cdc28-Clb2-Sic1h complex (C2h) and from the Cdc28-Clb2-Sic1hP complex (C2hP), respectively. Note that we distinguish between Sic1, which is phosphorylated by Cdc28 (Sic1P) and the one which is phosphorylated by Hog1PP (Sic1h) (see section 2.7).

(iii) Swe1 inhibits Cdc28-Clb2 activity by tyrosine phosphorylation of Cdc28 [5, 23]. We assume that all three stable forms of the kinase Swe1 can inactivate Cdc28-Clb2 [7, 11, 12, 44]. This phosphorylation is described by:

$$v_{iSwe1Clb2} = K_{Swe1}[Clb2], \tag{16}$$

where $K_{Swe1} = kk_{swe}[Swe1] + kkk_{swe}[Swe1M] + kkkk_{swe}[Swe1P]$.

(iv) Entry into mitosis requires Cdc28-Clb2 to be active. Mih1 reverses the phosphorylation of Cdc28 which is caused by Swe1 [5]:

$$v_{aMih1Clb2} = K_{Mih1}[PClb2], \tag{17}$$

where $K_{Mih1} = kk_{mih1}[Mih1] + kkk_{mih1}([Mih1_T] - [Mih1])$ and PClb2 represents the complex Cdc28-Clb2 in which Cdc28 is phosphorylated by Swe1. The regulation of PClb2 is modelled by Eq.(76) of Section 4.

Table S 1. Table of abbreviation for different complexes of Cdc28-Clb2

abbreviation	complex
C2	Cdc28-Clb2-Sic1
C5	Cdc28-Clb5-Sic1
F2	Cdc28-Clb2-Cdc6
C2P	Cdc28-Clb2-Sic1P (C2 phosphorylated by Cdc28)
F2P	Cdc28-Clb2-Cdc6P (F2 phosphorylated by Cdc28)
C2h	Cdc28-Clb2-Sic1h (C2 phosphorylated by Hog1PP)
C2hP	Cdc28-Clb2-Sic1hP (C2h phosphorylated by Cdc28)
PF2	Cdc28P-Clb2-Cdc6 (F2 phosphorylated by Swe1)
PF2P	Cdc28P-Clb2-Cdc6P (PF2 phosphorylated by Cdc28)
PTrim	Cdc28P-Clb2-Sic1 (C2 phosphorylated by Swe1)
PTrimP	Cdc28P-Clb2-Sic1P (PTrim phosphorylated by Cdc28)
PTrimh	Cdc28P-Clb2-Sic1h (PTrim phosphorylated by Hog1PP)
PTrimhP	Cdc28P-Clb2-Sic1hP (PTrimh phosphorylated by Cdc28)
C5P	Cdc28-Clb5-Sic1P (C5 phosphorylated by Cdc28)
C5h	Cdc28-Clb5-Sic1h (C5 phosphorylated by Hog1PP)
C5hP	Cdc28-Clb5-Sic1hP (C5h phosphorylated by Cdc28)

(v) Degradation of Clb2 is performed by two major APC subunits: Cdc20 and Cdh1 [16,17]. During anaphase, Cdh1 is absent and Clb2 is primarily degraded by Cdc20. Cdc20 also triggers the pathway that results in the activation of Cdh1 [16,17]. The remaining fraction of the Clb2 is degraded by Cdh1 during exit from mitosis [17]. The equation for this regulation is:

$$v_{dClb2} = V_{db2}[Clb2], \tag{18}$$

where $V_{db2} = kk_{db2} + kkk_{db2}[Cdh1] + k_{db2p}[Cdc20_A]$ represents the APC dependent degradation of Clb2.

Hence, the regulation of Cdc28-Clb2 can be summarised by the following equation:

$$\begin{aligned}
\frac{d[Clb2]}{dt} &= v_{pClb2} + v_{dC2} + v_{dC2P} + v_{dF2} + v_{dF2P} + v_{dC2h} + v_{dC2hP} \\
&\quad -v_{pC2} - v_{pF2} - v_{pC2h} + v_{iMih1Clb2} - v_{iSwe1Clb2} - v_{dClb2} \\
&= \frac{(kk_{sb2} + kkk_{sb2}[Mcm1])\,[mass]}{1 + (k_{dHog1Clb2}[Hog1PP])^{n_{Hog1Clb2}}} \\
&\quad +k_{dib2}[C2] + k_{d3c1}[C2P] + k_{hdib2}[C2h] + k_{hd3c1}[C2hP) + k_{dif2}[F2] + k_{d3f6}[F2P] \\
&\quad - (V_{db2} + k_{asb2}[Sic1] + k_{asf2}[Cdc6] + k_{hasb2}[Sic1h])\,[Clb2] \\
&\quad + (K_{Mih1}[PClb2] - K_{Swe1}[Clb2])\,.
\end{aligned} \tag{19}$$

Moreover, biological evidence suggests that Swe1 can also phosphorylate Cdc28 in the complexes of C2, C2P, C2h and C2hP (see Table S1 for abbreviation) [5, 7, 11, 12, 23, 44]. We denote the complexes Sic1-Cdc28P-Clb2, Sic1P-Cdc28P-Clb2, Sic1h-Cdc28P-Clb2 and Sic1Ph-Cdc28P-Clb2 by PTrim, PTrimP, PTrimh and PTrimhP, respectively (see Table 1 for abbreviation). Figure 1 of main text shows how these complexes relate to C2, C2P, C2h and C2hP. The regulation of PTrim, PTrimP, PTrimh and PTrimhP were modelled by Eq.(77), Eq.(78), Eq.(84) and Eq.(85) of Section 4, respectively. Likewise, we introduced the Cdc6-Cdc28P-Clb2 and Cdc6P-Cdc28P-Clb2 complexes when Swe1 phosphorylates Cdc28 in the F2 and F2P complexes. The Cdc6-Cdc28P-Clb2 and Cdc6P-Cdc28P-Clb2 are denoted by PF2 and PF2P, respectively. Eq.(79) and Eq.(80) of Section 4 model their regulations.

2.5 Modelling cell growth

Since osmotic stress arrests cells at different stages, a pure exponential model for the mass would lead to cells reaching unrealistic sizes. We therefore modelled cell growth such that cell growth is limited. Cell

size is proportional to its mass [40,46] and also upon osmotic stress, cell volume decreases [47]. Hence, cell growth under osmotic stress is slower compared with an untreated cell. Hence, we describe cell growth under osmotic stress as:

$$\frac{d[mass]}{dt} = \frac{k_g[mass]\,(M_{mass,max} - [mass])}{1 + k_{dHog1mass}[Hog1PP]}, \tag{20}$$

where k_g is the growth rate in untreated conditions, $M_{mass,max}$ is the maximum reported mass for the cell, and $k_{dHog1mass}$ represents the influence of Hog1PP on the cell growth.

2.6 Modelling the influence of Hog1PP on the cyclin transcription

The cyclins Cln1 and Cln2 are lumped together as Cln2, the S phase cyclins Clb5 and Clb6 are represented by Clb5, and the mitotic cyclins Clb1 and Clb2 are represented by Clb2. Moreover, in our model the synthesis rate of each cyclin (Cln2, Clb5, Clb2 and Cln3) is a linear function of mass (Eq.(35), Eq.(36), Eq.(37) and Eq.(88) of Section 4). These equations describe cyclin concentrations inside the nucleus. Since, Hog1PP is located in the nucleus [48], our model for cyclin regulation inside the nucleus can describe their changes upon osmotic stress. Doubly phosphorylated Hog1 strongly changes the transcription rate of cyclins [18, 20–22]. We modelled the Hog1PP effect on the transcription rates by Hill functions (Eq.(35), Eq.(36), Eq.(37) and Eq.(88) of Section 4).

2.7 Modelling the regulation of Sic1 under osmotic stress

The activity of Sic1 in the G1 phase controls the G1-to-S transition [9]. Several mechanisms are involved in the regulation of Sic1:

(i) The availability of Sic1 is transcriptionally controlled. The transcription factor Swi5 activates the transcription of *SIC1* at the M/G1 phase boundary and in the G1 phase [49]

$$v_{pSic1} = kk_{sc1} + kkk_{sc1}[Swi5], \tag{21}$$

where the first term represents the basal production of Sic1, and the second term represents the transcriptional regulation of *SIC1* by Swi5. Regulation of Swi5 is described by Eq.(53) of Section 4.

(ii) Sic1 binds to the B-type cyclin complexes (Cdc28-Clb5 and Cdc28-Clb2) to inhibit their activity in the G1 phase. We modelled these associations by mass-action kinetics:

$$\begin{aligned} v_{pC5} &= k_{asb5}[Clb5][Sic1], \\ v_{pC2} &= k_{asb2}[Clb2][Sic1], \end{aligned} \tag{22}$$

where k_{asb5} and k_{asb2} are the rates of Sic1 binding to Cdc28-Clb5 and Cdc28-Clb2 to build C5 and C2 complexes, respectively.

We also assumed that Sic1 binds to Cdc28P-Clb2 denoted by PClb2, using mass-action kinetics to model this reaction:

$$v_{pPTrim} = k_{pasb2}[PClb2][Sic1], \tag{23}$$

where k_{pasb2} captures the association rate of Sic1 to PClb2.

(iii) To enable the G1-to-S transition, Sic1 has to be inactivated. Cdc28 phosphorylates and consequently inactivates Sic1 [9]. We modelled the phosphorylation of Sic1 by Cdc28, considering the total concentration of Cdc28, which is bound to cyclins:

$$v_{pSic1P} = V_{kpc1}[Sic1], \tag{24}$$

where V_{kpc1} is described by Eq.(102) of Section 4. V_{kpc1} is a function of the total Cdc28 concentration (represented by cyclin concentration in our model) and the total concentration of Sic1.

(iv) During the M phase, active APC complexes degrade the B-type cyclins (Clb5, Clb2). These degradations cause the release of Sic1 from the C5 and C2 complexes in addition to spontaneous disassociation of Sic1 from these two complexes. We modelled the freeing of Sic1 from C2 and C5 complex by the following equations:

$$\begin{aligned} v_{aSic1C5} &= (V_{db5} + k_{dib5})\,[C5], \\ v_{aSic1C2} &= (V_{db2} + k_{dib2})\,[C2], \end{aligned} \tag{25}$$

where $V_{db5} = kk_{db5} + kkk_{db5}[Cdc20_A]$ and $V_{db2} = kk_{db2} + kkk_{db2}[Cdh1] + k_{db2p}[Cdc20_A]$ capture the dynamics of APC dependent release of Sic1 from C5 and C2, and k_{dib5} and k_{dib2} are the disassociation rates of Sic1 from C5 and C2, respectively.

We assumed that the same mechanisms freeing Sic1 apply to the PTrim (Sic1-Cdc28P-Clb2) complex. The following equation describes our model for the dissociation of Sic1 from PTrim and also the release of Sic1 from PTrim when Clb2 is degraded by APC dependent mechanisms:

$$v_{aSic1PTrim} = V_{pdb2}[PTrim] + k_{pdib2}[PTrim], \tag{26}$$

where $V_{pdb2} = V_{db2} = kk_{db2} + kkk_{db2}[Cdh1] + k_{db2p}[Cdc20_A]$.

(v) Protein phosphatase Cdc14 is required for exit from mitosis. Cdc14 dephosphorylates Sic1. We modelled this dephosphorylation by mass-action kinetics. Regulation of Cdc14 is described by Eq.(62) of Section 4.

$$v_{pSic1Sic1P} = k_{ppc1}[Cdc14][Sic1P], \tag{27}$$

where k_{ppc1} is the dephosphorylation rate of Sic1P by Cdc14.

(vi) Hog1PP directly phosphorylates Sic1 [20]. This phosphorylation reduces the binding of Sic1 to the SCF complex and thus hinders efficient Sic1 degradation [20]. Phosphorylation of Sic1 by Hog1PP occurs at its Thr173 site, which is different from the Cdc28-dependent phosphorylation site. Therefore, we distinguish between the Sic1 which is phosphorylated by Cdc28 (Sic1P) and the one which is phosphorylated by Hog1PP (Sic1h). Sic1h is more stable compared with Sic1P [20]. We used the following Hill function to model the phosphorylation of Sic1 by Hog1PP:

$$v_{iSic1Hog1PP} = \frac{kk_{ash}[Sic1][Hog1PP]^{n_{Hog1Sic1}}}{kkk_{ash1}^{n_{Hog1Sic1}} + [Hog1PP]^{n_{Hog1Sic1}}}, \tag{28}$$

where k_{ash1} corresponds to maximal level of Sic1h, and kkk_{ash1} represents the concentration of Hog1PP needed to phosphorylate Sic1.

We assumed that Cdc14 is the phosphatase that reverses the phosphorylation of Sic1 by Hog1PP. This is a reasonable choice, since Cdc14 has many substrates in a cell and has been reported to dephosphorylate many Cdc28-Clb substrates [24]. We used mass-action kinetics to model this dephosphorylation:

$$v_{pSic1Sic1h} = k_{h1ppc1}[Cdc14][Sic1h], \tag{29}$$

where k_{h1ppc1} is the dephosphorylation rate of Sic1h by Cdc14.

By similar arguments we modelled the regulation of Sic1P and Sic1h. We also assumed that simultaneous phosphorylation of Sic1 by Cdc28 and Hog1PP is possible, the product of which is denoted by Sic1hP.

$$
\begin{aligned}
\frac{d[Sic1]}{dt} &= v_{pSic1} + v_{aSic1C5} + v_{aSic1C2} + v_{aSic1PTrim} + v_{pSic1Sic1P} + v_{pSic1Sic1h} \\
&\quad -v_{pC5} - v_{pC2} - v_{pPTrim} - v_{pSic1P} - v_{iSic1Hog1PP} \\
&= (kk_{sc1} + kkk_{sc1}[Swi5]) + (V_{db5} + k_{dib5})[C5] \\
&\quad + (V_{db2} + k_{dib2})[C2] + (V_{pdb2} + k_{pdib2})[PTrim] \\
&\quad + (k_{ppc1}[Sic1P] + k_{h1ppc1}[Sic1h])[Cdc14] \\
&\quad - (k_{asb2}[Clb2] + k_{asb5}[Clb5] + k_{pasb2}[PClb2] + V_{kpc1})[Sic1] \\
&\quad - \frac{kk_{ash}[Sic1][Hog1PP]^{n_{Hog1Sic1}}}{kkk_{ash1}^{n_{Hog1Sic1}} + [Hog1PP]^{n_{Hog1Sic1}}}.
\end{aligned} \tag{30}
$$

$$
\begin{aligned}
\frac{d[Sic1P]}{dt} &= V_{kpc1}[Sic1] + V_{db2}[C2P] + V_{db5}[C5P] + V_{pdb2}[PTrimP] \\
&\quad + k_{h1ppc1}[Sic1hP][Cdc14] - (k_{ppc1}[Cdc14] + k_{d3c1})[Sic1P] \\
&\quad - \frac{kk_{ash1}[Sic1P][Hog1PP]^{n_{Hog1Sic1}}}{kkk_{ash1}^{n_{Hog1Sic1}} + [Hog1PP]^{n_{Hog1Sic1}}}.
\end{aligned} \tag{31}
$$

$$
\begin{aligned}
\frac{d[Sic1h]}{dt} &= \frac{kk_{ash}[Sic1][Hog1PP]^{n_{Hog1Sic1}}}{kkk_{ash1}^{n_{Hog1Sic1}} + [Hog1PP]^{n_{Hog1Sic1}}} \\
&\quad + V_{hdb2}[C2h] + k_{hdib2}[C2h] + V_{hdb5}[C5h] + k_{hdib5}[C5h] + k_{hppc1}[Cdc14][Sic1hP] \\
&\quad - (k_{hasb2}[Clb2] + k_{hasb5}[Clb5] + V_{hkpc1} + k_{hpasb2}[PClb2] + k_{h1ppc1}[Cdc14])[Sic1h] \\
&\quad + (k_{hpdib2} + V_{hpdb2})[PTrimh].
\end{aligned} \tag{32}
$$

$$
\begin{aligned}
\frac{d[Sic1hP]}{dt} &= V_{hkpc1}[Sic1h] - (k_{hppc1}[Cdc14] + k_{hd3c1})[Sic1hP] \\
&\quad + V_{hdb2}[C2hP] + V_{hdb5}[C5hP] + V_{hpdb2}[PTrimhP] - k_{h1ppc1}[Sic1hP][Cdc14] \\
&\quad + \frac{kk_{ash1}[Sic1P][Hog1PP]^{n_{Hog1Sic1}}}{kkk_{ash1}^{n_{Hog1Sic1}}[Hog1PP]^{n_{Hog1Sic1}}}.
\end{aligned} \tag{33}
$$

3 Sensitivity analysis of the estimated parameters

We studied the influence of the estimated parameters on the delay duration upon application of 1 M NaCl osmostress at different time points throughout the cell cycle. We focussed on the set of new parameters which were added to the model to link the osmotic stress response to the cell cycle, since the rest of the parameters was available from the literature [4, 20, 40, 41, 43, 46, 50–54].

To investigate the sensitivity of the parameters we took the following approach: (i) we chose a time point in each of the phases for application of 1 M NaCl ($t = 20$ minutes for the G1 phase, $t = 50$ minutes for the S phase, $t = 75$ minutes for the DNA re-replication window and $t = 90$ minutes for the M phase). (ii) At each time point we generated 100 sets of randomly chosen parameters uniformly distributed in the interval from 0.1 to 10 times the value in Table S2 (this is done for the parameters involved in the interaction between Hog1PP and the cell cycle components active during each of the investigated phases, highlighted in Table S2. For example, for the G1 phase we randomly vary the 22 parameters describing the interaction of Hog1PP with Sic1, Cln2 and Cln3.) (iii) Finally, we calculated the change in delay duration with respect to the estimated set of parameters, namely $\Delta_\tau = \tau_o - \tau_r$, where τ_o is the delay duration caused by 1 M NaCl for the estimated set of parameters (Table S2) and τ_r is the delay duration caused by 1 M NaCl with the random set of parameters. The results for each of phases are shown in

Figure S8. The y-axis shows $\frac{\Delta_\tau}{|\tau_o|}$ and the x-axis represents the sets of randomly chosen parameters (100 sets for each phase). If the cell cycle duration obtained for one of the randomly chosen set of parameters was longer than 300 min, we stopped the simulation and represented it by a red point. The results for each of the phases show that the obtained delay duration is very robust against variations in the parameters (see Figures S8A (G1 phase), S8B (S phase), S8C (DNA re-replication window) and S8D (M phase)). This is especially the case for the G1 and S phase. In the case of the DNA re-replication window, there are a few random sets which yield a non-dividing cell, but the general results show that the predicted delay is very robust. The most sensitive parameters seem to be the ones related to the M phase. Note that with our set of estimated parameters, our model predicts an acceleration of the cell cycle, instead of an arrest, if stress is applied during the M phase. 37% of the chosen random sets show an arrest of the cell cycle instead of an acceleration, indicating the sensitivity of the chosen parameters in the M phase. In all those sets, the parameter governing the downregulation of *CLB2* by Hog1PP happen to be very small compared with the estimated value, showing that the prediction of accelerated exit from mitosis is very sensitive to the value of this parameter.

4 Equations

$$\frac{d[mass]}{dt} = \frac{k_g}{1+k_{dHog1mass}[Hog1PP]}[mass]\left(M_{mass,max}-[mass]\right) \tag{34}$$

$$\frac{d[Cln2]}{dt} = \frac{(kk_{sn2}+kkk_{sn2}[SBF])\,([mass])}{1+(k_{dHog1Cln2}[Hog1PP])^{n_{Hog1Cln2}}} - k_{dn2}[Cln2] \tag{35}$$

$$\begin{aligned}\frac{d[Clb5]}{dt} = {} & \frac{(kk_{sb5}+kkk_{sb5}[MBF])\,[mass]}{1+(k_{dHog1Clb5}[Hog1PP])^{n_{Hog1Clb5}}} \\ & +(k_{dib5}[C5]+k_{d3c1}[C5P])+(k_{dif5}[F5]+k_{d3f6}[F5P]) \\ & +(k_{hdib5}[C5h]+k_{hd3c1}[C5hP]) \\ & -(V_{db5}+k_{asb5}[Sic1]+k_{asf5}[Cdc6]+k_{hasb5}[Sic1h])\,[Clb5]\end{aligned} \tag{36}$$

$$\begin{aligned}\frac{d[Clb2]}{dt} = {} & \frac{(kk_{sb2}+kkk_{sb2}[Mcm1])\,[mass]}{1+(k_{dHog1Clb2}[Hog1PP])^{n_{Hog1Clb2}}} \\ & +(k_{dib2}[C2]+k_{d3c1}[C2P])+(k_{hdib2}[C2h]+k_{hd3c1}[C2hP]) \\ & +(k_{dif2}[F2]+k_{d3f6}[F2P]) \\ & -(V_{db2}+k_{asb2}[Sic1]+k_{asf2}[Cdc6]+k_{hasb2}[Sic1h])\,[Clb2] \\ & +(K_{Mih1}[PClb2]-K_{Swe1}[Clb2])\end{aligned} \tag{37}$$

$$\begin{aligned}\frac{d[Sic1]}{dt} = {} & (kk_{sc1}+kkk_{sc1}[Swi5])+(V_{db2}+k_{dib2})\,[C2] \\ & +(V_{db5}+k_{dib5})\,[C5]+k_{ppc1}[Cdc14][Sic1P] \\ & -(k_{asb2}[Clb2]+k_{asb5}[Clb5]+V_{kpc1})\,[Sic1] \\ & -\frac{kk_{ash}[Sic1][Hog1PP]^{n_{Hog1Sic1}}}{kkk_{ash1}^{n_{Hog1Sic1}}+[Hog1PP]^{n_{Hog1Sic1}}} \\ & -k_{pasb2}[Sic1][PClb2]+k_{h1ppc1}[Sic1h][Cdc14] \\ & +(V_{pdb2}+k_{pdib2})\,[PTrim]\end{aligned} \tag{38}$$

$$\begin{aligned}\frac{d[Sic1P]}{dt} = {} & V_{kpc1}[Sic1]-(k_{ppc1}[Cdc14]+k_{d3c1})\,[Sic1P] \\ & +V_{db2}[C2P]+V_{db5}[C5P]+V_{pdb2}[PTrimP] \\ & -\frac{kk_{ash1}[Sic1P][Hog1PP]^{n_{Hog1Sic1}}}{kkk_{ash1}^{n_{Hog1Sic1}}+[Hog1PP]^{n_{Hog1Sic1}}}+k_{h1ppc1}[Sic1hP][Cdc14]\end{aligned} \tag{39}$$

$$\begin{aligned}\frac{d[Sic1h]}{dt} = {} & \frac{kk_{ash}[Sic1][Hog1PP]^{n_{Hog1Sic1}}}{kkk_{ash1}^{n_{Hog1Sic1}}+[Hog1PP]^{n_{Hog1Sic1}}} \\ & +(V_{hdb2}+k_{hdib2})\,[C2h]+(V_{hdb5}+k_{hdib5})\,[C5h] \\ & +(k_{hppc1}[Sic1hP]-k_{h1ppc1}[Sic1h])\,[Cdc14] \\ & -(k_{hasb2}[Clb2]+k_{hasb5}[Clb5]+V_{hkpc1})\,[Sic1h] \\ & +k_{hpdib2}[PTrimh]+V_{hpdb2}[PTrimh] \\ & -k_{hpasb2}[Sic1h][PClb2]\end{aligned} \tag{40}$$

$$\begin{aligned}\frac{d[Sic1hP]}{dt} = {} & V_{hkpc1}[Sic1h]-(k_{hppc1}[Cdc14]+k_{hd3c1})\,[Sic1hP] \\ & +V_{hdb2}[C2hP]+V_{hdb5}[C5hP]+V_{hpdb2}[PTrimhP] \\ & +\frac{kk_{ash1}[Sic1P][Hog1PP]^{n_{Hog1Sic1}}}{kkk_{ash1}^{n_{Hog1Sic1}}+[Hog1PP]^{n_{Hog1Sic1}}}-k_{h1ppc1}[Sic1hP][Cdc14]\end{aligned} \tag{41}$$

$$\frac{d[C2]}{dt} = k_{asb2}[Clb2][Sic1] + k_{ppc1}[Cdc14][C2P] - (k_{dib2} + V_{db2} + V_{kpc1})[C2] - K_{Swe1}[C2] + K_{Mih1}[PTrim] + k_{h1ppc1}[C2h][Cdc14] - \frac{kk_{ash1}[C2][Hog1PP]^{n_{Hog1Sic1}}}{kkk_{ash1}^{n_{Hog1Sic1}} + [Hog1PP]^{n_{Hog1Sic1}}} \quad (42)$$

$$\frac{d[C5]}{dt} = k_{asb5}[Clb5][Sic1] + k_{ppc1}[Cdc14][C5P] - (k_{dib5} + V_{db5} + V_{kpc1})[C5] + k_{h1ppc1}[C5h][Cdc14] - \frac{kk_{ash1}[C5][Hog1PP]^{n_{Hog1Sic1}}}{kkk_{ash1}^{n_{Hog1Sic1}} + [Hog1PP]^{n_{Hog1Sic1}}} \quad (43)$$

$$\frac{d[C2P]}{dt} = V_{kpc1}[C2] - (k_{ppc1}[Cdc14] + k_{d3c1} + V_{db2})[C2P] - K_{Swe1}[C2P] + K_{Mih1}[PTrimP] + k_{h1ppc1}[C2hP][Cdc14] - \frac{kk_{ash1}[C2P][Hog1PP]^{n_{Hog1Sic1}}}{kkk_{ash1}^{n_{Hog1Sic1}} + [Hog1PP]^{n_{Hog1Sic1}}} \quad (44)$$

$$\frac{d[C5P]}{dt} = V_{kpc1}[C5] - (k_{ppc1}[Cdc14] + k_{d3c1} + V_{db5})[C5P] - \frac{kk_{ash1}[C5P][Hog1PP]^{n_{Hog1Sic1}}}{kkk_{ash1}^{n_{Hog1Sic1}} + [Hog1PP]^{n_{Hog1Sic1}}} + k_{h1ppc1}[C5hP][Cdc14] \quad (45)$$

$$\frac{d[Cdc6]}{dt} = (kk_{sf6} + kkk_{sf6}[Swi5] + kkkk_{sf6}[SBF]) + (V_{db2} + k_{dif2})[F2] + (V_{db5} + k_{dif5})[F5] + k_{ppf6}[Cdc14][Cdc6P] - (k_{asf2}[Clb2] + k_{asf5}[Clb5] + V_{kpf6} + k_{pasf2}[PClb2])[Cdc6] + (V_{pdb2} + k_{pdif2})[PF2] \quad (46)$$

$$\frac{d[Cdc6P]}{dt} = V_{kpf6}[Cdc6] - (k_{ppf6}[Cdc14] + k_{d3f6})[Cdc6P] + V_{db2}[F2P] + V_{db5}[F5P] + V_{pdb2}[PF2P] \quad (47)$$

$$\frac{d[F2]}{dt} = k_{asf2}[Clb2][Cdc6] + k_{ppf6}[Cdc14][F2P] - (k_{dif2} + V_{db2} + V_{kpf6})[F2] - K_{Swe1}[F2] + K_{Mih1}[PF2] \quad (48)$$

$$\frac{d[F5]}{dt} = k_{asf5}[Clb5][Cdc6] + k_{ppf6}[Cdc14][F5P] - (k_{dif5} + V_{db5} + V_{kpf6})[F5] \quad (49)$$

$$\frac{d[F2P]}{dt} = V_{kpf6}[F2] - (k_{ppf6}[Cdc14] + k_{d3f6} + V_{db2})[F2P] - K_{Swe1}[F2P] + K_{Mih1}[PF2P] \quad (50)$$

$$\frac{d[F5P]}{dt} = V_{kpf6}[F5] - (k_{ppf6}[Cdc14] + k_{d3f6} + V_{db5})[F5P] \quad (51)$$

$$\frac{d[Swi5_T]}{dt} = kk_{sswi} + kkk_{sswi}[Mcm1] - k_{dswi}[Swi5_T] \quad (52)$$

$$\frac{d[Swi5]}{dt} = kk_{sswi} + kkk_{sswi}[Mcm1] + k_{aswi}[Cdc14]([Swi5_T] - [Swi5]) - (k_{dswi} + k_{iswi}[Clb2])[Swi5] \quad (53)$$

$$\frac{d[APC_P]}{dt} = \frac{k_{aapc}[Clb2]\left(1-[APC_P]\right)}{J_{aapc}+1-[APC_P]} - \frac{k_{iapc}[APC_P]}{J_{iapc}+[APC_P]} \quad (54)$$

$$\frac{d[Cdc20_T]}{dt} = kk_{s20} + kkk_{s20}[Mcm1] - k_{d20}[Cdc20_T] \quad (55)$$

$$\frac{d[Cdc20_A]}{dt} = \left(kk_{a20} + kkk_{a20}[APC_P]\right)\left([Cdc20_T]-[Cdc20_A]\right) - \left(k_{mad2}+k_{d20}\right)[Cdc20_A] \quad (56)$$

$$\frac{d[Cdh1]}{dt} = k_{scdh} - k_{dcdh}[Cdh1] + \frac{V_{acdh}\left([Cdh1_T]-[Cdh1]\right)}{J_{acdh}+[Cdh1_T]-[Cdh1]} - \frac{V_{icdh}[Cdh1]}{J_{icdh}+[Cdh1]} \quad (57)$$

$$\frac{d[Cdh1_T]}{dt} = k_{scdh} - k_{dcdh}[Cdh1_T] \quad (58)$$

$$\frac{d[Tem1]}{dt} = \frac{k_{lte1}\left([Tem1_T]-[Tem1]\right)}{J_{atem}+Tem1_T-Tem1} - \frac{k_{bub2}[Tem1]}{J_{item}+[Tem1]} \quad (59)$$

$$\frac{d[Cdc15]}{dt} = -k_{i15}[Cdc15] + \left(kk_{a15}\left([Tem1_T]-[Tem1]\right)\right)\left([Cdc15_T]-[Cdc15]\right) + \left(kkk_{a15}[Tem1]+kkkk_{a15}[Cdc14]\right)\left([Cdc15_T]-[Cdc15]\right) \quad (60)$$

$$\frac{d[Cdc14_T]}{dt} = k_{s14} - k_{d14}[Cdc14_T] \quad (61)$$

$$\frac{d[Cdc14]}{dt} = k_{s14} - k_{d14}[Cdc14] + k_{dnet}\left([RENT]+[RENTP]\right) + k_{dirent}[RENT] + k_{direntp}[RENTP] - \left(k_{asrent}[Net1]+k_{asrentp}[Net1P]\right)[Cdc14] \quad (62)$$

$$\frac{d[Net1_T]}{dt} = k_{snet} - k_{dnet}[Net1_T] \quad (63)$$

$$\frac{d[Net1]}{dt} = k_{snet} - k_{dnet}[Net1] + k_{d14}[RENT] + k_{dirent}[RENT] - k_{asrent}[Cdc14][Net1] + V_{ppnet}[Net1P] - V_{kpnet}[Net1] \quad (64)$$

$$\frac{d[RENT]}{dt} = -\left(k_{d14}+k_{dnet}\right)[RENT] - k_{dirent}[RENT] + k_{asrent}[Cdc14][Net1] - V_{kpnet}[RENT] + V_{ppnet}[RENTP] \quad (65)$$

$$\frac{d[PPX]}{dt} = k_{sppx} - V_{dppx}[PPX] \quad (66)$$

$$\frac{d[Pds1]}{dt} = kk_{spds} + kkk_{s1pds}[SBF] + kkk_{s2pds}[Mcm1] + k_{diesp}[PE] - \left(V_{dpds}+k_{asesp}[Esp1]\right)[Pds1] \quad (67)$$

$$\frac{d[Esp1]}{dt} = -k_{asesp}[Pds1][Esp1] + \left(k_{diesp}+V_{dpds}\right)[PE] \quad (68)$$

$$\frac{d[ORI]}{dt} = k_{sori}\left(\epsilon_{orib5}[Clb5]+\epsilon_{orib2}[Clb2]\right) - k_{dori}[ORI] \quad (69)$$

$$\frac{d[BUD]}{dt} = k_{sbud}\left(\epsilon_{budn2}[Cln2] + \epsilon_{budn3}[Cln3] + \epsilon_{budb5}[Clb5]\right) - k_{dbud}[BUD] \quad (70)$$

$$\frac{d[SPN]}{dt} = k_{sspn}\frac{[Clb2]}{Jspn + [Clb2]} - k_{dspn}[SPN] \quad (71)$$

$$\frac{d[Swe1]}{dt} = k_{sswe}[SBF] + k_{ssweC} - \frac{k_{hsl1}[Hsl1Hsl7][Swe1]}{J_{iwee} + [Swe1]} + k_{hsl1r}[Swe1M] - \frac{V_{iwee}[Clb2][Swe1]}{J_{iwee} + [Swe1]} + \frac{V_{awee}[Swe1P]}{J_{awee} + [Swe1P]} - kk_{dswe}[Swe1] \quad (72)$$

$$\frac{d[Swe1P]}{dt} = -\frac{k_{hsl1}[Hsl1Hsl7][Swe1P]}{J_{iwee} + [Swe1P]} + k_{hsl1r}[Swe1MP] - \frac{V_{awee}[Swe1P]}{J_{awee} + [Swe1P]} - kk_{dswe}[Swe1P] + \frac{V_{iwee}[Clb2][Swe1]}{J_{iwee} + [Swe1]} \quad (73)$$

$$\frac{d[Hsl1Hsl7]}{dt} = \frac{kk_{Hsl1Hsl7}[BUD]}{1 + \left(\frac{[Hog1PP]}{J_{Hsl1}}\right)^{n_{Hsl1d}}} - kkk_{Hsl1Hsl7}V_{db2}[Hsl1Hsl7] - kkd_{Hsl1Hsl7}[Hsl1_Hsl7] \quad (74)$$

$$\frac{d[Mih1]}{dt} = \frac{Va_{mih}[Clb2]\left([Mih1_T] - [Mih1]\right)}{Ja_{mih} + [Mih1_T] - [Mih1]} - \frac{Vi_{mih}[Mih1]}{Ji_{mih} + [Mih1]} \quad (75)$$

$$\frac{d[PClb2]}{dt} = K_{Swe1}[Clb2] - K_{Mih1}[PClb2] + k_{pd3c1}[PTrimP] + k_{pdib2}[PTrim] + k_{pd3f6}[PF2P] + k_{pdif2}[PF2] - V_{pdb2}[PClb2] - k_{pasb2}[Sic1][PClb2] - k_{pasf2}[Cdc6][PClb2] + k_{hpd3c1}[PTrimhP] + k_{hpdib2}[PTrimh] - k_{hpasb2}[PClb2][Sic1h] \quad (76)$$

$$\frac{d[PTrim]}{dt} = k_{pasb2}[Sic1][PClb2] - k_{pdib2}[PTrim] + K_{Swe1}[C2] + k_{pppc1}[Cdc14][PTrimP] - V_{pdb2}[PTrim] - V_{pkpc1}[PTrim] - K_{Mih1}[PTrim] + k_{h1ppc1}[Cdc14][PTrimh] - \frac{kk_{ash1}[PTrim][Hog1PP]^{n_{Hog11Sic1}}}{kkk_{ash1}^{n_{Hog1Sic1}} + [Hog1PP]^{n_{Hog1Sic1}}} \quad (77)$$

$$\frac{d[PTrimP]}{dt} = -k_{pd3c1}[PTrimP] - k_{pppc1}[Cdc14][PTrimP] + V_{pkpc1}[PTrim] - V_{pdb2}[PTrimP] - K_{Mih1}[PTrimP] + K_{Swe1}[C2P] - \frac{kk_{ash1}[PTrimP][Hog1PP]^{n_{Hog1Sic1}}}{kkk_{ash1}^{n_{Hog1Sic1}} + [Hog1PP]^{n_{Hog1Sic1}}} + k_{h1ppc1}[Cdc14][PTrimhP] \quad (78)$$

$$\frac{d[PF2]}{dt} = -k_{pdif2}[PF2] + k_{pasf2}[PClb2][Cdc6] + k_{pppf6}[Cdc14][PF2P] - V_{pdb2}[PF2] - V_{pkpf6}[PF2] + K_{Swe1}[F2] - K_{Mih1}[PF2] \quad (79)$$

$$\frac{d[PF2P]}{dt} = V_{pkpf6}[PF2] - k_{pppf6}[Cdc14][PF2P] - k_{pd3f6}[PF2P] - V_{pdb2}[PF2P] + K_{Swe1}[F2P] - K_{Mih1}[PF2P] \quad (80)$$

$$\frac{d[C5h]}{dt} = k_{hasb5}[Clb5][Sic1h] + k_{hppc1}[Cdc14][C5hP] - \left(k_{hdib5} + V_{hdb5} + V_{hkpc1}\right)[C5h] + \frac{kk_{ash1}[C5][Hog1PP]^{n_{Hog1Sic1}}}{kkk_{ash1}^{n_{Hog1Sic1}} + [Hog1PP]^{n_{Hog1Sic1}}} - k_{h1ppc1}[C5h][Cdc14] \quad (81)$$

$$\frac{d[C2h]}{dt} = k_{hasb2}[Clb2][Sic1h] + k_{hppc1}[Cdc14][C2hP] - (k_{hdib2} + V_{hdb2} + V_{hkpc1})[C2h] - K_{Swe1}[C2h] + K_{Mih1}[PTrimh] - k_{h1ppc1}[C2h][Cdc14] + \frac{kk_{ash1}[C2][Hog1PP]^{n_{Hog1Sic1}}}{kkk_{ash1}^{n_{Hog1Sic1}} + [Hog1PP]^{n_{Hog1Sic1}}} \quad (82)$$

$$\frac{d[C5hP]}{dt} = V_{hkpc1}[C5h] - (k_{hppc1}[Cdc14] + k_{hd3c1} + V_{hdb5})[C5hP] + \frac{kk_{ash1}[C5P][Hog1PP]^{n_{Hog1Sic1}}}{kkk_{ash1}^{n_{Hog1Sic1}} + [Hog1PP]^{n_{Hog1Sic1}}} - k_{h1ppc1}[C5hP][Cdc14] \quad (83)$$

$$\frac{d[C2hP]}{dt} = V_{hkpc1}[C2h] - (k_{hppc1}[Cdc14] + k_{hd3c1} + V_{hdb2})[C2hP] - K_{Swe1}[C2hP] + K_{Mih1}[PTrimhP] - k_{h1ppc1}[C2hP][Cdc14] + \frac{kk_{ash1}[C2P][Hog1PP]^{n_{Hog1Sic1}}}{kkk_{ash1}^{n_{Hog1Sic1}} + [Hog1PP]^{n_{Hog1Sic1}}}$$

$$\frac{d[PTrimh]}{dt} = k_{hpasb2}[Sic1h][PClb2] - k_{hpdib2}[PTrimh] + k_{hpppc1}[Cdc14][PTrimhP] - V_{hpdb2}[PTrimh] - V_{hpkpc1}[PTrimh] + K_{Swe1}[C2h] - K_{Mih1}[PTrimh] - k_{h1ppc1}[Cdc14][PTrimh] + \frac{kk_{ash1}[PTrim][Hog1PP]^{n_{Hog1Sic1}}}{kkk_{ash1}^{n_{Hog1Sic1}} + [Hog1PP]^{n_{Hog1Sic1}}} \quad (84)$$

$$\frac{d[PTrimhP]}{dt} = -k_{hpd3c1}[PTrimhP] - k_{hpppc1}[Cdc14][PTrimhP] + V_{hpkpc1}[PTrimh] - V_{hpdb2}[PTrimhP] - K_{Mih1}[PTrimhP] + K_{Swe1}[C2hP] - k_{h1ppc1}[Cdc14][PTrimhP] + \frac{kk_{ash1}[PTrimP][Hog1PP]^{n_{Hog1Sic1}}}{kkk_{ash1}^{n_{Hog1Sic1}} + [Hog1PP]^{n_{Hog1Sic1}}} \quad (85)$$

$$\frac{d[Swe1MP]}{dt} = \frac{k_{hsl1}[Hsl1Hsl7][Swe1P]}{J_{iwee} + [Swe1P]} - k_{hsl1r}[Swe1MP] - \frac{V_{awee}[Swe1MP]}{J_{awee} + [Swe1MP]} + \frac{V_{iwee}[Clb2][Swe1M]}{J_{iwee} + [Swe1M]} - kk_{dswe}[Swe1MP] \quad (86)$$

$$\frac{d[Swe1M]}{dt} = \frac{k_{hsl1}[Hsl1Hsl7][Swe1]}{J_iwee + [Swe1]} - k_{hsl1r}[Swe1M] - \frac{V_{iwee}[Clb2][Swe1M]}{J_{iwee} + [Swe1M]} + \frac{V_{awee}[Swe1MP]}{J_{awee} + [Swe1MP]} - kk_{dswe}[Swe1M] \quad (87)$$

$$[Cln3] = \frac{C_0 D_{n3}[mass]}{J_{n3} + (k_{Hog1Cln3} + kd_{Hog1Cln3}[Hog1PP]^{n_{Hog1Cln3}})[Hog1PP]^{n_{Hog1Cln3}} + D_{n3}[mass]} \quad (88)$$

$$[Bck2] = B_0[mass] \quad (89)$$

$$[Clb5_T] = [Clb5] + [C5] + [C5P] + [F5] + [F5P] + [C5h] + [C5hP] \quad (90)$$

$$[Clb2_T] = [Clb2] + [C2] + [C2P] + [F2] + [F2P] + [PClb2] + [PTrim] + [PTrimP] + [PF2] + [PF2P] + [C2h] + [C2hP] + [PTrimh] + [PTrimhP] \quad (91)$$

$$[Sic1_T] = [Sic1] + [Sic1P] + [C2] + [C2P] + [C5] + [C5P] + [PTrim] + [PTrimP] + [Sic1h] + [Sic1hP] + [C2h] + [C2hP] + [C5h] + [C5hP] + [PTrimh] + [PTrimhP] \quad (92)$$

$$[Cdc6_T] = [Cdc6] + [Cdc6P] + [F2] + [F2P] + [F5] + [F5P] + [PF2] + [PF2P] \quad (93)$$

$$[CKI_T] = [Sic1_T] + [Cdc6_T] \quad (94)$$

$$[RENTP] = [Cdc14_T] - [RENT] - [Cdc14] \quad (95)$$

$$[Net1P] = [Net1_T] - [Net1] - [Cdc14_T] + [Cdc14] \quad (96)$$

$$[PE] = [Esp1_T] - [Esp1] \quad (97)$$

$$V_{db5} = kk_{db5} + kkk_{db5}[Cdc20_A] \quad (98)$$

$$V_{db2} = kk_{db2} + kkk_{db2}[Cdh1] + k_{db2p}[Cdc20_A] \quad (99)$$

$$V_{asbf} = k_{asbf}\left(\epsilon_{sbfn2}[Cln2] + \epsilon_{sbfn3}\left([Cln3] + [Bck2]\right) + \epsilon_{sbfb5}[Clb5]\right) \quad (100)$$

$$V_{isbf} = kk_{isbf} + kkk_{isbf}[Clb2] \quad (101)$$

$$V_{kpc1} = k_{d1c1} + k_{d2c1}\frac{\epsilon_{c1n3}[Cln3] + \epsilon_{c1k2}[Bck2] + \epsilon_{c1n2}[Cln2] + \epsilon_{c1b5}[Clb5] + \epsilon_{c1b2}[Clb2]}{J_{d2c1} + [Sic1_T]} \quad (102)$$

$$V_{kpf6} = k_{d1f6} + k_{d2f6}\frac{\epsilon_{f6n3}[Cln3] + \epsilon_{f6k2}[Bck2] + \epsilon_{f6n2}[Cln2] + \epsilon_{f6b5}[Clb5] + \epsilon_{f6b2}[Clb2]}{J_{d2f6} + [Cdc6_T]} \quad (103)$$

$$V_{acdh} = kk_{acdh} + kkk_{acdh}[Cdc14] \quad (104)$$

$$V_{icdh} = kk_{icdh} + kkk_{icdh}\left(\epsilon_{cdhn3}[Cln3] + \epsilon_{cdhn2}[Cln2] + \epsilon_{cdhb2}[Clb2] + \epsilon_{cdhb5}[Clb5]\right) \quad (105)$$

$$V_{ppnet} = kk_{ppnet} + kkk_{ppnet}[PPX] \quad (106)$$

$$V_{kpnet} = \left(kk_{kpnet} + kkk_{kpnet}[Cdc15]\right)[mass] \quad (107)$$

$$V_{dppx} = kk_{dppx} + kkk_{dppx}\left(J_{20ppx} + [Cdc20_A]\right)\frac{J_{pds}}{J_{pds} + [Pds1]} \quad (108)$$

$$V_{dpds} = kk_{d1pds} + kkk_{d2pds}[Cdc20_A] + kkk_{d3pds}[Cdh1] \quad (109)$$

$$K_{Swe1} = kk_{swe}[Swe1] + kkk_{swe}[Swe1M] + kkkk_{swe}[Swe1P] \quad (110)$$

$$K_{Mih1} = kk_{mih1}[Mih1] + kkk_{mih1}\left([Mih1_T] - [Mih1]\right) \quad (111)$$

$$[MBF] = G\left(V_{asbf}, V_{isbf}, J_{asbf}, J_{isbf}\right) \quad (112)$$

$$[SBF] = G\left(V_{asbf}, V_{isbf}, J_{asbf}, J_{isbf}\right) \quad (113)$$

$$[Mcm1] = G\left(k_{amcm}[Clb2], k_{imcm}, J_{amcm}, J_{imcm}\right) \quad (114)$$

$$G\left(V_a, V_i, J_a, J_i\right) = \frac{2J_iV_a}{V_i - V_a + J_aV_i + J_iV_a + \sqrt{\left(V_i - V_a + J_aV_i + JiVa\right)^2 - 4\left(V_i - V_a\right)J_iV_a}} \quad (115)$$

$$V_{hkpc1} = k_{hkpc1}V_{kpc1} \quad (116)$$

$$V_{hpkpc1} = k_{hpkpc1}V_{kpc1} \quad (117)$$

$$V_{pdb2} = V_{db2} \quad (118)$$

$$V_{hdb5} = V_{db5} \quad (119)$$

$$V_{hdb2} = V_{db2} \quad (120)$$

$$V_{hpdb2} = V_{db2} \quad (121)$$

$$V_{pkpf6} = V_{kpf6} \quad (122)$$

$$V_{pkpc1} = V_{kpc1} \quad (123)$$

5 Initial conditions and parameters

Table S 2. Initial conditions for a newborn, wild-type daughter cell in arbitrary units.

$[mass] = 2.201$	$[Cln2] = 5.069 \times 10^{-2}$	$[Clb5] = 7.330 \times 10^{-2}$	$[Clb2] = 3.008 \times 10^{-1}$
$[Sic1] = 1.123 \times 10^{-2}$	$[Sic1P] = 7.408 \times 10^{-3}$	$[C2] = 2.086 \times 10^{-1}$	$[C5] = 4.810 \times 10{-}2$
$[C2P] = 3.994 \times 10{-}2$	$[C5P] = 9.154 \times 10^{-3}$	$[Cdc6] = 5.911 \times 10^{-2}$	$[Cdc6P] = 1.808 \times 10^{-2}$
$[F2] = 2.154 \times 10^{-1}$	$[F5] = 5.007 \times 10^{-5}$	$[F2P] = 4.681 \times 10^{-2}$	$[F5P] = 1.055 \times 10^{-5}$
$[Swi5_T] = 9.870 \times 10^{-1}$	$[Swi5] = 9.532 \times 10^{-1}$	$[APC_P] = 9.676 \times 10^{-2}$	$[Cdc20_T] = 1.965$
$[Cdc20_A] = 4.241 \times 10^{-1}$	$[Cdh1_T] = 1$	$[Cdh1] = 6.675 \times 10^{-1}$	$[Tem1] = 9.778 \times 10^{-1}$
$[Cdc15] = 6.618 \times 10^{-1}$	$[Cdc14_T] = 2$	$[Cdc14] = 4.370 \times 10^{-1}$	$[Net1_T] = 2.8$
$[Net1] = 2.000 \times 10^{-2}$	$[RENT] = 1.014$	$[PPX] = 1.381 \times 10^{-1}$	$[Pds1] = 3.284 \times 10^{-2}$
$[Esp1] = 2.584 \times 10^{-1}$	$[ORI] = 9 \times 10^{-4}$	$[BUD] = 8.5 \times 10^{-3}$	$[SPN] = 3.05 \times 10^{-2}$
$[Hsl1Hsl7] = 8.236 \times 10^{-2}$	$[Swe1P] = 6.066 \times 10^{-4}$	$[Mih1] = 8.625 \times 10^{-1}$	$[PClb2] = 5.246 \times 10^{-4}$
$[PTrim] = 3.789 \times 10^{-4}$	$[PTrimP] = 7.324 \times 10^{-5}$	$[PF2] = 3.902 \times 10^{-4}$	$[PF2P] = 8.528 \times 10^{-5}$
$[Sic1h] = 1.262 \times 10^{-6}$	$[Sic1hP] = 1.096 \times 10^{-5}$	$[C5h] = 7.165 \times 10^{-6}$	$[C2h] = 3.111 \times 10^{-5}$
$[C5hP] = 7.489 \times 10^{-6}$	$[C2hP] = 3.252 \times 10^{-5}$	$[PTrimh] = 6.318 \times 10^{-8}$	$[PTrimhP] = 1.075 \times 10^{-7}$
$[Swe1MP] = 1.829 \times 10^{-2}$	$[Swe1M] = 6.223 \times 10^{-1}$	$[Swe1] = 1.004 \times 10^{-3}$	

Table S 3. Parameters
The dimension of all parameters that start with a lower case k is min^{-1}. All other parameters are dimensionless.

Parameters	References	Parameters	References	Parameters	References
$k_g = 0.007702$	[46]	$kk_{sn2} = 0$	[50]	$kkk_{sn2} = 0.15$	[50]
$k_{dn2} = 0.12$	[55]	$kk_{sb5} = 0.0008$	[50]	$kkk_{sb5} = 0.005$	[50]
$kkk_{db2} = 0.4$	[50]	$k_{d3c1} = 1$	[50]	$k_{d2f6} = 1$	[50]
$kkk_{sswi} = 0.08$	[40]	$kk_{db5} = 0.01$	[50]	$kkk_{db5} = 0.16$	[40]
$kk_{sb2} = 0.001$	[50]	$kkk_{sb2} = 0.04$	[50]	$kk_{db2} = 0.003$	[50]
$k_{db2p} = 0.15$	[50]	$kk_{sc1} = 0.012$	[40]	$kkk_{sc1} = 0.12$	[40]
$k_{d1c1} = 0.01$	[40]	$k_{d2c1} = 1$	[40]	$k_{ppc1} = 4$	[40]
$kk_{sf6} = 0.024$	[40]	$kkk_{sf6} = 0.12$	[40]	$kkkk_{sf6} = 0.004$	[40]
$k_{d1f6} = 0.01$	[40]	$k_{d3f6} = 1$	[40]	$k_{ppf6} = 4$	[40]
$k_{asb5} = 50$	[46]	$k_{dib5} = 0.06$	[46]	$k_{asf5} = 0.01$	[46]
$k_{asb2} = 50$	[46]	$k_{dib2} = 0.05$	[46]	$k_{asf2} = 15$	[46]
$k_{dif2} = 0.5$	[46]	$kk_{sswi} = 0.005$	[46]	$k_{dif5} = 0.01$	[40]
$kk_{s20} = 0.006$	[40]	$k_{dcdh} = 0.01$	[40]	$k_{d14} = 0.1$	[40]
$k_{i15} = 0.5$	[40]	$k_{dswi} = 0.08$	[40]	$k_{aswi} = 2$	[40]
$k_{iswi} = 0.05$	[40]	$k_{aapc} = 0.1$	[40]	$k_{iapc} = 0.15$	[40]
$kkk_{s20} = 0.6$	[40]	$k_{d20} = 0.3$	[40]	$kk_{a20} = 0.05$	[40]
$kkk_{a20} = 0.2$	[40]	$k_{scdh} = 0.01$	[40]	$kk_{acdh} = 0.01$	[40]
$kkk_{acdh} = 0.8$	[40]	$kk_{icdh} = 0.001$	[40]	$kkk_{icdh} = 0.08$	[40]
$k_{s14} = 0.2$	[40]	$k_{snet} = 0.084$	[40]	$k_{dnet} = 0.03$	[40]
$kk_{a15} = 0.002$	[40]	$kkk_{a15} = 1$	[40]	$kkkk_{a15} = 0.001$	[40]
$kk_{ppnet} = 0.05$	[40]	$kkk_{ppnet} = 3$	[40]	$kk_{kpnet} = 0.01$	[40]
$kkk_{kpnet} = 0.6$	[40]	$k_{asrent} = 200$	[40]	$k_{asrentp} = 1$	[40]
$kk_{spds} = 0$	[40]	$k_{asesp} = 50$	[40]	$k_{sspn} = 0.1$	[40]
$k_{imcm} = 0.15$	[40]	$k_{dirent} = 1$	[40]	$k_{direntp} = 2$	[40]
$k_{sppx} = 0.1$	[40]	$kk_{dppx} = 0.17$	[40]	$kkk_{dppx} = 2$	[40]
$kkk_{s1pds} = 0.03$	[40]	$kkk_{s2pds} = 0.055$	[40]	$kk_{d1pds} = 0.01$	[40]
$kkk_{d2pds} = 0.2$	[40]	$kkk_{d3pds} = 0.04$	[40]	$k_{diesp} = 0.5$	[40]
$k_{sori} = 2$	[51]	$k_{dori} = 0.06$	[51]	$k_{sbud} = 0.2$	[52]
$k_{dbud} = 0.06$	[52]	$k_{dspn} = 0.06$	[51]	$k_{asbf} = 0.38$	[4]
$kk_{isbf} = 0.642$	[4]	$kkk_{isbf} = 8$	[3]	$k_{amcm} = 1$	[40]
$\epsilon_{sbfn2} = 2$	[4]	$\epsilon_{sbfn3} = 10$	[53]	$\epsilon_{sbfb5} = 2$	[9]
$\epsilon_{c1n3} = 0.3$	[53]	$\epsilon_{c1n2} = 0.06$	[4]	$\epsilon_{f6b5} = 0.1$	[9]
$J_{icdh} = 0.03$	[40]	$J_{imcm} = 0.1$	[40]	$K_{ez2} = 0.2$	[51]
$M_{mass,max} = 2.5$	this study	$\epsilon_{c1k2} = 0.03$	[53]	$\epsilon_{orib5} = 0.9$	[53]
$\epsilon_{orib2} = 0.45$	[53]	$n_{Hog1Clb2} = 8$	this study	$k_{ash1} = 0.25$	[40]
$\epsilon_{c1b5} = 0.1$	[53]	$\epsilon_{c1b2} = 0.45$	[53]	$\epsilon_{f6n3} = 0.3$	[53]
$\epsilon_{f6n2} = 0.06$	[53]	$\epsilon_{f6k2} = 0.03$	[53]	$\epsilon_{f6b2} = 0.55$	[53]
$\epsilon_{cdhn3} = 0.25$	[53]	$\epsilon_{cdhn2} = 0.4$	[53]	$\epsilon_{cdhb5} = 8$	[53]
$\epsilon_{cdhb2} = 1.2$	[53]	$\epsilon_{budn3} = 0.05$	[53]	$\epsilon_{budn2} = 0.25$	[53]
$\epsilon_{budb5} = 1$	[53]	$C_0 = 0.4$	this study	$D_{n3} = 1$	this study
$J_{d2c1} = 0.05$	[40]	$J_{d2f6} = 0.05$	[40]	$J_{aapc} = 0.1$	[40]
$J_{iapc} = 0.1$	[40]	$J_{acdh} = 0.03$	[40]	$J_{atem} = 0.1$	[40]
$J_{item} = 0.1$	[40]	$J_{asbf} = 0.01$	[43]	$J_{isbf} = 0.01$	[43]
$J_{amcm} = 0.1$	[43]	$J_{spn} = 0.14$	[40]	$J_{20ppx} = 0.15$	[40]
$J_{pds} = 0.04$	[40]	$K_{ez} = 0.3$	[52]	$k_{pd3c1} = k_{d3c1}$	this study
$k_{pdib2} = k_{dib2}$	this study	$k_{pd3f6} = k_{d3f6}$	this study	$k_{pdif2} = k_{dif2}$	this study
$k_{pasb2} = k_{asb2}$	this study	$k_{pasf2} = k_{asf2}$	this study	$k_{pppc1} = k_{ppc1}$	this study
$k_{pppf6} = k_{ppf6}$	this study	$k_{hpd3c1} = 0.01$	this study, [20]	$k_{hpdib2} = k_{d}ib2$	this study
$k_{hpasb2} = k_{asb2}$	this study	$k_{hd3c1} = 0.01$	this study, [20]	$k_{hpppc1} = k_{ppc1}$	this study
$k_{hdib5} = k_{dib5}$	this study	$k_{hasb5} = k_{a}sb5$	this study	$k_{hdib2} = k_{dib2}$	this study
$k_{hasb2} = k_{asb2}$	this study	$k_{hppc1} = k_{ppc1}$	this study	$k_{h1ppc1} = 0.68$	this study
$n_{Hog1Sic1} = 2$	this study	$k_{dHog1Clb2} = 20$	this study	$k_{dHog1Clb5} = 0.01$	this study
$J_{Hsl1} = 0.012$	this study	$n_{Hsl1d} = 8$	this study	$k_{sswe} = 0.006$	[43]
$k_{ssweC} = 0$	[43]	$k_{hsl1} = 0.5$	this study	$k_{hsl1r} = 0.01$	[43]
$V_{awee} = 0.3$	[43]	$V_{iwee} = .2$	[43]	$kk_{dswe} = 0.007$	[43]
$kkk_{dswe} = 0.05$	[43]	$J_{awee} = 0.05$	[43]	$J_{iwee} = 0.0098$	this study
$kk_{swe} = 2$	[43]	$kkk_{swe} = 0.01$	[43]	$kkkk_{swe} = 0.2$	[43]
$kk_{dHog1Cln2} = 4.93$	this study	$kk_{Hsl1Hsl7} = .001$	this study	$kkk_{Hsl1Hsl7} = 0.4$	this study
$kkd_{Hsl1Hsl7} = 0.001$	this study	$k_{dHog1mass} = 2.23$	this study	$Vi_{mih} = 0.3$	[43]
$Va_{mih} = 1$	[43]	$Ji_{mih} = 0.1$	[43]	$Ja_{mih} = 0.1$	[43]
$B_0 = 0.054$	[40]	$kk_{mih1} = 5$	[43]	$kkk_{mih1} = 0.5$	[43]
$k_{hpkpc1} = 14$	this study	$k_{hkpc1} = 14$	this study	$kk_{ash1} = 3.2$	this study
$kkk_{ash1} = 0.2$	this study	$k_{Hog1Cln3} = 20$	this study	$n_{Hog1Cln3} = 2$	this study
$k_{dib5} = 0.06$	[40]	$[Mih1_T] = 1$	[43]	$[Tem1]_T = 1$	[54]
$[Cdc15]_T = 1$	[54]	$[Esp1]_T = 1$	[54]	$kk_{ash} = 0.8$	this study
$J_{n3} = 6$					
$k_{mad2} = 8$ (for $[ORI] > 1$ and $[SPN] < 1$) or 0.01 (otherwise)					
$k_{bub2} = 1$ (for $[ORI] > 1$ and $[SPN] < 1$) or 0.2 (otherwise)					
$k_{lte1} = 1$ (for $[SPN] > 1$ and $[Clb2] < K_{ez}$) or 0.2 (otherwise)					

6 Figures

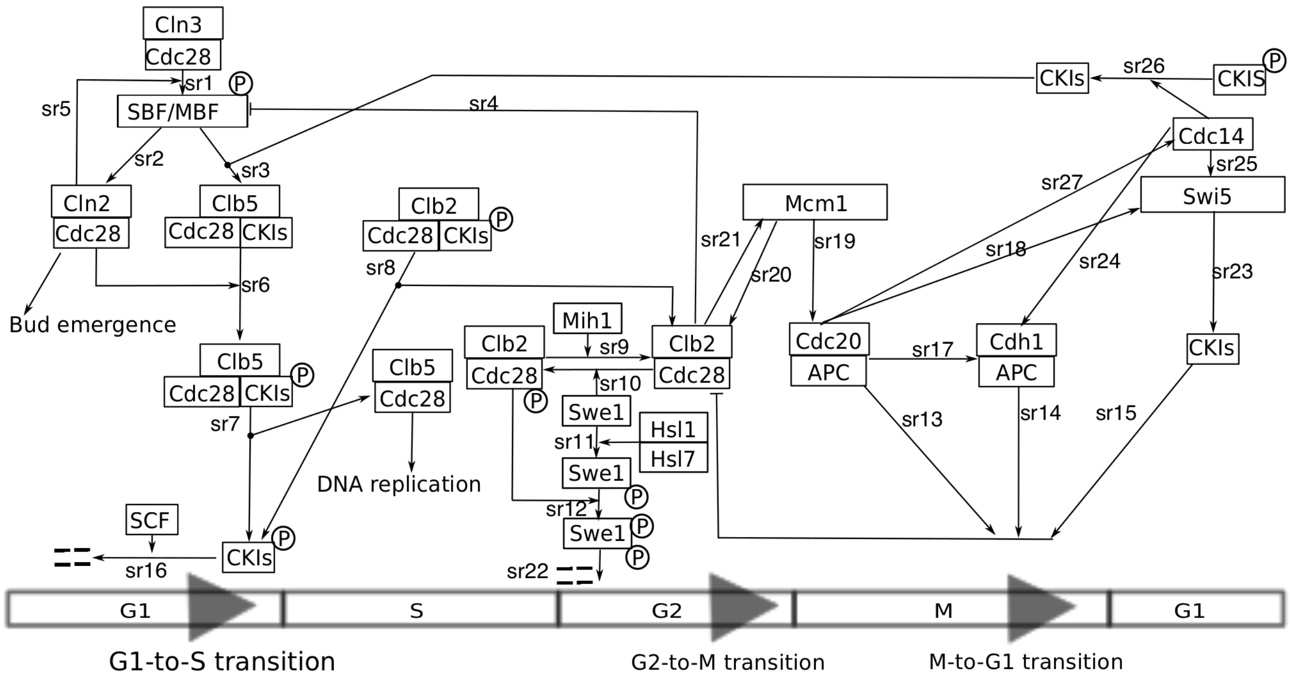

Figure S 1. Simplified schematic of budding yeast cell cycle control network. As soon as cyclins are synthesised, they bind to Cdc28. Since this tethering is fast, we do not show the free cyclins in the figure. CKIs in this figure represent Sic1 and Cdc6.

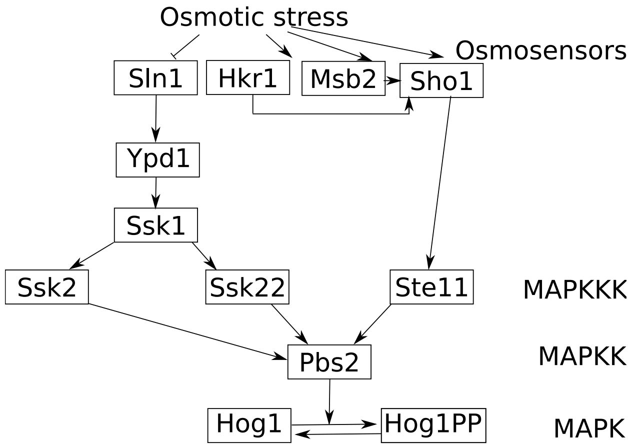

Figure S 2. Osmotic stress response network of *S. cerevisiae.* Osmotic stress is sensed by Sln1, Sho1 and Msb2/Hkr2. They are upstream of two branches which monitor changes in the turgor pressure and, independently, regulate three MAPKKKs (Ste11, Ssk2 and Ssk22). Consequently, MAPKKKs phosphorylate and activate MAPKK (Pbs2). Activation of Pbs2 results in the activation of Hog1 via phosphorylation. Phosphorylated Hog1 then accumulates in the nucleus and activates gene expression of proteins involved in glycerol production. Thereby, glycerol production increases to compensate for turgor pressure loss.

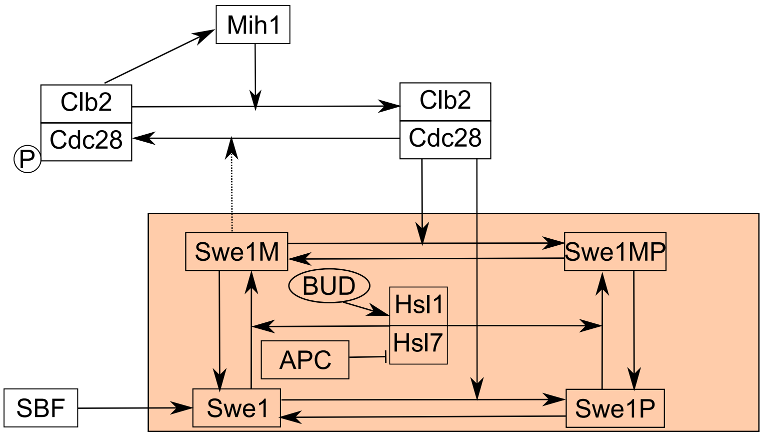

Figure S 3. Simplified schematic diagram of morphogenesis checkpoint: Swe1 kinase phosphorylates Cdc28-Clb2 and halts the transition to the G2/M phase. *SWE1* transcription is activated by the transcription factor SBF. Moreover, Swe1 needs to be phosphorylated in order to become degraded. This phosphorylation occurs via Cdc28-Clb2 and the Hsl1-Hsl7 complex. If the phosphorylation is caused by Cdc28-Clb2, the product is denoted by Swe1P in the diagram, whereas if the phosphorylation is mediated by the Hsl1-Hsl7 complex, it is denoted by Swe1M. The substrate Swe1MP is highly unstable. We assume that the total concentration of Swe1 inhibits the activity of Cdc28-Clb2; see text for further details. The formation of the complex Hsl1-Hsl7 coincides with the formation of the bud. This timing is shown by the variable denoted by BUD in the figure. BUD denotes a mathematical function which describes the observation of bud formation and depends on Cln2, Cln3 and Clb5. The degradation of Hsl1-Hsl7 is APC dependent, which has not been shown in this figure, but it has been considered in the model.

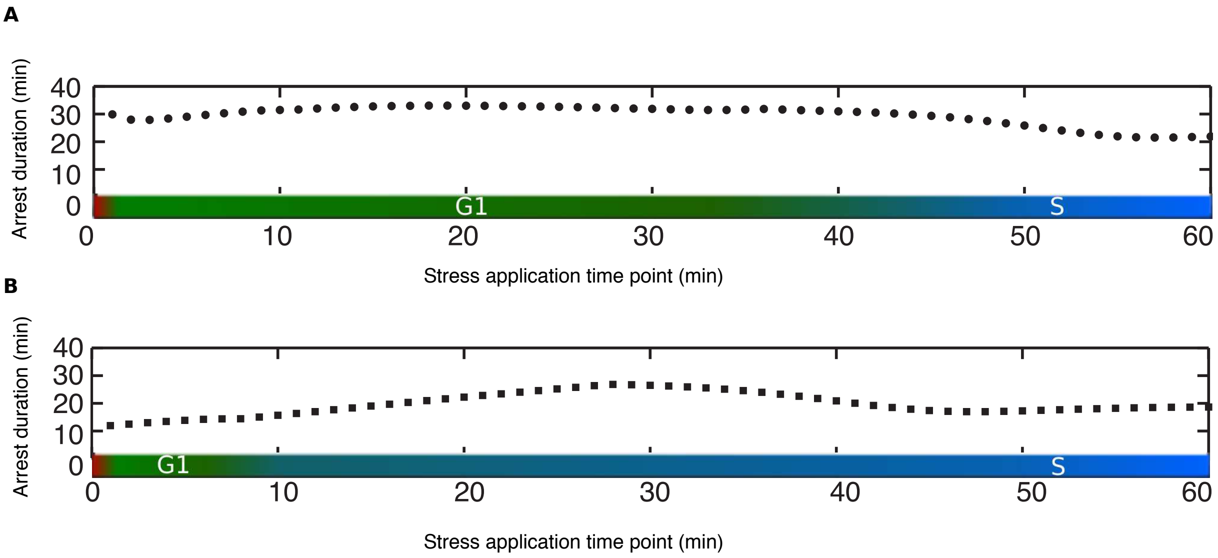

Figure S 4. Investigation of the role of Sic1 on blocking the cell cycle progression when Hog1PP is active. The influence of Sic1 accumulation on the G1 and S phase delay duration has been experimentally investigated, which confirms our simulation results. A) A wild-type cell is subjected to 0.4 M NaCl. B) A *sic*1Δ cell is subjected to 0.4 M NaCl. Deletion of Sic1 makes the G1 phase of the cell shorter in untreated conditions. It also makes the G1 phase cell less adaptable to the osmo-condition, whereas the delay duration for the S phase *sic*1Δ cell is approximately equal to that of wild-type.

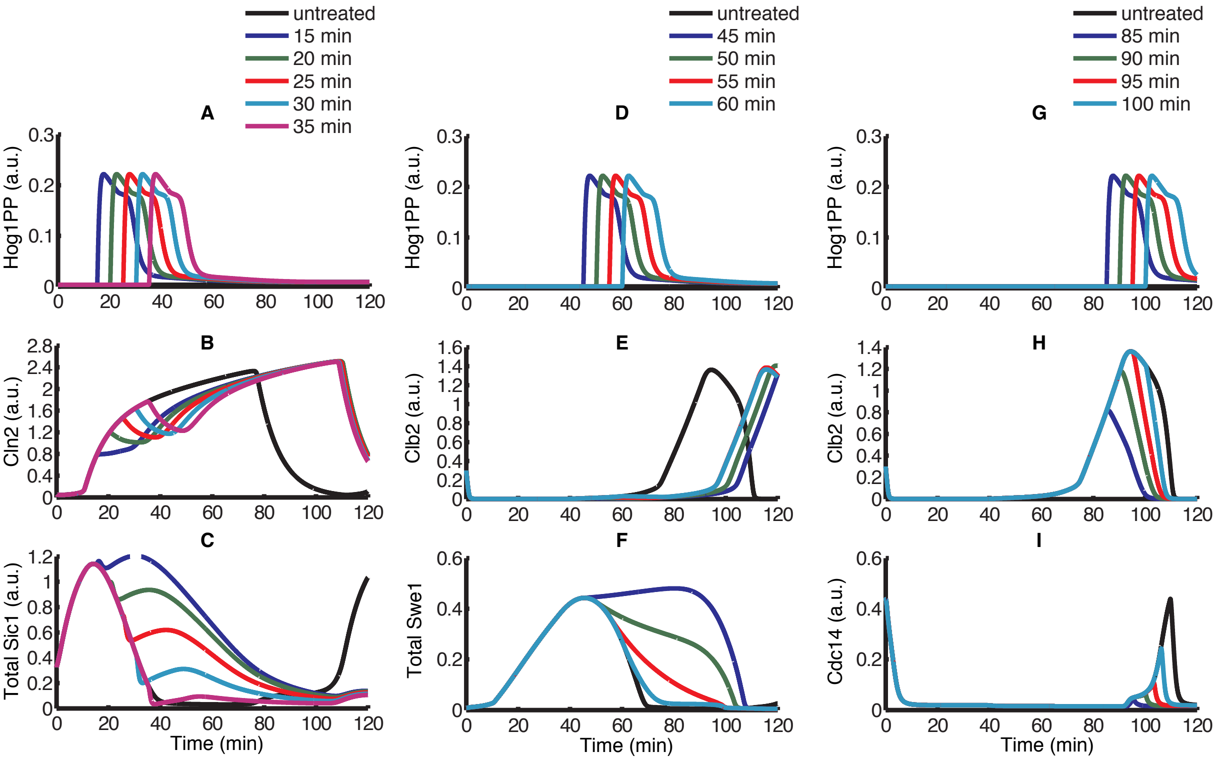

Figure S 5. Model predictions for the activity of Hog1PP and its targets during the G1-to-S (A, B, C), G2-to-M (D, E, F) and M-to-G1 transition (G, H, I). A) 0.4 M NaCl is applied at different time points during the G1-to-S transition to study the influence of stress timing on the profile of Hog1PP. B) Presence of 0.4 M NaCl at different time points during the G1 phase changes the activity profile of Cln2. Salt causes downregulation of Cln2, which is followed by an upregulation when Hog1PP is inactive again. C) Presence of 0.4 M NaCl at different time points during the G1 phase accumulates total Sic1. Moving towards the S phase this accumulation is less pronounced. D) 0.4 M NaCl is applied at different time points during the G2-to-M transition. The time profile of Hog1PP is shown. E) The activation of Clb2 is delayed due to presence of Hog1PP. F) Activity of Hog1PP causes the accumulation of Swe1. G) 0.4 M NaCl is applied at different time points during the M-to-G1 transition. There is no stabilising influence on Clb2 (H) and Cdc14 (I) upon stress, and their levels decrease almost immediately after the onset of stress. The immediate downregulation of Clb2 causes a shorter cell cycle. Moving towards the end of M phase the length of the cell cycle with stress is comparable with the length of the cycle of untreated cells. The levels of the shown proteins are given in arbitrary units (a.u.).

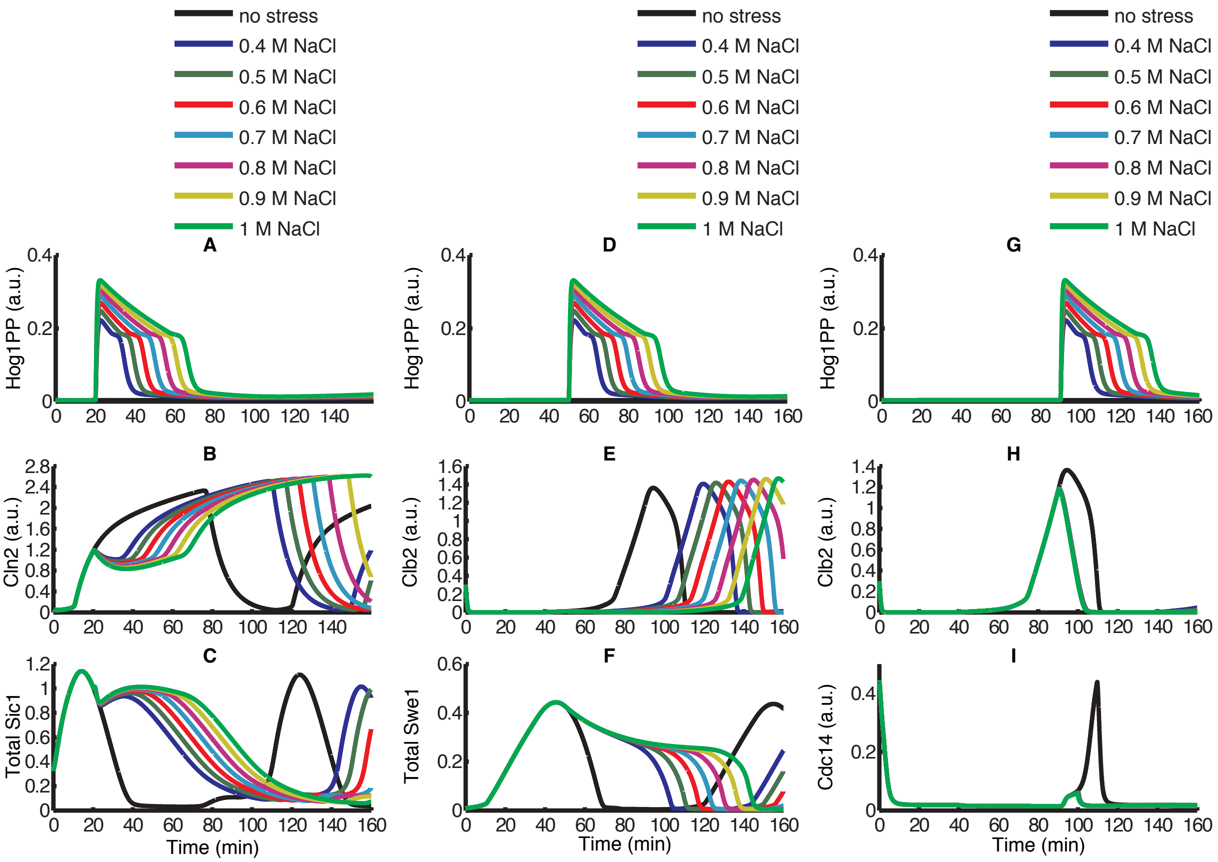

Figure S 6. Model predictions for the influence of the dose of NaCl on the activity of Hog1PP and key cell cycle components during G1-to-S (A, B, C), G2-to-M (D, E, F) and M-to-G1 transition (G, H, I). Time course activity of the wild type untreated cell cycle components are depicted in black. Different concentrations of salt can change the activity duration of Hog1PP. A) Different strengths of stress are applied at a fixed time point which is located near START. B) Hog1PP stabilises the level of Cln2 for a transient duration which is linearly proportional to the dose of stress. C) Higher dose of NaCl causes stronger accumulation of Sic1. D) Different dose of salt is applied to a fixed time point, which is located before G2-to-M transition. E) A higher dose of stress will delay the upregulation of Clb2. F) Higher dose of NaCl causes stronger accumulation of Swe1. The relation between the dose of stress and delay duration is also linear in this interval. G) Different dose of salt is applied to a fixed time point, which is located before M-to-G1 transition. There is no stabilising influence on Clb2 (H) and Cdc14 (I) upon stress, and their levels decrease almost immediately after the onset of the stress. Remarkably, the decrease in Clb2 and Cdc14 occurs in the same way, independently of the stress dose. The levels of the shown proteins are given in arbitrary units (a.u.).

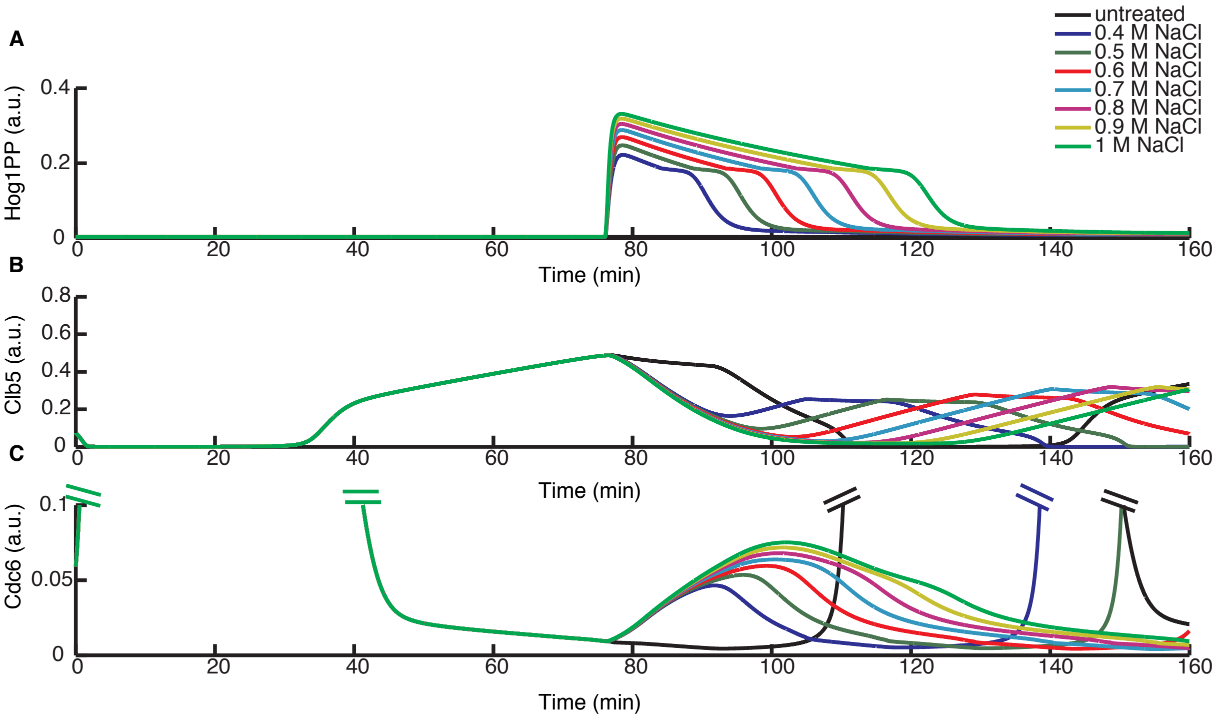

Figure S 7. Model prediction for the influence of the dose of NaCl on the activity of Hog1PP and the cell cycle components during the late S phase or early G2/M phase. Time course activity of the wild type untreated cell cycle components are depicted in black. A) Different concentrations of salt applied at minute 76, change the activity duration of Hog1PP. B) Activity of Hog1PP causes downregulation of Clb5 for a transient duration which is linearly proportional to the dose of stress. C) The Cdc6 levels increase when Clb5 activity is reduced by Hog1PP. This effect is more pronounced for higher stress doses. For illustration purposes the focus is on reactivation of Cdc6 upon presence of Hog1PP. After Hog1PP returns to its basal level, Clb5 starts increasing again. The downregulation, following by an upregulation of Clb5 can lead to DNA re-replication.

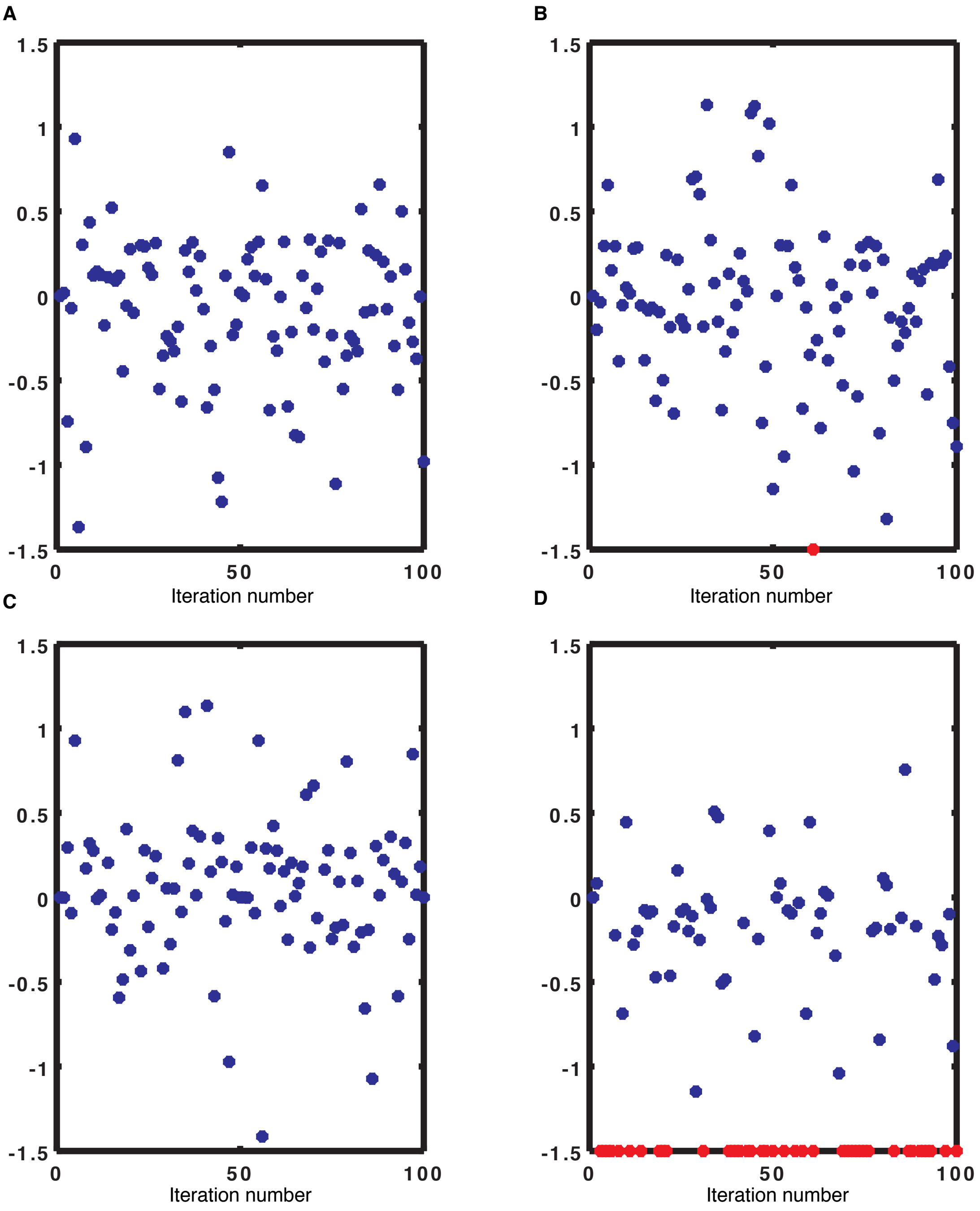

Figure S 8. Parameter sensitivity analysis. We generated 100 sets of randomly chosen parameters in the interval from 0.1 to 10 times the estimated value in Table S2 for; A) G1 phase: 1 M NaCl is applied at $t = 20$ minutes, B) S phase: 1 M NaCl is applied at $t = 50$ minutes, C) DNA replication window: 1 M NaCl is applied at $t = 75$ minutes, D) M phase: 1 M NaCl is applied at $t = 90$ minutes. See section 3 for further details of analysis. The y-axis shows $\frac{\Delta_\tau}{|\tau_o|}$ and the x-axis represents the sets of randomly chosen parameters (100 sets for each phase). The red dots represent the cells which are not dividing with the given set of parameters. They are set to -1.5 for visualisation purposes.

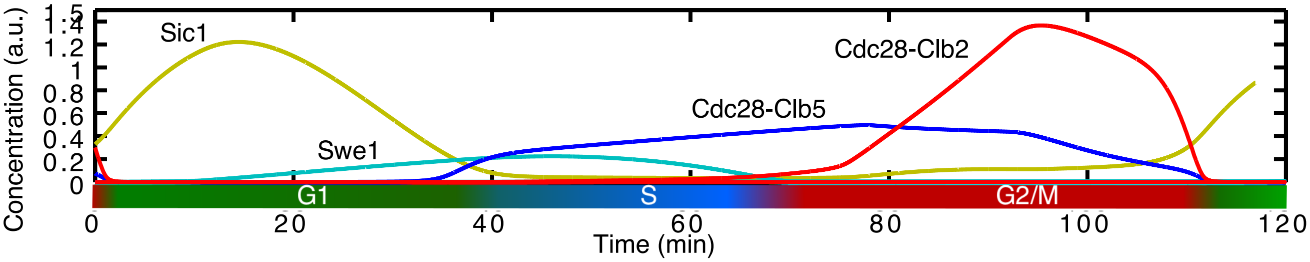

Figure S 9. Mathematical definition of the cell cycle phases. The G1 phase starts immediately after cell division, and finishes when the level of Cdc28-Clb5 crosses the level of Sic1, which indicates initiation of DNA replication and the start of the S phase. The S phase finishes when the level of Swe1 is less than the level of Cdc28-Clb2. Note that we do not distinguish between G2 and M phase, as the G2 phase is very short for *S. cerevisiae*.

References

1. Surana U, Robitsch H, Price C, Schuster T, Fitch I, et al. (1991) The role of CDC28 and cyclins during mitosis in the budding yeast S. cerevisiae. Cell 65: 145–61.

2. Tyers M, Tokiwa G, Futcher B (1993) Comparison of the Saccharomyces cerevisiae G1 cyclins: Cln3 may be an upstream activator of Cln1, Cln2 and other cyclins. The EMBO Journal 12: 1955–68.

3. Amon A, Tyers M, Futcher B, Nasmyth K (1993) Mechanisms that help the yeast cell cycle clock tick: G2 cyclins transcriptionally activate G2 cyclins and repress G1 cyclins. Cell 74: 993 - 1007.

4. Dirick L, Böhm T, Nasmyth K (1995) Roles and regulation of Cln-Cdc28 kinases at the start of the cell cycle of Saccharomyces cerevisiae. The EMBO Journal 14: 4803-13.

5. Sia R, Herald H, Lew DJ (1996) Cdc28 tyrosine phosphorylation and the morphogenesis checkpoint in budding yeast. Molecular Biology of the Cell 7: 1657–66.

6. Toyn J, Johnson A, Donovan J, Toone W, Johnston L (1997) The Swi5 transcription factor of Saccharomyces cerevisiae has a role in exit from mitosis through induction of the cdk-inhibitor Sic1 in telophase. Genetics 145: 85-96.

7. Sia R, Bardes ES, Lew DJ (1998) Control of Swe1p degradation by the morphogenesis checkpoint. The EMBO Journal 17: 6678–88.

8. Mendenhall MD, Hodge AE (1998) Regulation of Cdc28 cyclin-dependent protein kinase activity during the cell cycle of the yeast Saccharomyces cerevisiae. Microbiology and Molecular Biology Reviews 62: 1191-1243.

9. Schwob E, Böhm T, Mendenhall MD, Nasmyth K (1994) The B-type cyclin kinase inhibitor p40SIC1 controls the G1 to s transition in S. cerevisiae. Cell 79: 233–44.

10. Nash P, Tang X, Orlicky S, Chen Q, Gertler FB, et al. (2001) Multisite phosphorylation of a CDK inhibitor sets a threshold for the onset of DNA replication. Nature 414: 514-521.

11. Lew DJ (2000) Cell-cycle checkpoints that ensure coordination between nuclear and cytoplasmic events in Saccharomyces cerevisiae. Current Opinion in Genetics & Development 10: 47-53.

12. Lew DJ (2003) The morphogenesis checkpoint: how yeast cells watch their figures. Current Opinion in Cell Biology 15: 648–653.

13. Theesfeld CL, Zyla TR, Bardes EGS, Lew DJ (2003) A monitor for bud emergence in the yeast morphogenesis checkpoint. Molecular Biology of the Cell 14: 3280 -3291.

14. Burton JL, Solomon MJ (2000) Hsl1p, a Swe1p inhibitor, is degraded via the anaphase-promoting complex. Molecular and Cellular Biology 20: 4614-4625.

15. Simpson-Lavy KJ, Sajman J, Zenvirth D, Brandeis M (2009) APC/C Cdh1 specific degradation of Hsl1 and Clb2 is required for proper stress responses of S. cerevisiae. Cell Cycle 8: 3006-3012.

16. Visintin R, Prinz S, Amon A (1997) CDC20 and CDH1: a family of substrate-specific activators of APC- dependent proteolysis. Science 278: 460-3.

17. Bäumer M, Braus GH, Irniger S (2000) Two different modes of cyclin Clb2 proteolysis during mitosis in Saccharomyces cerevisiae. FEBS Letters 468: 142–8.

18. Bellí G, Garí E, Aldea M, Herrero E (2001) Osmotic stress causes a G1 cell cycle delay and downregulation of Cln3/Cdc28 activity in Saccharomyces cerevisiae. Molecular Microbiology 39: 1022-35.

19. Alexander MR, Tyers M, Perret M, Craig BM, Fang KS, et al. (2001) Regulation of cell cycle progression by Swe1p and Hog1p following hypertonic stress. Molecular biology of the cell 12: 53-62.

20. Escoté X, Zapater M, Clotet J, Posas F (2004) Hog1 mediates cell-cycle arrest in G1 phase by the dual targeting of Sic1. Nature Cell Biology 6: 997-1002.

21. Clotet J, Escoté X, Adrover MA, Yaakov G, Garí E, et al. (2006) Phosphorylation of Hsl1 by hog1 leads to a G2 arrest essential for cell survival at high osmolarity. The EMBO Journal 25: 2338-46.

22. Yaakov G, Duch A, Garcí-Rubio M, Clotet J, Jimenez J, et al. (2009) The stress-activated protein kinase Hog1 mediates S phase delay in response to osmostress. Molecular Biology of the Cell 20: 3572-3582.

23. Booher RN, Deshaies RJ, Kirschner MW (1993) Properties of Saccharomyces cerevisiae wee1 and its differential regulation of p34CDC28 in response to G1 and G2 cyclins. The EMBO Journal 12: 3417-26.

24. Stegmeier F, Amon A (2004) Closing mitosis: The functions of the Cdc14 phosphatase and its regulation. Annual Review of Genetics 38: 203-232.

25. Jorgensen P, Rupees I, Sharom JR, Schneper L, Broach JR, et al. (2004) A dynamic transcriptional network communicates growth potential to ribosome synthesis and critical cell size. Genes and Development 18: 2491-2505.

26. Charvin G, Oikonomou C, Siggia ED, Cross FR (2010) Origin of irreversibility of cell cycle start in budding yeast. PLoS Biology 8: e1000284.

27. Diffley JFX (1996) Once and only once upon a time: Specifying and regulating origins of DNA replication in eukaryotic cells. Genes and Development 10: 2819-2830.

28. Dahmann C, Diffley J, Nasmyth K (1995) S-phase-promoting cyclin-dependent kinases prevent re-replication by inhibiting the transition of replication origins to a pre-replicative state. Current Biology 5: 1257-1269.

29. Hohmann S (2002) Osmotic stress signaling and osmoadaptation in yeasts. Microbiology and Molecular Biology Reviews 66: 300-372.

30. Brewster JL, De Valoir T, Dwyer ND, Winter E, Gustin MC (1993) An osmosensing signal transduction pathway in yeast. Science 259: 1760-1763.

31. Kyriakis JM, Avruch J (2001) Mammalian mitogen-activated protein kinase signal transduction pathways activated by stress and inflammation. Physiological Reviews 81: 807-869.

32. Gustin MC, Albertyn J, Alexander M, Davenport K (1998) MAP kinase pathways in the yeast Saccharomyces cerevisiae. Microbiology and Molecular Biology Reviews 62: 1264-1300.

33. Posas F, Wurgler-Murphy SM, Maeda T, Witten EA, Thai TC, et al. (1996) Yeast HOG1 MAP kinase cascade is regulated by a multistep phosphorelay mechanism in the SLN1-YPD1-SSK1 ”two-component” osmosensor. CELL 86: 865-875.

34. Posas F, Saito H (1997) Osmotic activation of the HOG MAPK pathway via Ste11p MAPKKK: Scaffold role of Pbs2p MAPKK. Science 276: 1702-1705.

35. ÓRourke SM, Herskowitz I (2002) A third osmosensing branch in Saccharomyces cerevisiae requires the Msb2 protein and functions in parallel with the Sho1 branch. Molecular and Cellular Biology 22: 4739-4749.

36. Tatebayashi K, Tanaka K, Yang HY, Yamamoto K, Matsushita Y, et al. (2007) Transmembrane mucins Hkr1 and Msb2 are putative osmosensors in the SHO1 branch of yeast HOG pathway. The EMBO Journal 26: 3521-3533.

37. Westfall PJ, Ballon DR, Thorner J (2004) When the stress of your environment makes you go HOG wild. Science 306: 1511-1512.

38. Skowyra D, Craig KL, Tyers M, Elledge SJ, Harper JW (1997) F-box proteins are receptors that recruit phosphorylated substrates to the SCF ubiquitin-ligase complex. Cell 91: 209-219.

39. Goldbeter A, Koshland DE (1981) An amplified sensitivity arising from covalent modification in biological systems. PNAS 78: 6840-6844.

40. Chen KC, Calzone L, Csikasz-nagy A, Cross FR, Novak B, et al. (2004) Integrative analysis of cell cycle control in budding yeast. Molecular Biology of the Cell 15: 3841–3862.

41. Zi Z, Liebermeister W, Klipp E (2010) A quantitative study of the Hog1 MAPK response to fluctuating osmotic stress in Saccharomyces cerevisiae. PLoS ONE 5: e9522.

42. McMillan JN, Longtine MS, Sia R, Theesfeld CL, Bardes ES, et al. (1999) The morphogenesis checkpoint in Saccharomyces cerevisiae: cell cycle control of Swe1p degradation by Hsl1p and Hsl7p. Molecular and Cellular Biology 19: 6929–39.

43. Ciliberto A, Novak B, Tyson JJ (2003) Mathematical model of the morphogenesis checkpoint in budding yeast. The Journal of Cell Biology 163: 1243–54.

44. McMillan JN, Theesfeld CL, Harrison JC, Bardes ESG, Lew DJ (2002) Determinants of Swe1p degradation in Saccharomyces cerevisiae. Molecular Biology of the Cell 13: 3560-3575.

45. Cid VJ, Shulewitz MJ, McDonald KL, Thorner J (2001) Dynamic localization of the Swe1 regulator Hsl7 during the Saccharomyces cerevisiae cell cycle. Molecular Biology of the Cell 12: 1645–69.

46. Chen KC, Csikasz-Nagy A, Gyorffy B, Val J, Novak B, et al. (2000) Kinetic analysis of a molecular model of the budding yeast cell cycle. Molecular Biology of the Cell 11: 369-391.

47. Klipp E, Nordlander B, Kröger R, Gennemark P, Hohmann S (2005) Integrative model of the response of yeast to osmotic shock. Nature Biotechnology 23: 975-982.

48. Reiser V, Ruis H, Ammerer G (1999) Kinase activity-dependent nuclear export opposes stress-induced nuclear accumulation and retention of Hog1 mitogen-activated protein kinase in the budding yeast Saccharomyces cerevisiae. Molecular Biology of the Cell 10: 1147-1161.

49. Dohrmann PR, Voth WP, Stillman DJ (1996) Role of negative regulation in promoter specificity of the homologous transcriptional activators Ace2p and Swi5p. Molecular and Cellular Biology 16: 1746-58.

50. Cross F, Archambault V, Miller M, Klovstad M (2002) Testing a mathematical model of the yeast cell cycle. Molecular Biology of the Cell 13: 52-70.

51. Su T, Follette P, ÓFarrell P (1995) Qualifying for the license to replicate. Cell 81: 825-828.

52. Zachariae W, Nasmyth K (1999) Whose end is destruction: Cell division and the anaphase-promoting complex. Genes and Development 13: 2039-2058.

53. Epstein C, Cross F (1994) Genes that can bypass the CLN requirement for Saccharomyces cerevisiae cell cycle START. Molecular and Cellular Biology 14: 2041-2047.

54. Ghaemmaghami S, Huh W, Bower K, Howson R, Belle A, et al. (2003) Global analysis of protein expression in yeast. Nature 425: 737-741.

55. Salama S, Hendricks K, Thorner J (1994) G1 cyclin degradation: The PEST motif of yeast Cln2 is necessary, but not sufficient, for rapid protein turnover. Molecular and Cellular Biology 14: 7953-7966.
